# Evaluation of WGS performance for bacterial pathogen characterization with the Illumina technology optimized for time-critical situations

**DOI:** 10.1099/mgen.0.000699

**Published:** 2021-11-05

**Authors:** Bert Bogaerts, Raf Winand, Julien Van Braekel, Stefan Hoffman, Nancy H. C. Roosens, Sigrid C. J. De Keersmaecker, Kathleen Marchal, Kevin Vanneste

**Affiliations:** ^1^​ Transversal activities in Applied Genomics, Sciensano, Brussels (1050), Belgium; ^2^​ Department of Plant Biotechnology and Bioinformatics, Ghent University, Ghent (9000), Belgium; ^3^​ Department of Information Technology, IDLab, imec, Ghent University, Ghent (9000), Belgium; ^4^​ Department of Genetics, University of Pretoria, 0001 Pretoria, South Africa

**Keywords:** real-time, Illumina, NGS, WGS, validation

## Abstract

Whole genome sequencing (WGS) has become the reference standard for bacterial outbreak investigation and pathogen typing, providing a resolution unattainable with conventional molecular methods. Data generated with Illumina sequencers can however only be analysed after the sequencing run has finished, thereby losing valuable time during emergency situations. We evaluated both the effect of decreasing overall run time, and also a protocol to transfer and convert intermediary files generated by Illumina sequencers enabling real-time data analysis for multiple samples part of the same ongoing sequencing run, as soon as the forward reads have been sequenced. To facilitate implementation for laboratories operating under strict quality systems, extensive validation of several bioinformatics assays (16S rRNA species confirmation, gene detection against virulence factor and antimicrobial resistance databases, SNP-based antimicrobial resistance detection, serotype determination, and core genome multilocus sequence typing) for three bacterial pathogens (* Mycobacterium tuberculosis *, *Neisseria meningitidis *, and Shiga-toxin producing *Escherichia coli *) was performed by evaluating performance in function of the two most critical sequencing parameters, i.e. read length and coverage. For the majority of evaluated bioinformatics assays, actionable results could be obtained between 14 and 22 h of sequencing, decreasing the overall sequencing-to-results time by more than half. This study aids in reducing the turn-around time of WGS analysis by facilitating a faster response in time-critical scenarios and provides recommendations for time-optimized WGS with respect to required read length and coverage to achieve a minimum level of performance for the considered bioinformatics assay(s), which can also be used to maximize the cost-effectiveness of routine surveillance sequencing when response time is not essential.

## Data Summary

The datasets supporting the conclusions of this study have been previously deposited in the NCBI Sequence Read Archive under BioProjects PRJNA633966 and PRJNA574887 (* E. coli *), PRJNA448994 (* N. meningitidis *), and PRJNA681718 (* M. tuberculosis *). Individual accession numbers are provided in Table S1 (available in the online version of this article). FastQC reports for datasets generated with the real-time sequencing protocol for run C are available on Figshare: https://doi.org/10.6084/m9.figshare.16691383.v1.

**Impact Statement**

The unprecedented resolution of whole-genome sequencing (WGS) has revolutionized the characterization of pathogenic bacteria. However, a WGS run using Illumina sequencing typically requires several days rendering the technology less suited for emergencies such as bacterial outbreaks or the characterization of clinical infections. Previous studies proposed a protocol to analyse Illumina WGS data in real-time (i.e. during the sequencing) to enable a faster response. In this study, we have implemented and successfully tested this protocol in three independent MiSeq runs. Additionally, we have modified previously generated sequencing datasets from various species *in silico* to determine the minimal sequencing duration, with and without the real-time analysis protocol, to obtain accurate results for several bioinformatics assays. This flexible framework provides concrete guidelines to set up time-optimized Illumina WGS experiments, substantially reducing the turnover time.

## Introduction

Whole genome sequencing (WGS) has become the reference standard for bacterial outbreak investigation and pathogen typing. WGS-based analytical methods offer a resolution unattainable with conventional molecular typing methods, enabling quick and reliable identification of infections from a common source with a much higher level of certainty. Information on the presence of genomic features associated with antimicrobial resistance (AMR) or virulence of relevance for rapid outbreak management or clinical intervention can be extracted directly from WGS data, thereby avoiding the need for multiple molecular assays to characterize an isolate. Consequently, since it was introduced in public health settings, WGS has proven its added value for outbreak investigation [1–4] and pathogen typing [5, 6].

Time is a crucial factor in the successful implementation of infection control measures to contain and combat outbreaks. While technological advancements have substantially reduced sequencing and analysis times, a typical full-length run on the Illumina MiSeq and HiSeq sequencing instruments takes approximately three and five days to complete, respectively [7]. Only after sequencing has been completed, data becomes available for bioinformatics analysis. Shortening the duration of sequencing could hence allow an even faster response, but is limited by the ‘massively parallel’ setup of the Illumina technology wherein all reads are being sequenced simultaneously so that they become only available at full length at the end of the sequencing run. The advent of long-read sequencing technologies, such as Oxford Nanopore Technologies (ONT), introduced the possibility to analyse data in real-time, i.e. while reads are being generated by the sequencing instrument [8, 9]. The option to generate and analyse sequencing data in real-time, renders ONT sequencing especially interesting during outbreaks, but in practice remains difficult because of several bottlenecks. First, reads produced by this technology are characterized by high read error rates, rendering them less suitable for analyses that require highly accurate basecalling, such as variant calling for SNP analysis or allele detection for cgMLST, which are of critical interest to delineate strains during outbreak situations [10, 11]. Second, many public health agencies are still actively transitioning to implementing short-read sequencing technologies in their routine activities and do not always have the necessary resources to invest in this novel technology [12, 13]. Third, most national reference laboratories (NRLs) and centres (NRCs), as well as other laboratories working under a quality system, require extensive validation to demonstrate that employed methods are ‘fit-for-purpose’ and provide high-quality results through a process of rigorous validation [14–16]. Public health authorities are still actively facing the challenge of validating bioinformatics workflows for short-read technologies, for which a consensus is emerging but many issues still need to be addressed [17]. This renders the implementation and validation of the ONT technology, which is still constantly evolving resulting in quickly changing protocols and data analytical approaches [18], not necessarily the highest priority.

Another avenue that has been explored is optimizing the Illumina sequencing platforms for rapid data generation. Quick *et al*. reduced the time for a MiSeq run to about 6 h by optimizing the sequencing and library preparation protocols, and limiting read lengths to 75 bp. Despite the highly reduced data volume, they could resolve an outbreak of *Salmonella enterica* using a SNP phylogeny-based approach [19]. An alternative approach performs analysis on the intermediary files generated by Illumina sequencers while they are still sequencing. Although no vendor support exists, binary intermediate basecalling (BCL) files generated for every cycle of the Illumina sequencing process, can be converted to FASTQ files that can be fed into bioinformatics pipelines. This method was introduced by Lambert *et al*. to characterize marker genes for toxigenic *E. coli *, providing results within a single working day [20]. Recently, a set of tools based on the same principle was developed, such as HiLive that performs k-mer based read mapping in real-time [21] and serves as the basis for PathoLive that performs metagenomics pathogen detection [22]. Other real-time applications developed based on HiLive are LiveKraken [23] and PriLive [24]. The advantage of this strategy is that reads of increasing size can be periodically analysed, progressively providing better results as read lengths increase. A major drawback, besides the lack of vendor support from Illumina, is that such methods are not exempt from requiring validation within applied public health settings. Since read length is a key characteristic influencing the quality of bioinformatics analysis, shorter read lengths result in more fragmented assemblies and are more difficult to reliably map against reference genomes [25–27]. A validated workflow for full-length read data therefore does not apply when read lengths are reduced. Coverage, i.e. the sequencing depth or the number of times the genome is sequenced, constitutes a second key characteristic, as low-coverage datasets also result in more fragmented assemblies and reduce support values for read mapping and variant calling [28, 29]. Coverage is highly intertwined with read length, and their exact relationship within the same sequencing run is determined by the total number of samples that are combined or ‘multiplexed’. Adding more samples at the same sequencing read length will decrease overall coverage per sample, as will decreasing read lengths when keeping the number of samples constant. Recommendations and guidelines on minimum coverages and read lengths remain limited and vary wildly, with one estimate stating that 40X coverage and 2×250 bp reads are required for high-quality detection of virulence genes [30], and another stating that 21 bp reads at 4X coverage are sufficient for target gene detection [20]. A systematic evaluation of read lengths and coverages for multiple pathogenic species providing recommendations on minimum requirements for reliable pathogen typing and characterization is still absent, even though this is a prerequisite during outbreaks to know how quickly (partially) generated NGS datasets can reliably be used for interpretation.

Here, we evaluated both decreasing overall run time by reducing read lengths, and analysing isolate WGS data in real-time from Illumina instruments while the sequencer is still running, enabling partially generated datasets to be fed into bioinformatics workflows for typing and characterization of pathogenic isolates when response time is essential. We performed an exhaustive performance evaluation at decreasing read lengths and coverages in function of sequencing run time to provide specific recommendations for guaranteeing a minimum required level of performance based on a previously described validation strategy [31–33], using high-quality reference datasets for three pathogens (* Neisseria meningitidis *, *Mycobacterium tuberculosis *, and Shiga-toxin producing *Escherichia coli *), and evaluating multiple bioinformatics assays of public health interest that can also be adapted for other time-critical WGS applications. Specific recommendations to set up time-optimized experiments are provided through the characterization of assay performance in function of read lengths, coverage, and estimated sequencing duration.

## Methods

### Protocol for real-time data generation and analysis

**Fig. 1.**
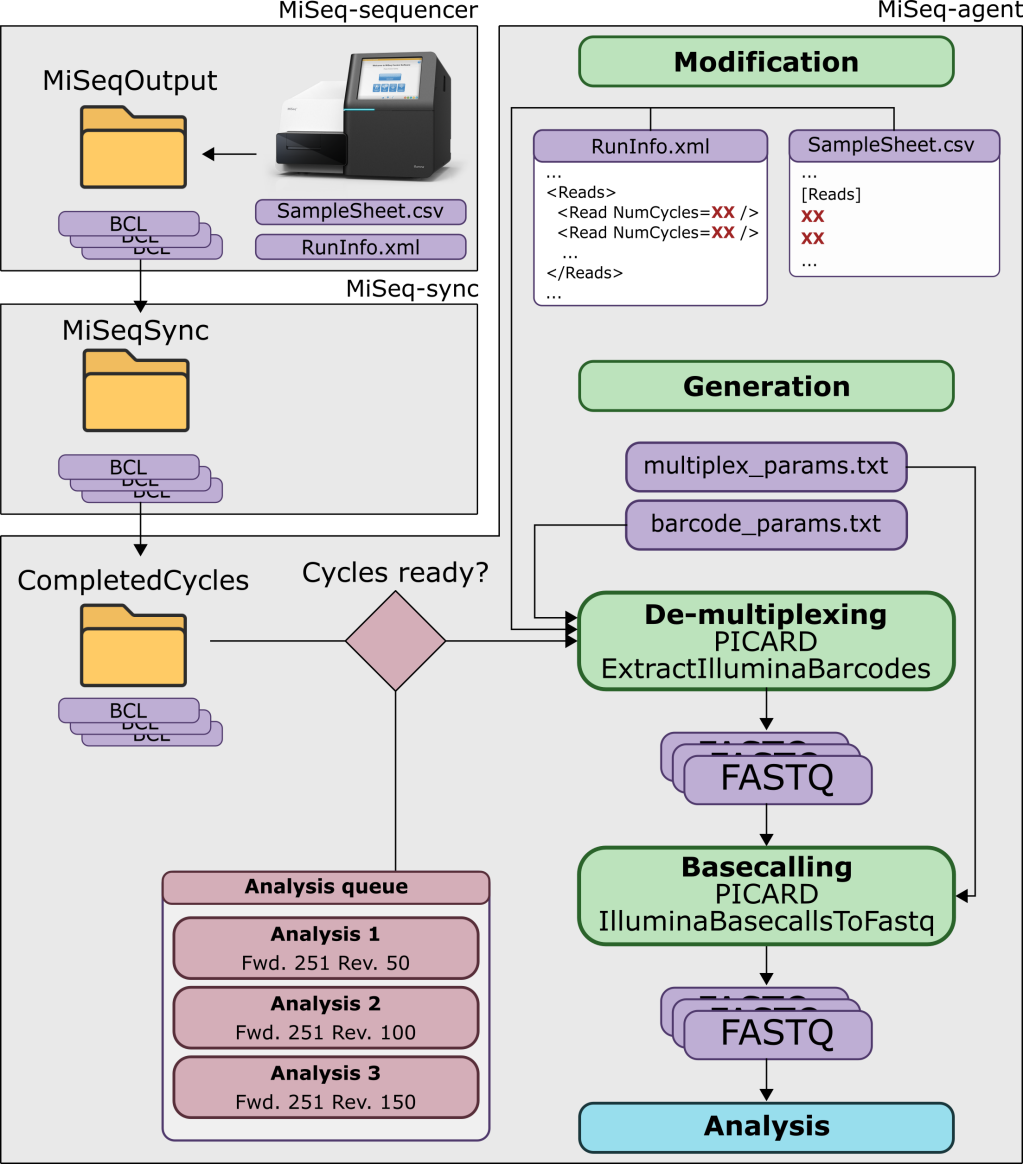
Schematic overview of the protocol for real-time data generation and analysis. The grey boxes indicate the different environments involved. The three environments are located on separate (virtual) machines or servers. See Methods S1 for a detailed description. The ‘MiSeq-sequencer’ environment corresponds to the sequencer itself. The ‘MiSeq-sync’ environment corresponds to a server that periodically mounts the MiSeq drive to transfer intermediate BCL files generated by the MiSeq. The ‘MiSeq-agent’ environment corresponds to a server that collects BCL files from the ‘MiSeq-sync' environment and converts them to FASTQ files that can be analysed with bioinformatics workflows. See Methods S1 for an elaborate description.

A schematic overview of the complete protocol is presented in Fig. 1, a full explanation is provided in the Methods S1. Concisely, a background service periodically mounts the hard drive of the MiSeq sequencer and transfers newly generated BCL files to a different network location. When sufficient data is transferred to start one of the pre-defined jobs, basecalling is performed on the analysis server (MiSeq-agent in Fig. 1) using Picard v2.8.3 (http://broadinstitute.github.io/picard/), with modified ‘RunInfo.xml’ and ‘SampleInfo.csv’ files based on the originals retrieved from the sequencer. The protocol for real-time basecalling and FASTQ data generation (Fig. 1) was tested during three independent randomly selected MiSeq sequencing runs part of the routine activities of the NGS sequencing platform at Sciensano, with each sequencing run serving as an independent technical replicate. The paired-end (PE) 251 bp protocol was used for the first two runs ‘A’ and ‘B’, and the 301 bp PE protocol for the third run ‘C’, as illustrated in Fig. S1. Data integrity of real-time basecalled datasets was verified by running FastQC for which reports have been deposited in Figshare for run C (See Data Summary). The protocol was designed and tested using the Illumina MiSeq, but can be adapted for other Illumina sequencing instruments.

### *In silico* modification of datasets

#### Validation datasets

The effects of coverage and read length on performance of various bioinformatics assays were evaluated for three species (* N. meningitidis *, *M. tuberculosis *, and *E. coli *) using high-quality data collected from several previous BioProjects (see Data Summary). These datasets were employed as validation datasets rather than the data generated during the testing of the protocol described in the previous section, because demonstrating that the protocol for real-time data generation and FASTQ generation is operational, and validation of minimal read length and coverage requirements, constitute two separate problems that do not require the same input dataset (see also Discussion). All employed WGS data were first harmonized by cropping read lengths to 251 bp with the ‘CROP’ option of Trimmomatic 0.38 [34], and then downsampled to a theoretical coverage of 70X with the ‘sample’ function of seqtk 1.2 (https://github.com/lh3/seqtk). The required number of reads for 70-fold coverage was obtained by multiplying the desired coverage with the genome size divided by the total read length (forward plus reverse read length, i.e. 502). The genome sizes for every species were taken from their corresponding entries in the NCBI RefSeq database (see Table S2) [35]. The resulting dataset is referred to as the ‘full dataset’. Performance evaluation of several assays required negative control samples, for which samples from the two other species were used (e.g. for *N. meningitidis *, *E. coli* and *M. tuberculosis* samples were used as negative controls). For some analyses, a substantial number of reads was required, for which coverage of some samples in the validation datasets did not suffice. To get the required number of reads for such samples, a ‘read-shuffling’ approach was used where new reads were derived from reads randomly sampled (with replacement) from the full dataset. When the target read length was shorter than 151 bp, random subsections of the required length were taken from the first 151 bases of the read, otherwise reads were sampled from the start of the read up to the required length. The subsections were limited to the first 151 bases because of the relatively high and constant Phred-score in this part of the reads. The starting position was varied to avoid biases by generating exactly duplicated reads. However, this approach is unusable for reads longer than 151 bp, for which the Phred-score varies and typically drops substantially at the end of the reads, so that in this case realistic error profiles could only be obtained by cropping full-length reads.

#### Decreasing coverage and read length for time-critical scenarios

**Fig. 2.**
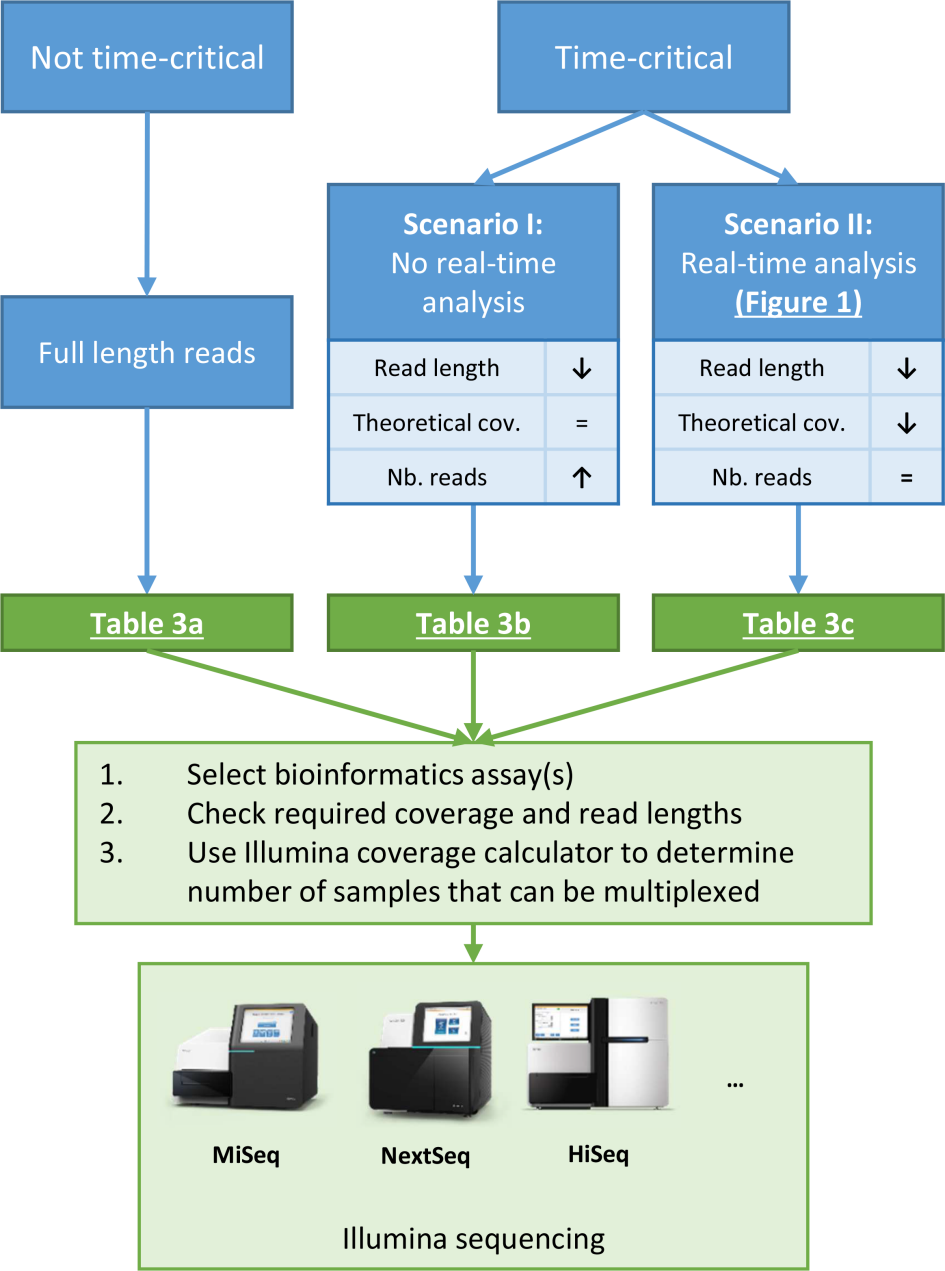
Schematic representation of the workflow to obtain minimal requirements for sequencing coverage and read lengths. The flowchart provides a schematic overview of the different scenarios for which the performance of various bioinformatics assays was evaluated. The arrows direct the steps that need to be followed to set up optimized WGS runs on Illumina sequencers. Underlined text refers to other figures or tables included in this manuscript. The ‘=’, ‘↑’, and ‘↓’ symbols indicate that the associated metric was kept constant, increased, or decreased, respectively. Abbreviations: cov, coverage. Nb., number

We evaluated first the effect of coverage when full read lengths are used for non-time-critical situations to investigate how many samples can be multiplexed in a single full sequencing run. The effects of decreasing coverage and read length were then investigated by modifying the full dataset *in silico* according to two scenarios for time-critical situations (Fig. 2). For scenario 1, we investigated how many samples can be multiplexed at lower read lengths when the real-time sequencing protocol cannot be used (for instance, when laboratories do not want to implement the protocol because their quality system does not allow changing the MiSeq configuration). In this scenario, reductions in sequencing times can be obtained by reducing the overall read length of the run. To mimic shortened read lengths, the full-length validation datasets were cropped *in silico* using the ‘CROP’ option of Trimmomatic while keeping the theoretical coverage constant by increasing the number of reads. Although the Illumina MiSeq allows read lengths up to 301 bp, we employed 251 bp as the full-length dataset because in practice the read quality drops considerably after 251 bp [36]. Because Illumina sequencers support unequal lengths for the forward and reverse read, we also evaluated setups with longer forward or reverse read lengths to characterize their effect on downstream analysis. All combinations of decreasing forward and reverse read lengths of 251, 201, 151, 126, 101, 76, and 51 bp, were evaluated at theoretical coverages of 50, 40, 30, 25, 20, 15, and 10X. A large number of reads was required to obtain high coverage at low read lengths, and therefore the ‘read-shuffling’ approach detailed above had to be used for some samples. For symmetric datasets (i.e. same forward and reverse read length), 28 samples of the corresponding species and twelve negative control samples were always analysed. For asymmetric datasets, fourteen samples of the corresponding species and six negative control samples were always analysed. Repeatability and reproducibility were always evaluated on a limited subset containing two positive and one negative control sample(s).

For scenario 2, we investigated how many samples can be multiplexed in a single full-length Illumina MiSeq sequencing run when the real-time sequencing protocol is used. Several adaptations ensure compatibility with the incomplete datasets analysed in this study, as described in the Methods S1.3. In this scenario, it is necessary to know at which point incomplete read lengths provide results of high enough quality for interpretation by the end-user. In practice, the same methodology from the first scenario was used but without correcting for the theoretical coverage by increasing read numbers. This effectively entails that the theoretical coverage is lower at lower read lengths but then progressively increases as read lengths increase to the full theoretical coverage by the end of the sequencing run. A complete overview of all samples used for both scenarios is provided in Table S3.

#### Impact of coverage and read length on WGS data quality

For our experimental setup, required read numbers were determined based on the theoretical equation for coverage, but the resulting ‘real’ effective coverage was expected to be lower as it is affected by read trimming, unmapped reads, and other factors (see also Discussion). The effective coverage was estimated by calculating the median depth per position using samtools depth 1.9 [37] after mapping the trimmed reads against the H37Rv [38] reference genome for the *M. tuberculosis* workflow, and against the assembled contigs for the *E. coli* and *N. meningitidis* workflows. The quality of the generated assemblies was evaluated using the total assembly length and N50 metrics determined with Quast 4.4 using default settings [39].

### Validation of minimal read length and coverage requirements

We built upon our previously described validation strategy to evaluate the effect of decreasing read lengths, coverages, and their interaction, on the performance of various bioinformatics assays for three different species [31–33]. An overview and short descriptions of the evaluated assays are provided in Table 1, along with references to their corresponding publications describing in detail the employed bioinformatics methodology. Additionally, more information on the exact tools and commands that were used in this study, as well as a detailed description for the pre-processing steps such as read trimming and *de novo* assembly, is available in the Supplementary Material (section S1.3). A concise description of the validation strategy is provided below and definitions for the performance metrics are provided in Table 2. The performance metrics repeatability and reproducibility were evaluated by running the bioinformatics workflow twice on the same dataset in the same and a different computational environment, respectively. The two computational environments were Python 3.7.4 on Ubuntu 18.04.3 LTS (64 bit) and Python 3.7.5 on Ubuntu 16.04.6 (64 bit). The performance metrics accuracy, precision, sensitivity, and specificity, were always evaluated by comparing results of the three bioinformatics workflows against the ‘ground truth’ for the three species, in this case results of the bioinformatics workflows obtained on the full datasets. This allows classifying all results on *in silico* modified datasets as either true positives (TP), false negatives (FN), true negatives (TN), and false positives (FP). Assay-specific definitions for TP, TN, FP, and FN, are summarized in Table 1 and explained in the Methods S2. Additionally, we employed the Matthews correlation coefficient (MCC) as an aggregate performance metric. In contrast to accuracy, the MCC is a more reliable aggregate metric that considers all confusion matrix categories and is better suited for unbalanced datasets [40]. When the MCC was undefined (i.e. when TP+FP or TN+FN was 0), its value was set to zero. For performance evaluation of some assays, negative control samples were required, which were always modified in the same manner as the positive control samples under evaluation. An *a priori* MCC value of 95 % was enforced as an acceptance criterion for considering assays to provide high-quality results.

**Table 1.** Overview of evaluated bioinformatics assays for all three species. The publication(s) describing the assay in more detail are listed in the second column, and more information about the bioinformatics methodology is also presented in the Supplementary Material Section S1.3. The primary bioinformatics tools used for the assay are listed in the third column.

Bioinformatics assay		Short description	Bioinformatics tool(s)	Evaluated species			Assay-specific definitions			
				* E. coli *	* N. meningitidis *	* M. tuberculosis *	TP	FN	TN	FP
16S rRNA species confirmation		Confirmation that the targeted species is present trough alignment of the assembled contigs against the NCBI 16S rRNA database [33]	blastn [53]+in-house workflow	No	No	Yes	Matching species detected with full dataset	Unmatched species detected with full dataset	No detection of targeted species in negative control sample	Detection of targeted species in negative control sample
Gene detection	Virulence genes	Detection of genes associated with virulence using an alignment-based approach [31]	blastn [53]+in-house workflow	Yes	Yes	No	Detection of a gene detected in full dataset	No detection of a gene detected in full dataset	No detection of a gene not detected in full dataset	Detection of a gene not detected in full dataset
	AMR genes	Detection of genes associated with AMR using an alignment-based approach [31]		Yes	No	No				
SNP-based antimicrobial resistance detection		Detection of SNPs with a known association with AMR using a read mapping-based approach [33]	SAMtools [37]+BCFtools [54, 55]	No	No	Yes	Detection of mutation present in full dataset	No detection of mutation present in full dataset	Mutation from database not detected in full and modified datasets	Mutation from database detected in modified dataset but not in full dataset
PointFinder		Detection of SNPs with a known association with AMR using an alignment-based approach [31, 33]	PointFinder [56]	Yes	No	Yes	Detection of mutation present in full dataset	No detection of mutation present in full dataset	Mutation from database not detected in full and modified datasets	Mutation from database detected in modified dataset but not in full dataset
Serotype determination*		In silico serotyping based on alignment-based detection of serotype-determining genes [31, 32]	blastn [53]+in-house workflow	Yes	Yes	No	Detection of same serotype as in full dataset	Detection of different serotype as in full dataset	No detection of serotype in negative control sample	Detection of serotype in negative control sample
Sequence typing (cgMLST)		Alignment-based detection of alleles from species-specific cgMLST schemes [31–33]	blastn [53]+in-house workflow	Yes	Yes	Yes	Detection of same allele as in full dataset	Detection of different allele as in full dataset	No detection of allele in negative control sample	Detection of allele in negative control sample

* Algorithm for serotype determination is different for *E. coli* and *N. meningitidis*.

AMR, antimicrobial resistance; FN, false negative; FP, false positive; TN, true negative; TP, true positive.

**Table 2.** Overview of performance metrics and their corresponding definitions and formulas adopted for our validation strategy

Metric	Definition	Formula
Repeatability	Agreement of assay based on intra-assay replicates*	Repeatability=100 %×(# intra-assay replicates in agreement) / (total # intra-run replicates)
Reproducibility	Agreement of assay based on inter-assay replicates*	Reproducibility=100 %×(# inter-assay replicates in agreement) / (total # inter-assay replicates)
Accuracy	The likelihood that results of the assay are correct	Accuracy=100 %×(TP+TN)/(TN+FN+TP+FP)
Precision	The likelihood that detected results of the assay are truly present	Precision=100 %×TP/(TP +FP)
Sensitivity	The likelihood that a result will be correctly picked up by the assay when present	Sensitivity=100 %×TP/(TP +FN)
Specificity	The likelihood that a result will not be falsely picked up by the assay when not present	Specificity=100 %×TN/(TN +FP)
Matthews correlation coefficient (MCC)	Compound performance metric that considers all confusion matrix categories (TN, TP, FP, FN) expressed as a value between zero (very low performance) and 1 (very high performance)	$MCC=\frac{TP.TN-FP.FN}{\sqrt{\left(TP+FP\right).\left(TP+FN\right).\left(TN+FP\right).\left(TN+FN\right)}}$

*Intra- and inter-assay replicates were defined as repeated bioinformatics analysis on the same dataset on the same and different computational environments, respectively.

### Estimation of minimal sequencing time

The performance of various bioinformatics assays was systematically evaluated in function of coverage and read length. The following equation was then used to estimate sequencing duration for each evaluated combination of coverage and read length for all datasets: T_s_=T_fixed_+ L_f_ . C_f_ + L_r_ . C_r_, where T_fixed_ represents the duration of the initial setup and sequencing of the forward and reverse adapters, C_f_ and C_r_ the average sequencing duration of a single cycle of the forward and reverse read, respectively, and L_f_ and L_r_ the forward and reverse read length, respectively (see Fig. S1). Parameter values were set to the average of the values observed during the tests of the protocol. The combination of read lengths for each assay and target coverage with the shortest estimated sequencing duration producing high-quality results (MCC ≥95 %), was then determined through an exhaustive search by considering all possible combinations and selecting the one with the shortest time requirement.

## Results

### Protocol for real-time data generation and analysis

For all three replicate runs (A, B, C), to test the real-time analysis protocol, predefined basecalling and FASTQ generation jobs finished successfully and without any interference on the sequencing run itself. A schematic overview of timelines for all three replicates is provided in Fig. S1, and exact timestamps for all cycles are provided in Tables S4–S6 for replicates A, B, and C, respectively. Basecalling jobs took around 9 min to complete averaged over the three tests of the real-time analysis protocol. The total time required to generate all sequencing cycles was 45 h and 29 min (2×251 bp PE reads), 46 h and 38 min (2×251 bp PE reads), and 55 h and 7 min (2×301 bp PE reads) for replicates A, B and C, respectively. The first basecalling jobs (i.e. after adapters had been sequenced and forward reads could be demultiplexed) were completed after 25 h and 55 min (55.29 % sequencing time), 26 h and 8 min (56.04 % sequencing time), and 30 h and 39 min (54.72 % sequencing time) for replicates A, B and C, respectively.

### Impact of coverage and read length on WGS data quality

**Fig. 3.**
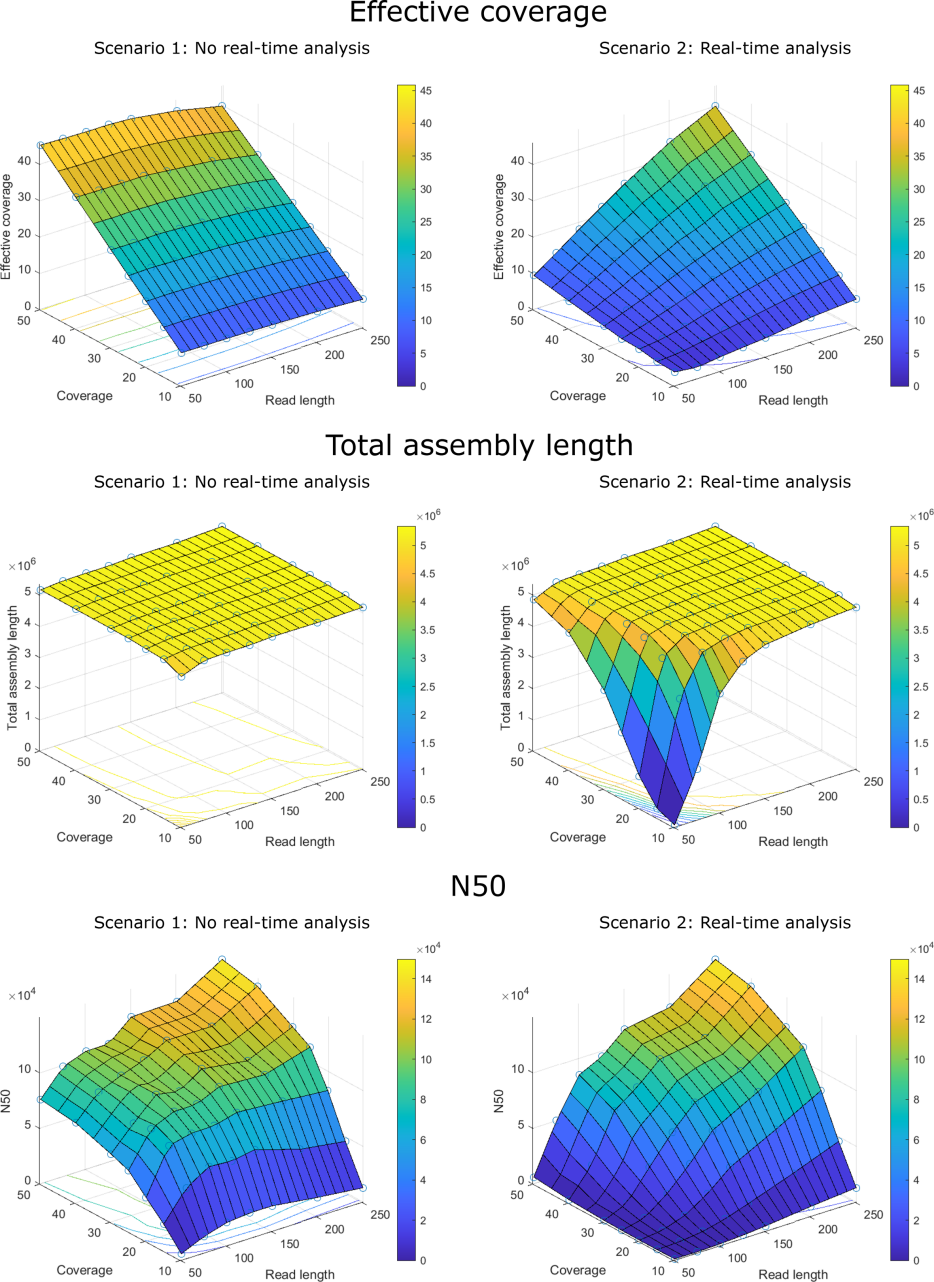
WGS data quality metrics in function of coverage and read length for *E. coli.* The x- and y-axes in each panel denote the (symmetric) read length and theoretical coverage. For scenario 2, the theoretical coverage is calculated based on full-length reads and the effective coverage is therefore lower than indicated on the y-axis. The z-axis and color-scale show the value of the corresponding metric for (a) effective coverage, (b) total assembly length, and (c) N50. The evaluated coverage and read length combinations are indicated with blue open circles, and the three-dimensional planes were extrapolated based on the observed values. Contour lines on the bottom of individual figures are indicated according to the colour legend. Figures for all three species are provided in Figs S2–S7.

The impact of coverage and read length on WGS data quality was evaluated according to two scenarios (Fig. 2). For scenario 1, the real-time analysis protocol is not used and sequencing time is reduced by sequencing shorter read lengths and keeping the theoretical coverage fixed by increasing read numbers. Scenario 2 represents the application of the real-time analysis protocol, where the number of reads is kept constant and theoretical coverage increases as read lengths gradually increase during the sequencing run. Fig. 3 illustrates the effect of read length and coverage, and their interaction, on effective coverage, total assembly length, and N50 for *E. coli* samples with symmetrical read lengths for both scenarios, obtained by analysing real sequencing datasets that were modified *in silico*. The plots for both scenarios also indirectly contain results for the non-time-critical scenario, which corresponds to the curves at full read length (i.e. 251 bp). For both time-critical scenarios, effective coverage was expectedly lower than the theoretical coverage for all combinations of read length and coverage. When read lengths increased above 151 bp and the theoretical coverage was kept fixed (scenario 1), effective coverage dropped slightly compared to the theoretical coverage (likely due to the decreasing read quality for longer reads). This effect was more profound for asymmetric combinations with a longer reverse read length. Total assembly length was mostly unaffected by coverage and read length for scenario 1, but dropped substantially for combinations with low starting coverages and short read lengths for scenario 2. For asymmetric read length combinations, longer forward read lengths generally had a positive effect on assembly quality. Assemblies were less fragmented (i.e. higher N50) when the read length and/or coverage increased (with the read length having a more pronounced effect), but the N50 decreased slightly for read lengths above 151 bp for scenario 1, likely again due to the decreasing read quality of longer reads, resulting in less data after read trimming. Similar trends were observed for *N. meningitidis* and *M. tuberculosis *, and are illustrated for all tested combinations (including asymmetric read length combinations) in Figs S2–S25, except for the N50 value which was close to the maximum for the shortest read lengths for *M. tuberculosis* in contrast to *E. coli* and *N. meningitidis *, potentially explained by a lower number of repeat regions in the *Mycobacterium* genome [41].

### Validation of minimal coverage and read length requirements

**Fig. 4.**
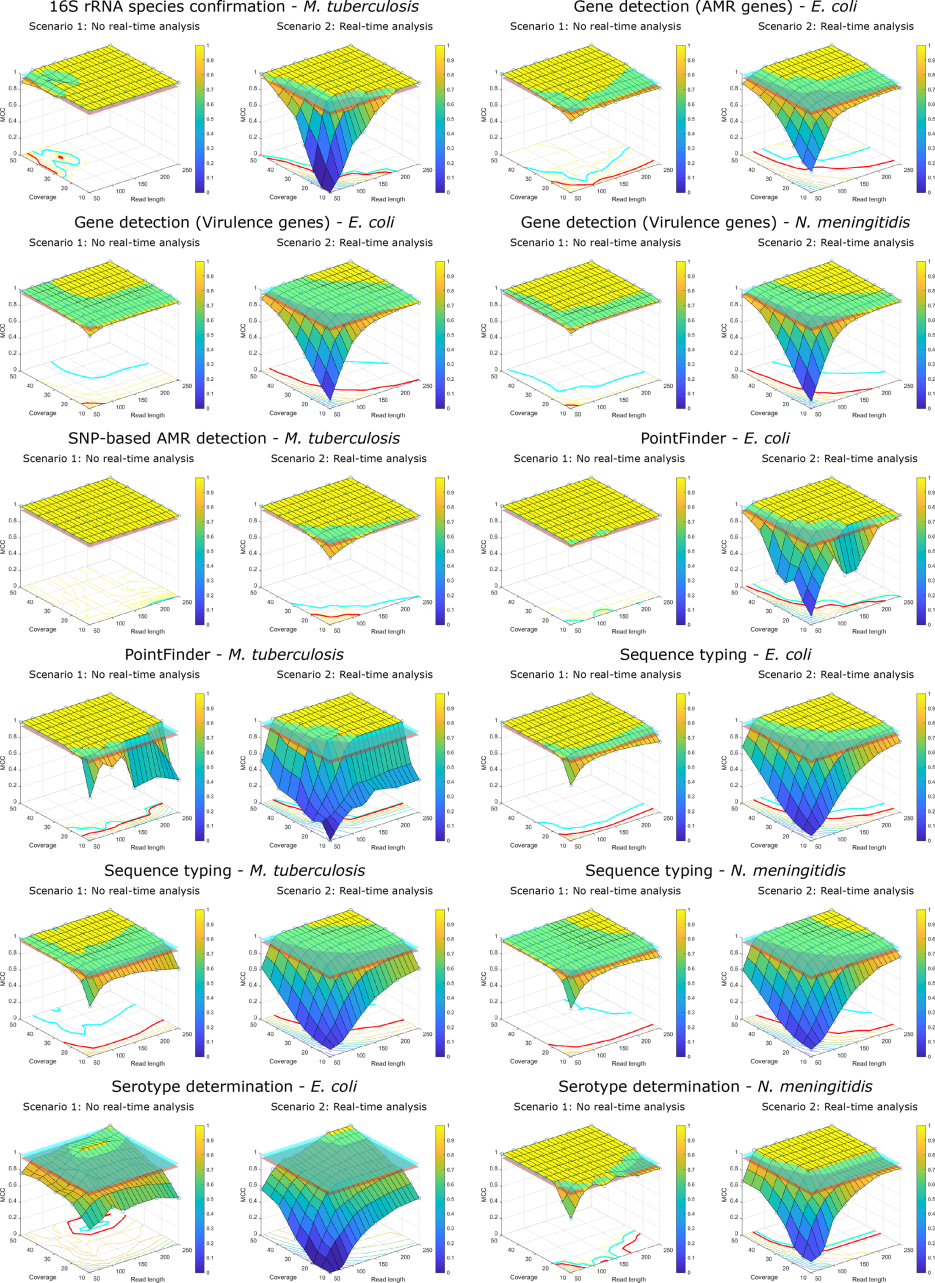
Bioinformatics assay performance in function of coverage and read length. The x- and y-axes in each panel denote the (symmetric) read length and theoretical coverage. For Scenario 2, the theoretical coverage is calculated based on full-length reads and effective coverage is therefore lower than indicated on the y-axis. The z-axis denotes the Matthews Correlation Coefficient (MCC). The evaluated data points are indicated with blue open circles, and the three-dimensional planes were extrapolated based on the observed values. The red and cyan planes correspond to MCC thresholds of 95 and 99 %, respectively. Contour lines on the bottom of individual figures are indicated according to the colour legend, and the red and cyan lines correspond to the 95 and 99% MCC thresholds, respectively. Abbreviations: antimicrobial resistance (AMR).

Results for all evaluated assays are visualized in Fig. 4 where performance thresholds defined by MCC cutoffs of 95 and 99 % are also indicated. Results for all combinations (including asymmetric read lengths) are provided in Figs S26–S65. The minimal coverage requirements for each assay when using full read lengths are provided in Table 3(a). For all assays, performance was mainly impacted by coverage, and to a lesser degree, read length. For asymmetric read length combinations, longer forward read lengths generally resulted in better performance, although the effect decreased at higher coverages. Results of replicate runs on the same and different computational environments were fully consistent, resulting in repeatability and reproducibility of 100 % for all assays. All results presented below are based on symmetric read lengths. For scenario 1, the indicated coverage on Fig. 4 was always fixed at all read lengths by increasing read numbers. For scenario 2, the indicated coverage corresponds to the theoretical coverage at full read lengths so that lower read lengths effectuate decreased effective coverages, for which the relationship is expressed in Fig. 3(a) for *E. coli* and Fig. S5 for all three species.

**Table 3.** Overview of the minimal sequencing duration and associated sequencing set-ups. Dashes indicate that performance for the corresponding theoretical coverage was below the 95 % MCC threshold for all read length combinations. For non-time-critical situations, the number of samples that can be multiplexed for the targeted assay(s) can be determined using the Lander/Waterman equation and the minimum coverage values listed in section A. For time-critical situations, the number of samples can be determined similarly, using the values in sections B and C. The minimum read-lengths that need to be sequenced are provided in section B. When the real-time sequencing protocol is used, the earliest combination that provides accurate results is provided in section C. Note that for Scenario 2, the indicated coverage is based on full-length 2×251 reads, which should be considered when determining the number of samples to multiplex in a run.

Bioinformatics assay			16S rRNA	Gene detection			SNP-based AMR detection	PointFinder		Sequence typing			Serotype determination	
**Database**			**NCBI 16S**	**NDARO**	**VFDB**		**In-house**	**PointFinder**		**EnteroBase**	**PubMLST**	**PubMLST**	**SerotypeFinder**	**PubMLST**
**Species**			*** M. tuberculosis ***	*** E. coli ***	*** E. coli ***	*** N. meningitidis ***	*** M. tuberculosis ***	*** M. tuberculosis ***	*** E. coli ***	*** E. coli ***	*** M. tuberculosis ***	*** N. meningitidis ***	*** E. coli ***	*** N. meningitidis ***
**A) Minimal coverage at full read length (2×251 bp)**														
**Theoretical coverage**			10X	15X	15X	10X	10X	10X	20X	15X	20X	15X	50X	25X
**B) Scenario 1**														
**Minimal sequencing duration**	**10X**	**Time (h**)	14.07	–	25.30	19.68	14.07	14.07	25.30	–	–	–	–	19.68
		**Read setup**	51F - 51R	–	201F - 51R	126F - 51R	51F - 51R	51F - 51R	201F - 51R	–	–	–	–	126F - 51R
	**20X**	**Time (h**)	14.07	21.56	14.07	14.07	14.07	14.07	14.07	14.07	19.68	19.68	–	14.07
		**Read setup**	51F - 51R	151F - 51R	51F - 51R	51F - 51R	51F - 51R	51F - 51R	51F - 51R	51F - 51R	126F - 51R	126F - 51R	–	51F - 51R
	**30X**	**Time (h**)	14.07	14.07	14.07	14.07	14.07	14.07	14.07	14.07	14.07	14.07	–	14.07
		**Read setup**	51F - 51R	51F - 51R	51F - 51R	51F - 51R	51F - 51R	51F - 51R	51F - 51R	51F - 51R	51F - 51R	51F - 51R	–	51F - 51R
	**40X**	**Time (h**)	14.07	14.07	14.07	14.07	14.07	14.07	14.07	14.07	14.07	14.07	30.42	14.07
		**Read setup**	51F - 51R	51F - 51R	51F - 51R	51F - 51R	51F - 51R	51F - 51R	51F - 51R	51F - 51R	51F - 51R	51F - 51R	151F - 151R	51F - 51R
	**50X**	**Time (h**)	14.07	14.07	14.07	14.07	14.07	14.07	14.07	14.07	14.07	14.07	46.77	14.07
		**Read setup**	51F - 51R	51F - 51R	51F - 51R	51F - 51R	51F - 51R	51F - 51R	51F - 51R	51F - 51R	51F - 51R	51F - 51R	251F - 251R	51F - 51R
**C) Scenario 2**														
**Minimal sequencing duratio**n	**10X**	**Time (h**)	34.16	–	–	35.69	25.30	29.04	–	–	–	–	–	29.04
		**Read setup**	201F - 151R	–	–	251F - 126R	201F - 51R	251F - 51R	–	–	–	–	–	251F - 51R
	**20X**	**Time (h**)	19.68	30.42	25.30	25.30	15.94	19.68	25.30	29.04	37.91	31.26	–	25.30
		**Read setup**	126F - 51R	151F - 151R	201F - 51R	201F - 51R	76F - 51R	126F - 51R	201F - 51R	251F - 51R	251F - 151R	251F - 76R	–	201F - 51R
	**30X**	**Time (h**)	17.81	25.30	21.56	19.68	14.07	17.81	21.56	25.30	25.30	25.30	–	19.68
		**Read setup**	101F - 51R	201F - 51R	151F - 51R	126F - 51R	51F - 51R	101F - 51R	151F - 51R	201F - 51R	201F - 51R	201F - 51R	–	126F - 51R
	**40X**	**Time (h**)	17.81	25.30	19.68	17.81	14.07	15.94	19.68	21.56	25.30	21.56	–	17.81
		**Read setup**	101F - 51R	201F - 51R	126F - 51R	101F - 51R	51F - 51R	76F - 51R	126F - 51R	151F - 51R	201F - 51R	151F - 51R	–	101F - 51R
	**50X**	**Time (h**)	13.98	21.46	17.72	17.72	13.98	13.98	17.72	19.59	19.59	19.59	38.28	17.72
		**Read setup**	51F - 51R	151F - 51R	101F - 51R	101F - 51R	51F - 51R	51F - 51R	101F - 51R	126F - 51R	126F - 51R	126F - 51R	201F - 201R	101F - 51R

AMR, antimicrobial resistance ; F, forward; R, reverse.

The 16S rRNA species confirmation assay was quite resilient to decreasing coverage and read length. For scenario 1, the MCC dropped <95 % for some combinations of high coverages and short read lengths, such as 51 bp at 50X or 101 bp at 40X. The drop was caused by contig fragmentation in a single sample (on a total of 28 positive observations). For scenario 2, the MCC was ≥95 % for read lengths increasing from 76 bp at 40X to 201 bp at 10X. For both scenarios, all mismatches could be traced back to FNs caused by contig fragmentation in the assembly breaking up the 16S rRNA gene sequence of the sample.

The gene detection assay was relatively resilient to drops in coverages and read lengths, but performance was impacted differently depending on the species and employed database. Performance using the NDARO database (genes associated with AMR) was worse compared to the VirulenceFactor core database (VFDB, for genes encoding virulence factors), based on a MCC cutoff of 95%, and performance for the latter was more robust for *N. meningitidis* compared to *E. coli *. For the NDARO database, for scenario 1, the MCC dropped <95 % at 10X for all read lengths. For coverages ≥20X, the MCC was ≥95 % for all read lengths, indicating a relatively small effect of read length. For scenario 2, the MCC dropped <95 % for all read lengths at 10X. For higher coverages, required read lengths to obtain a MCC ≥95 % decreased from 251 bp at 15X to 76 bp at 50X. For the VFDB database, for scenario 1, the MCC only dropped <95 % for the combination of the shortest read lengths (i.e. 51 bp) and lowest coverage (i.e. 10X) for both *E. coli* and *M. tuberculosis *. For scenario 2, similar trends as for the NDARO database were observed, but the MCC was generally slightly higher. The required read lengths to obtain a MCC ≥95 % varied between 151 bp at 15X and 76 bp at 50X for *E. coli,* and between 201 bp at 10X and 76 bp at 50X for *N. meningitidis *. Mismatches for gene detection were almost exclusively caused by contig fragmentations leading to FN results (i.e. genes not detected in the modified dataset), whereas FP results only occurred very rarely.

The SNP-based AMR detection assay for *M. tuberculosis* was the most resilient against decreasing coverages and read lengths. For scenario 1, the MCC was ≥95 % for all read lengths and coverages. For scenario 2, the MCC only dropped <95 % for read lengths shorter than 101 bp with coverages ≤20X. For these combinations, the number of FP and FN mismatches was approximately the same. For coverages ≥20X and above, the MCC was ≥95 % for all read lengths.

The PointFinder assay was generally quite robust to decreasing coverage and read length, but was impacted at lower coverages by an algorithmic artefact causing many mismatches, resulting in sharp drops of the MCC at the affected data points. Performance for *E. coli* was generally better than for *M. tuberculosis *. For scenario 1, the MCC never dropped <95 % for *E. coli *. For *M. tuberculosis,* the MCC was ≥95 % for coverages ≥20X for all read lengths. For scenario 2, performance was problematic for coverages <20X and/or read lengths <101 bp for both species. For all other combinations, the MCC was above the 95 % threshold.

Results of sequence typing varied for the three species but were less profound compared to gene detection. Performance was slightly worse for *M. tuberculosis* than *E. coli* and *N. meningitidis *, for which performance was comparable based on a MCC threshold of 95 %. However, at a MCC threshold of 99%, a different trend was observed with performance of the *E. coli* dataset being the most robust. For scenario 1, the MCC was ≥95 % for coverages ≥20X for all three species at all read lengths. For scenario 2, read lengths of 76 bp were required at 50X, and had to be increased to 251 bp at 20X to obtain a MCC ≥95 % for *M. tuberculosis *. For *E. coli* and *N. meningitidis,* a minimal coverage ≥15X was required to obtain a MCC ≥95 %, while for *M. tuberculosis* read lengths of ≥201 bp and at least 20X were required.

The performance of the serotype determination assays was substantially affected by decreases in coverages and read lengths. This was most apparent for *E. coli *, where the MCC dropped <95 % for all coverages <40X for both scenarios, almost exclusively due to mismatches in the detection of the O-type determining genes. For scenario 1, the MCC was only ≥95 % for the highest coverages and read lengths ≥151 bp. For scenario 2, the MCC was only ≥95 % for the highest evaluated coverages and read lengths, indicating that high coverage was required to obtain accurate results for this assay. Serotype determination for *N. meningitidis* was however more resilient. For scenario 1, the MCC dropped <95 % for coverages ≤20X for read lengths at the outer extremities (i.e. ≤126 bp or ≥201 bp). For scenario 2, similar trends as for gene detection were observed, although a coverage ≥25X and read-lengths ≥101 bp were required to obtain a MCC ≥95 %. For all coverages ≥25X, all read lengths >101 bp resulted in MCC values ≥95 %.

### Estimation of minimal sequencing time duration

On average, based on observations during replicate runs A, B, and C (Fig. S1), the fixed time for each sequencing run was 344 min, consisting of 214, 79, and 51 min for the initial setup, forward adapter and reverse adapter sequencing, respectively. A single cycle took 4.49 and 5.32 min on average for the forward and reverse read, respectively. This resulted in the following equation to estimate sequencing time: T_s_=344 min+L_f_ . 4.49 min+L_r_ . 5.32 min. The selected read lengths (i.e. L_f_ and L_r_) for the shortest estimated sequencing time with MCC values ≥95 % for each assay and targeted coverage are provided in Table 3(b, c) for scenarios 1 and 2, respectively. For the large majority of cases, the minimal sequencing time was achieved by selecting a longer forward read length followed by a shorter reverse read length, in contrast to using a shorter forward read length followed by a longer reverse read length that would allow quicker de-multiplexing because the MCC value was negatively impacted by the lower quality of the reverse read.

## Discussion

We report here an extensive validation to provide guidelines for minimal coverages and read lengths to ensure suitable performance when setting up regular, and real-time analysis, Illumina sequencing runs, to provide a quick response in time-critical situations. The first scenario considers decreasing sequencing time by reducing read lengths so that sequencing runs complete quicker, while the second scenario considers analysing data in real-time as the sequencing run is ongoing. The word ‘real-time’ can be used differently depending on context. Some studies use ‘real-time’ to refer to analyses performed while an outbreak is actively ongoing, in contrast to retrospective studies conducted after an outbreak has been resolved. We employ ‘real-time’ here to refer to analyses performed while the sequencing process is still ongoing. This concept gained acclaim with the advent of ONT sequencers offering this functionality. Although no vendor support exists for the Illumina technology, raw data can be transferred during the sequencing process and analysed in real-time [20, 21, 42]. This can provide actionable results after ~one day of sequencing when multiple isolates are multiplexed (Fig. S1), saving 1–1.5 days of sequencing time. The main bottleneck is the requirement of a de-multiplexing step, for which adapters ligated to the end of the forward and beginning of the reverse read first need to be sequenced. To the best of our knowledge, a protocol to ligate adapters to the beginning of the forward read allowing even quicker de-multiplexing has not been reported. Since sequencing a single bacterial isolate on a MiSeq is rarely an economically viable solution and de-multiplexing is consequently required for isolate applications, we considered reducing sequencing time by not using the maximum 2×301 bp paired-end MiSeq sequencing protocol, but rather employing a sequencing set-up with shorter read lengths so that the barcodes are sequenced sooner and the response time can be further reduced. An approach where the forward read length is reduced and the reverse read is sequenced completely, appears particularly interesting for real-time sequencing. This allows de-multiplexing samples quickly (as soon as the forward read and both adapters are sequenced) so that real-time data can be generated, which is then periodically repeated with increasing reverse read lengths. We observed however that the lower quality of the reverse reads had a pronounced negative impact on assay performance, rendering it more interesting to sequence a substantial part of the forward read before switching to the reverse read (Table 3). Strategies to minimize the duration of pre-sequencing steps, such as DNA extraction and library preparation, could aid in further reducing the total turnover time.

Validated bioinformatics workflows are generally based on full-length and high-coverage read datasets. It is hence relevant to characterize bioinformatics performance of incomplete sequencing datasets because this allows to (i) obtain a better understanding of minimum coverage and read length requirements to optimize capacity of full sequencing runs to minimize the cost per sample; (ii) provide guidelines for minimum requirements of Illumina sequencing datasets; and (iii) reduce read lengths of whole sequencing runs to ensure data becomes available faster while maintaining high quality. Lambert *et al*. previously evaluated the effect of read lengths and coverage on the performance of target gene identification using simulated read datasets [20]. A similar evaluation was performed here, but employing a much more extensive approach with the following differences: (i) instead of simulated reads, our approach uses real Illumina MiSeq sequencing data where read lengths and coverages were *in silico* reduced, eliminating potential bias from the underlying read generation simulation model; (ii) the evaluation included multiple bioinformatics assays besides gene detection; (iii) performance described by Lambert *et al*. was expressed in terms of percent identity to target sequences, whereas our approach builds on a previously described validation framework with assay-specific definitions for routinely used performance metrics, taking into account positive and negative results classes for a complete characterization of assay performance; (iv) Lambert *et al*. evaluated a limited set of coverages (*n*=4) and read lengths (18 bp to 50 bp), whereas our approach covers a much larger range of sequencing depths and read lengths; and (v) our approach was evaluated using three different species whereas Lambert *et al*. only included *E. coli* data. In total, 62 640 datasets were constructed and analysed to provide this in-depth characterization.

Coverage and read lengths had a substantial effect on WGS data quality and genome assembly as illustrated in Figs 2, S2–S25. Due to unmapped reads, quality trimming and other factors, the effective coverage was slightly lower than the theoretical value, especially for longer read lengths (Fig. 3a). Chen *et al*. previously showed using real sequencing data that the N50 for *de novo E. coli* assemblies plateaus for coverages between 12–20X for various read lengths combinations (i.e. 2×200 bp, 2×250 bp, 2×300 bp) [43]. Additionally, they found that longer read lengths did not increase the N50 when this plateau was reached. We observed similar trends for *M. tuberculosis* (Fig. S3), however, for both *E. coli* and *N. meningitidis* we observed a substantial increase in contiguity (i.e. higher N50) when read lengths were increased from 201 bp to 251 bp for coverages ≥20X. However, at low theoretical coverages (<20X), an opposite trend was observed, with the N50 decreasing when read lengths were increased above 151 bp as the beneficial effect of longer reads was outweighed by their lower quality towards the end. Our results hence indicate that assembly contiguity can be improved by increasing coverage and/or read length, but the added value of longer read lengths only becomes noteworthy when coverage is sufficiently high (≥20X).

The performance impact of coverage and read length was different depending on the bioinformatics assay, targeted species, and underlying database (Figs 4, S27–S66). The effect of coverage on performance has been extensively evaluated before, but comparisons are challenging due to differences in methodology, bioinformatics tools, and validation datasets [28, 29]. In contrast, the effect of read length remains largely unexplored. For gene detection, Lambert *et al*. found that a coverage of 4X for short 21 bp reads is sufficient to cover over 95 % of the genome without significant gaps, enabling accurate target gene identification [20]. Recently, Cooper *et al*. evaluated the performance of several bioinformatics methods to detect AMR genes and serotypes for *Salmonella* using downsampled Illumina datasets [44]. Performance was impacted by the targeted genes, coverage, and employed methodology, but high accuracy was observed for coverages ≥15X, regardless of the employed bioinformatics method. We observed similar results for this assay, as the MCC only dropped <95 % for short reads at low coverages (Fig. 4), and our results suggest a minimum coverage of 10–15X when using full reads, depending on the database (Table 3a). Performance for the NDARO database was generally lower compared to VFDB, for which the exact causes are unknown, but the size of the positive and negative classes (i.e. genes that are present and not present, respectively), length and sequence of the targeted genes, and genomic location can all affect performance. For example, copy numbers of plasmid-encoded genes or %GC-content can affect the local sequencing depth [45]. Our *in silico* modification approach accounts for these effects by starting from real sequencing runs, ensuring that the downsampled datasets exhibit the same uneven coverage pattern as the original runs. However, at low coverages, this might be distorted due to stochasticity, but we assume this approach still provides more realistic coverage profiles than other strategies such as *in silico* data generation. Additionally, we found that longer reads can improve accuracy even when coverage is kept constant, for instance by reducing assembly fragmentation since this was the main source of mismatches for gene detection. Other assays depending on gene detection were impacted differently by decreasing coverage and read lengths. The 16S rRNA species confirmation of *M. tuberculosis* was very resilient against decreasing coverage and read lengths, possibly due to the conserved genomic location and relatively short target length. In contrast, serotype determination for *E. coli* was heavily impacted and only produced reliable results for the highest coverages and read lengths (i.e. 50X and 2×251 bp reads). This performance drop was almost exclusively caused by mismatches in identifying O-type determining genes located in low %GC-content regions associated with lower sequencing depth [45, 46]. Our findings suggest a minimum sequencing coverage of 50X with 2×251 bp read lengths to produce reliable results, slightly higher than the 40X previously recommended by Lindsey *et al*. for Nextera XT sequencing [30]. For *N. meningitidis *, serotype determination performance was more robust, and a minimum threshold of 25X coverage with full read lengths was recommended. To the best of our knowledge, the effect of coverage and read lengths on the performance of this assay has not been characterized, but studies have employed similar coverages as a QC threshold for the analysis of *N. meningitidis* WGS data [47]. The sequence typing assay was more markedly affected by decreasing read lengths and coverages because, in contrast to gene detection, only perfect matches with the reference allele are considered correct, and a single SNP or indel consequently results in a different allele call. Similar to gene detection, substantial differences were observed between species. SNP-based AMR detection for *M. tuberculosis* was remarkably resilient against decreasing read length and/or coverage. Performance of variant detection has been described extensively [28], and recently Bush *et al*. found high precision for various SNP detection tools even at 5X coverage on simulated error-free Illumina reads of 150 and 300 bp for various bacterial species [48]. However, as they applied depth filtering, the sensitivity drastically decreased at lower coverage. We similarly observe high precision at low coverage. Since we did not apply depth filtering, the sensitivity was still high, but both FP and FN increased drastically when coverage was lowered below a minimum coverage of 10X. The literature suggests a minimum threshold of 30X for identifying SNPs associated with AMR in *M. tuberculosis* [49], and our recommendation of 10X can be slightly increased to 20X to reduce the number of mismatches, especially in light of the clinical implications of detected mutations. However, we did not observe a decrease in mismatches when coverage was further increased from 20 to 30X. A small effect of read length was observed for long read lengths at low coverage, but even for datasets with low coverage, read lengths could be drastically reduced before the MCC dropped below 95 %. In comparison, the Pointfinder assay was less resilient. While both assays identify point mutations, PointFinder was executed using the alignment of *de novo* assembled contigs rather than read mapping, which was only added as a feature in a recent update [50], and not evaluated in this study. At very low effective coverages, the assay failed to detect the targeted loci, increasing the number of FN results. Additionally, the assay was impacted at slightly higher coverages by an algorithmic artefact (see Results S1). In the absence of both issues, performance was robust and slightly higher for *E. coli* compared to *M. tuberculosis *. We did not find previous coverage and/or read length recommendations in the literature for this assay. Lastly, for all evaluated assays, repeatability and reproducibility were always 100 %, demonstrating that results of bioinformatics workflows were unaffected by repeated runs, regardless of input data quality.

By extensively characterizing the performance of different bioinformatics assays at varying read lengths and coverages, we have provided recommendations for minimal coverage and read length requirements to maintain adequate performance for various scenarios (Fig. 2), including regular runs with full-length reads (Table 3a) and two time-critical scenarios. In the first scenario, we investigated how many samples can be multiplexed at lower read lengths when the real-time sequencing protocol cannot be used, by keeping the theoretical coverage fixed and adapting read lengths. In the second scenario, we investigated how many samples can be multiplexed in a single full-length Illumina MiSeq sequencing run, and in particular, when using the real-time sequencing protocol, at which point incomplete read lengths result in high-quality results that can be used for interpretation. For all assays, performance was primarily impacted by effective coverage and to a lesser extent also by read lengths. Consequently, by reducing the number of samples sequenced per run, sequencing depth can be increased to achieve higher quality results with shorter read lengths, since a 100 bp read length reduction corresponds to approximately 8 h of sequencing. Concrete recommendations were then determined for each assay by estimating sequencing duration in function of read length (Table 3). Since coverage has the largest performance effect, the number of samples multiplexed in a single run should first be derived using the Lander/Waterman equation or tools such as the Illumina Sequencing Coverage calculator [51]. Then for each assay, the combination of read lengths with the shortest estimated sequencing time to obtain high-quality results (MCC ≥95 %) at the envisaged coverage can be determined (Table 3). Although minimum sequencing duration varied widely between assays, for the majority, total sequencing time can be drastically reduced compared to a full-length run (~46.38 h). For all assays and databases, except serotype determination for *E. coli *, a MCC value ≥95 % was obtained after less than 14 h of sequencing by optimizing the sequencing set-up (Scenario 1, Table 3b) and 22 h with the real-time sequencing protocol (Scenario 2, Table 3c) when coverage is sufficiently high. Specific guidelines for the evaluated assays can be derived by navigating Table 3. The guidelines are based on median coverages, because this information is required to determine how many samples can be multiplexed in the same sequencing run, while taking into account coverage fluctuations. For example, the requirement with respect to median coverage for serotype determination of *E. coli* is very high due to the reduced local coverage in the region containing the O-type determining genes [45]. The required median coverage for other assays for this species not subject to this effect, is consequently lower.

We acknowledge the following constraints of our work. Firstly, full-length and high-coverage read datasets were considered as the ‘ground truth’, i.e. performance was always compared to the full isolate sequencing datasets. This decision was motivated by our principal question how performance is affected by reducing sequencing lengths and coverages compared to full datasets, but also necessitated because for many bioinformatics assays (e.g. sequence typing) no gold standard reference data are available. This effectively entails that high performance at reduced sequencing depths and/or coverages can be potentially misleading if performance of the bioinformatics assay for the full dataset is poor to begin with. Notwithstanding, this effect is expected to be minimal in our study because we specifically used bioinformatics workflows already rigorously validated previously to be of very high performance [31–33]. While we employed 95 % as a threshold to consider results as high-quality, higher cut-offs can be enforced when quality needs to be as close to that of the full dataset as possible. Results of Fig. 4 therefore also indicate requirements using a MCC threshold of 99 %. For some assays, this heavily affects minimum coverage and read-length requirements (e.g. sequence typing for *N. meningitidis *), while for others the effect was minimal (e.g. 16S rRNA species confirmation). Thirdly, performance evaluation of bioinformatics assays was limited to a single method, whereas multiple alternative tools and algorithms are available but evaluating all of them would be impossible. Other laboratories can adopt our approach to characterize performance of their own bioinformatics workflows on incomplete datasets, regardless of the underlying bioinformatics methods and targeted species. Fourthly, validation focused on characterization at the isolate level, which showed that read length and coverage requirements can be substantially reduced whilst maintaining high performance for certain assays such as sequence typing or SNP-based AMR detection. However, limited mismatches at the isolate level can have a cumulative effect on interpretation of multi-isolate relationships. Notwithstanding, although the performance of cgMLST and SNP-based AMR detection cannot be directly extrapolated to phylogenomics inference methods based on cgMLST or SNPs, our results do suggest that such methods can also attain high levels of performance for use during outbreaks. Studying the exact impact of read length and coverage on phylogenomics inference would however require an entire study on its own based on datasets with known epidemiological links [52]. Fifthly, this study employed the MiSeq, while other laboratories might rely upon different Illumina sequencers. In the context of time optimization, sequencers such as the iSeq or MiniSeq with a shorter setup time and duration per cycle could be particularly suited. Alternatively, laboratories processing large number of samples will typically use the NovaSeq and NextSeq sequencers due to their higher throughput. Notwithstanding, these alternative sequencers rely on the same sequencing chemistry, and despite being quantitatively different (i.e. different number of reads per run, shorter read lengths), they do provide qualitatively the same output (i.e. similar error profiles of the reads, subject to same biases such as GC% etc.). The minimal requirements for coverage and read length described in Table 3 therefore also apply for these other Illumina sequencers, but the time estimates should be adapted according to the time-specific durations per sequencing cycle for the different sequencers. Sixthly, the employed ‘read-shuffling’ approach to obtain enough short-length reads at high coverages could potentially overestimate certain biases also present in real sequencing datasets, such as e.g. GC-bias with a higher number of short reads showing a more uniform distribution compared to a lower number of long reads, which remain impossible to account for.

In conclusion, our work provides specific recommendations to set up sequencing experiments that facilitate rapid analysis in time-critical scenarios. We present a general framework to validate the impact of coverage and read length on the performance of various bioinformatics assays across different species, which can be extended to other use-cases. Our implementation of a real-time sequencing protocol can also be used by other labs that employ the Illumina technology. Our work facilitates the integration of WGS into routine settings by labs operating under strict quality requirements that need to provide a rapid response in emergency situations.

## Supplementary Data

Supplementary material 1
