# Supplementary material for: Evaluation of WGS performance for bacterial pathogen characterization with the Illumina technology optimized for time-critical situations
Source: Microb Genom. 2021 Nov 5;7(11):000699. doi: 10.1099/mgen.0.000699 (PMC8743554; doi:10.1099/mgen.0.000699)
Supplement: Supplementary material 1 [file mgen-7-0699-s001.pdf]

SUPPLEMENTARY

Evaluation of WGS performance for bacterial pathogen characterization with the Illumina technology optimized for time-critical situations

Methods

S1 Protocol for real-time data generation and analysis

S1.1 Workflow overview

A schematic overview of the complete workflow is presented in Figure S1. The data transfer protocol is implemented as a service written in BASH that uses rsync v3.1.2 for synchronization between different entities. The workflow comprises three different environments. First, the environment 'MiSeq-sequencer' constitutes the actual Illumina MiSeq sequencing instrument, and is provided 'as-is' by Illumina and generates the actual sequencing data. During sequencing, the MiSeq saves generated data by default in the *MiSeqOutput* folder on its D:/ drive as intermediate BCL files. Second, the environment 'MiSeq-sync' constitutes a virtual machine (VM) that periodically synchronizes with the MiSeq-sequencer with read-only permissions. At regular intervals, the 'MiSeq-sync' VM mounts the D:/ drive of 'MiSeq-sequencer' to transfer generated BCL files to a network location *MiSeqSync*. Files generated at the start of the sequencing run are transferred as soon as they become available. To minimize the risk of interfering with the sequencing process on the MiSeq, the drive is unmapped between synchronization steps. Third, the environment 'MiSeq-agent' constitutes a VM that monitors *MiSeqSync*, and will only copy completed cycles to a local drive *CompletedCycles* (a cycle is considered to be completed once the output folder for the next cycle has been created). If all of the cycles required to start one of the predefined jobs in the 'data conversion queue' have been created, a basecalling job is launched (see '2.2.2 Basecalling') after which a standardized bioinformatics analysis is started (see '2.2.3 Analysis'), finally resulting in an output report for the scientist that will follow-up the potential outbreak. When all of

the specified jobs have been completed, the 'MiSeq-agent' shuts down. Predefined jobs in the data conversion queue are started after sequencing the complete forward read, and all multiples of 50 residues for the reverse read. The synchronization service on MiSeq-sync shuts down as soon as the 'RTAComplete.txt' file is generated on MiSeq-sequencer at the end of the sequencing run. The same protocol could have been constructed based on only two environments, namely the MiSeq instrument and an environment that both transfers and analyses data coming from the MiSeq. The choice for an intermediary environment was motivated by the general lack of vendor support and specific recommendations for OS updates, security etc. by Illumina for its product, so that an environment in between both layers was added for increased stability and security.

S1.2 Basecalling

Basecalling is initiated after data has been transferred to the MiSeq-agent and a predetermined number of complete cycles specified in the data conversion queue has been reached. Basecalling in our specific context refers to the process wherein the binary intermediate files coming from the MiSeq instrument are converted into human-readable FASTQ files. Picard v2.8.3 (http://broadinstitute.github.io/picard/) is used to convert BCL files to FASTQ files. The required files for conversion are 'RunInfo.xml' and 'SampleSheet.csv' that are generated at the start of sequencing on the MiSeq, and the output folders of the completed cycles that contain the '.bcl' and '.stats' files. When working with multiplexed samples, adapters are ligated both at the end of the forward read and the start of the reverse read, and both adapters need to be sequenced before de-multiplexing can occur. The total number of minimum required cycles for multiplexed samples constitutes therefore the total forward read length plus two times the length of the adapters, which are each typically eight residues long. To allow basecalling on an incomplete dataset, copies of the original 'RunInfo.xml' and 'SampleSheet.csv' files are edited to mimic the available cycles rather than the cycles of a completed sequencing run. This requires updating the 'NumCycles' attribute of the reads entries in the 'RunInfo.xml' file, and the numbers in the 'Reads' section of the 'SampleSheet.csv' file. The information from the 'Data' section of the 'SampleSheet.csv' file is then used to construct a tabular multiplex parameters file and a tabular barcode parameter file. The multiplex parameters file contains the output file prefix, first barcode sequence, and second barcode sequence for each sample, while the barcode parameter file contains the first barcode sequence, second barcode sequence, barcode name, and library name for each sample in tabular format. Afterwards, the barcode for each read is determined using the Picard 'ExtractIlluminaBarcodes'

command setting the following options: 'MAX_NO_CALLS' set to 2; 'MIN_MISMATCH_DELTA' set to 0; 'MAX_MISMATCHES' set to 2; 'NUM_PROCESSORS' set to 8; 'LANE' set to 1; 'READ_STRUCTURE' set to '{FL}T{A}B{A}B{RL}T' where FL is the forward read length, A the adapter length and RL the reverse read length; 'BASECALLS_DIR' set to the directory containing the completed cycles; and 'BARCODE_FILE' set to the aforementioned barcode parameters file. BCL files are then converted into de-multiplexed FASTQ files using the PICARD 'IlluminaBasecallsToFastq' command setting the following options: 'LANE' set to 1; 'NUM_PROCESSORS' set to 8; 'CREATE_MD5_FILE' set to 'true'; 'READ_STRUCTURE' set to '{FL}T{A}B{A}B{RL}T'; 'MULTIPLEX_PARAMS' set to the aforementioned multiplex parameters file; and the values for the 'RUN_BARCODE', 'MACHINE_NAME' and 'FLOWCELL_BARCODE' parameters set based on the values parsed from the 'RunInfo.xml' file. This approach was designed for an Illumina MiSeq, but can be adapted for other Illumina sequencing instruments by changing their accompanying settings.

S1.3 Bioinformatics analysis

The output of the basecalling is fed into previously described bioinformatics workflows for *N. meningitidis* (1)*, M. tuberculosis* (2), and STEC (3) that perform complete isolate characterization. Data pre-processing and assembly are analogous for the three workflows and starts with read-trimming followed by *de novo* assembly, after which several quality control (QC) metrics are evaluated. For this study several adaptations were made to the workflows. Firstly, QC thresholds were disabled, as dataset quality was intentionally decreased. Secondly, SPAdes (4) was updated to version 3.13.0 (compared to 3.10.0 for the original *N. meningitidis* workflow), and the value for the '--cov-cutoff' option was changed from '10' to 'off'. And thirdly, removal of contigs smaller than 1 kb was disabled.

For *N. meningitidis*, three bioinformatics assays are evaluated. First, detection of virulence genes is performed using the VirulenceFactor core database (5), instead of searching for antimicrobial resistance (AMR) genes as described in Bogaerts et al. (see also '2.2.2 Validation strategy'). The algorithm was slightly modified by clustering all gene sequences in the database beforehand and afterwards selecting the best hit for each detected cluster based on the method for allele scoring described in Bogaerts et al. Second, for sequence typing, samples are typed based on the cgMLST scheme retrieved from PubMLST (6). Lastly, for serogroup determination, the serogroup is determined based on a combination of different capsule loci that are typed, but the minimal percentage of detected

loci for was lowered from >60% to >=50%. All employed databases were updated on 24 February 2020 (unless stated otherwise), before starting the analyses.

For STEC, four assays are evaluated. First, gene detection is evaluated as described for *N. meningitidis*, using two different databases: the VirulenceFactor core database (5) and the NDARO (7) database. Second, for detection of point mutations associated with AMR, a local installation of PointFinder (checked out from BitBucket on 27-02-2019) is used with the ‘ecoli’ database. Third, the serotype is determined based on the detection of O-type and H-type determining genes from the SerotypeFinder database (8). And fourth, for sequence typing, samples are typed using the *E. coli* cgMLST scheme retrieved from EnteroBase (9).

For *M. tuberculosis*, four assays are evaluated. First, species confirmation is performed by performing gene detection on the NCBI 16S database, retrieved on 4 July 2018. Second, two methods are used to detect mutations associated with AMR: an in-house workflow described in Bogaerts et al. and a local installation of PointFinder (10), which were evaluated separately. Third, for sequence typing, samples are typed based on the cgMLST scheme retrieved from PubMLST.

S1.4 WGS sequencing

DNA extracts were preserved at −20 °C before Nextera XT DNA library preparation (Illumina) according to the manufacturer’s instructions, and subsequently underwent MiSeq sequencing over seven sequencing runs using the MiSeq V3 chemistry (Illumina) for the production of 2x251 bp reads for runs A and B and 2 x 301 bp reads for run C.

S2 Validation of minimum read length and coverage requirements

S2.1 16S rRNA species confirmation assay

The 16S rRNA species confirmation assay was unchanged compared to the *M. tuberculosis* publication (2). As this assay is only included in the *M. tuberculosis* workflow, the two other species were not evaluated. The following assay-specific definitions for classification were employed: TP as a matching detection of *M. tuberculosis* in the modified and full datasets; FN as an unmatched detection of *M. tuberculosis* in the modified and full datasets; FP as detection of *M. tuberculosis* in negative control samples; and TN as no detection of *M. tuberculosis* in negative control samples.

S2.2 Gene detection assay

The performance of the gene detection assays was evaluated with two databases: the NDARO database for genes associated with AMR and the VirulenceFactor core database (5) for genes encoding virulence factors. The performance NDARO database was only evaluated for the STEC workflow, because the employed *N. meningitidis* and *M. tuberculosis* validation datasets contained only very few antibiotic resistance genes. Likewise, the VirulenceFactor core database was only evaluated for the STEC and *N. meningitidis* workflows. Performance of this assay was evaluated at the gene level, and the following assay-specific definitions for classification were employed: TP as genes detected in both the modified and full datasets; FN as genes not detected in the modified dataset but detected in the full dataset; FP as genes detected in the modified dataset but not detected in the full dataset; and TN as genes not detected in both the modified and full datasets.

S2.3 *M. tuberculosis* SNP-based antimicrobial resistance detection assay

The AMR detection assay was completely the same as described in Bogaerts et al. (2). Performance evaluation was limited to mutations with a known link to AMR, either sensitive of resistant. No distinction was made between high confidence mutations that passed the variant filtering steps and the other detected mutations. The following assay-specific definitions for classification were employed: TP as mutations detected in the modified and full datasets; FN as mutations detected in the full dataset not detected in the modified dataset; FP as mutations detected in the modified dataset not detected in the

full dataset; and TN as no detection of mutations from the database in both the modified and full datasets.

S2.4 PointFinder assay

The PointFinder assay was not evaluated in isolation previously due to a lack of a suitable reference standard, but is was included in the genotypic AMR characterization for the STEC workflow. As no database was available for *N. meningitidis,* performance evaluation was limited to the *M. tuberculosis* and STEC workflows, with the corresponding databases. RNA mutations were excluded from the performance evaluation, resulting in 15,979 and 49,448 putative mutations for the STEC and *M. tuberculosis* workflows, respectively. The following assay-specific definitions for classification were employed: TP as mutations detected in the modified and full datasets; FN as mutations detected in the full dataset not detected in the modified dataset; FP as mutations detected in the modified dataset not detected in the full dataset; and TN as no detection of mutations in the database in both the modified and full datasets.

S2.5 Sequence typing assay

The sequence typing assay was previously described in the publication of the STEC workflow (3). Performance of this assay was evaluated at the allele level with only perfects hits taken into consideration (i.e. full length and 100% identity). Each of the three workflows was evaluated with the corresponding cgMLST scheme. The following assay-specific definitions for classification were employed: TP as the same allele detected in both the modified and full datasets; FN as alleles were the output of the modified dataset did not match the full dataset; FP as alleles detected in the negative control samples of the modified dataset; and TN as alleles not detected in the negative control samples.

S2.6 Serogroup determination assay: *N. meningitidis* and STEC

The serogroup determination assays for *N. meningitidis* and STEC were completely the same as described in the corresponding publications of the workflows (2, 3). Even though algorithmically the assays are different, the same definitions could be applied for the validation. Performance of the assay was evaluated at the serogroup level, and the following assay-specific definitions for classification were

employed: TP as serogroups that matched between the modified and full datasets; FN as serogroups that did not match between the modified and full datasets; FP as serogroups detected in negative control samples; and TN as serogroups not detected in negative control samples.

Tables

Table S1: Overview validation samples

This table contains all the samples that were used in the validation. The first column lists the species, the second column the sample identifier. The third and fourth column list the number of reads and estimated coverage, respectively. The fifth column the accession number or numbers in case of pooled samples in the NCBI SRA archive. All the samples not belonging to species *N. meningitidis* were used as negative control samples.

Species	Sample	Nb. of reads	Accession number
E. coli	ec_328	996487	SRR11816045
E. coli	ec_567	947999	SRR11815973
E. coli	ec_569	1156207	SRR11815971
E. coli	ec_570	888788	SRR11815970
E. coli	ec_572	1157265	SRR11815968
E. coli	ec_575	846621	SRR11815966
E. coli	ec_579	950555	SRR11815972
E. coli	ec_582	898362	SRR11816098
E. coli	ec_584	873968	SRR11816088
E. coli	ec_585	900982	SRR11816097
E. coli	ec_62	821932	SRR11816076
E. coli	ec_MB3933	1041211	SRR11816042
E. coli	ec_MB3971	850846	SRR11816028
E. coli	ec_MB3978	827093	SRR11815998
E. coli	ec_MB3999	1060628	SRR11816024
E. coli	ec_TIAC1151	1033500	SRR10201457
E. coli	ec_TIAC1165	859572	SRR10201439
E. coli	ec_TIAC1169	1287955	SRR10201411
E. coli	ec_TIAC1638	902326	SRR10201407
E. coli	ec_TIAC1660	993849	SRR10201399
M. tuberculosis	mt_S19BD00282	2332493	SRR13180340
M. tuberculosis	mt_S19BD00290	1354970	SRR13180338
M. tuberculosis	mt_S19BD00373	1327406	SRR13180336
M. tuberculosis	mt_S19BD00393	2991932	SRR13180335
M. tuberculosis	mt_S19BD00457	1611721	SRR13180333
M. tuberculosis	mt_S19BD00561	4727064	SRR13180332
M. tuberculosis	mt_S19BD00646	3349715	SRR13180330
M. tuberculosis	mt_S19BD01027	1845249	SRR13180275
M. tuberculosis	mt_S19BD01090	1403181	SRR13180273
M. tuberculosis	mt_S19BD01129	1392914	SRR13180432
M. tuberculosis	mt_S19BD01217	1486235	SRR13180429
M. tuberculosis	mt_S19BD01466	1643238	SRR13180421
M. tuberculosis	mt_S19BD01749	2840894	SRR13180409
M. tuberculosis	mt_S19BD02539	1545289	SRR13180374

M. tuberculosis	mt_S19BD02751	2895915	SRR14718673
M. tuberculosis	mt_S19BD02792	1928355	SRR13180368
M. tuberculosis	mt_S19BD02912	1954813	SRR14718674
M. tuberculosis	mt_S19BD03195	2145893	SRR14718675
M. tuberculosis	mt_S19BD03271	1852302	SRR13180318
M. tuberculosis	mt_S19BD03443	2785435	SRR13180315
N. meningitidis	nm_Z1035	412030	SRR6953925
N. meningitidis	nm_Z1073	454396	SRR6953927
N. meningitidis	nm_Z1092	413413	SRR6953928
N. meningitidis	nm_Z1099	415053	SRR6953929
N. meningitidis	nm_Z4672	399743	SRR6954083
N. meningitidis	nm_Z4674	415949	SRR6954081
N. meningitidis	nm_Z4676	402209	SRR6954079
N. meningitidis	nm_Z4681	491184	SRR6954087
N. meningitidis	nm_Z4682	402043	SRR6954041
N. meningitidis	nm_Z4690	391986	SRR6954042
N. meningitidis	nm_Z4692	410081	SRR6954033
N. meningitidis	nm_Z4711	431169	SRR6954018
N. meningitidis	nm_Z5043	408568	SRR6954008
N. meningitidis	nm_Z6414	470328	SRR6953972
N. meningitidis	nm_Z6416	494430	SRR6953974
N. meningitidis	nm_Z6418	428945	SRR6953977
N. meningitidis	nm_Z6422	405822	SRR6953979
N. meningitidis	nm_Z6426	391518	SRR6953978
N. meningitidis	nm_Z6429	393893	SRR6953980
N. meningitidis	nm_Z6431	426184	SRR6953958

Table S2: Selected reference genomes to calculate coverage

The first and second column contain the species and reference name respectively. The third and fourth column contain the NCBI accession number and corresponding genome size, respectively.

Species	Reference name	Accession	Genome size (bp)
E. coli	*Escherichia coli* O157:H7 str. Sakai	NC_002695.2	5498578
M. tuberculosis	*Mycobacterium tuberculosis* H37Rv	NC_000962.3	4411532
N. meningitidis	*Neisseria meningitidis* MC58	NC_003112.2	2272360

Table S3: Samples used for validation

This table contains the samples that were used to evaluate the performance of various bioinformatics assays.. The first column lists the species, the second the read lengths combination, e.g. symmetric: (equal forward and reverse read length) and asymmetric (different forward and reverse read length). The third and fourth columns lists the evaluated and negative control samples.

**Workflow**	**Read lengths**	**Samples**	**Negative control samples**
E. coli	Symmetric	ec_328, ec_567, ec_569, ec_570, ec_571, ec_572, ec_573, ec_575, ec_576, ec_579, ec_580, ec_582, ec_584, ec_585, ec_629, ec_62, ec_MB3933, ec_MB3941, ec_MB3971, ec_MB3978, ec_MB3999, ec_MB4013, ec_TIAC1151, ec_TIAC1152, ec_TIAC1165, ec_TIAC1169, ec_TIAC1638, ec_TIAC1660	mt_S19BD00393, mt_S19BD00561, mt_S19BD00646, mt_S19BD01749, mt_S19BD02751, mt_S19BD03443, nm_Z1073, nm_Z4681, nm_Z4711, nm_Z6414, nm_Z6416, nm_Z6418
E. coli	Asymmetric	ec_328, ec_567, ec_569, ec_570, ec_572, ec_579, ec_582, ec_585, ec_MB3933, ec_MB3999, ec_TIAC1151, ec_TIAC1169, ec_TIAC1638, ec_TIAC1660	mt_S19BD00393, mt_S19BD00561, mt_S19BD00646, nm_Z4681, nm_Z6414, nm_Z6416
M. tuberculosis	Symmetric	mt_S19BD00282, mt_S19BD00290, mt_S19BD00373, mt_S19BD00393, mt_S19BD00444, mt_S19BD00457, mt_S19BD00561, mt_S19BD00646, mt_S19BD00970, mt_S19BD01003, mt_S19BD01027, mt_S19BD01090, mt_S19BD01129, mt_S19BD01204, mt_S19BD01217, mt_S19BD01466, mt_S19BD01749, mt_S19BD02539, mt_S19BD02751, mt_S19BD02792, mt_S19BD02912, mt_S19BD03195, mt_S19BD03271, mt_S19BD03295, mt_S19BD03443, mt_S19BD03617, mt_S19BD03871, mt_S19BD07348	ec_569, ec_572, ec_MB3933, ec_MB3999, ec_TIAC1151, ec_TIAC1169, nm_Z1073, nm_Z4681, nm_Z4711, nm_Z6414, nm_Z6416, nm_Z6418
M. tuberculosis	Asymmetric	mt_S19BD00282, mt_S19BD00393, mt_S19BD00457, mt_S19BD00561, mt_S19BD00646, mt_S19BD01027, mt_S19BD01466, mt_S19BD01749, mt_S19BD02751, mt_S19BD02792, mt_S19BD02912, mt_S19BD03195, mt_S19BD03271, mt_S19BD03443	ec_569, ec_572, ec_TIAC1169, nm_Z4681, nm_Z6414, nm_Z6416
N. meningitidis	Symmetric	nm_Z1035, nm_Z1073, nm_Z1092, nm_Z1099, nm_Z1275, nm_Z4664, nm_Z4667, nm_Z4671, nm_Z4672, nm_Z4673, nm_Z4674, nm_Z4676, nm_Z4681, nm_Z4682, nm_Z4683, nm_Z4690, nm_Z4692, nm_Z4693, nm_Z4711, nm_Z5043, nm_Z6414, nm_Z6416, nm_Z6418, nm_Z6422, nm_Z6426, nm_Z6429, nm_Z6431, nm_Z6432	ec_569, ec_572, ec_MB3933, ec_MB3999, ec_TIAC1151, ec_TIAC1169, mt_S19BD00393, mt_S19BD00561, mt_S19BD00646, mt_S19BD01749, mt_S19BD02751, mt_S19BD03443
N. meningitidis	Asymmetric	nm_Z1035, nm_Z1073, nm_Z1092, nm_Z1099, nm_Z4674, nm_Z4681, nm_Z4692, nm_Z4711, nm_Z6414, nm_Z6416, nm_Z6418, nm_Z6431	ec_569, ec_572, ec_TIAC1169, mt_S19BD00393, mt_S19BD00561, mt_S19BD00646

Table S4: Timestamps for real-time analysis MiSeq run 1

The first column lists the different events: start of the run, end of the run, generation of each cycle, start of each basecalling job and end of each basecalling job. The second column lists the minutes that passed since that start of the sequencing run.

**Event**	**Minutes since start**
Start run	0
Cycle 1	211
Cycle 2	211
Cycle 3	211
Cycle 4	211
Cycle 5	211
Cycle 6	211
Cycle 7	211
Cycle 8	211
Cycle 9	211
Cycle 10	211
Cycle 11	211
Cycle 12	211
Cycle 13	211
Cycle 14	211
Cycle 15	211
Cycle 16	211
Cycle 17	211
Cycle 18	211
Cycle 19	211
Cycle 20	211
Cycle 21	211
Cycle 22	211
Cycle 23	211
Cycle 24	211
Cycle 25	211
Cycle 26	212
Cycle 27	217
Cycle 28	222
Cycle 29	226
Cycle 30	231
Cycle 31	236
Cycle 32	240
Cycle 33	245
Cycle 34	250
Cycle 35	255
Cycle 36	260
Cycle 37	265
Cycle 38	269
Cycle 39	274
Cycle 40	278
Cycle 41	283
Cycle 42	288
Cycle 43	293
Cycle 44	297

Cycle 45	302
Cycle 46	307
Cycle 47	312
Cycle 48	317
Cycle 49	322
Cycle 50	326
Cycle 51	331
Cycle 52	336
Cycle 53	340
Cycle 54	345
Cycle 55	350
Cycle 56	354
Cycle 57	359
Cycle 58	364
Cycle 59	368
Cycle 60	373
Cycle 61	377
Cycle 62	382
Cycle 63	387
Cycle 64	391
Cycle 65	396
Cycle 66	401
Cycle 67	405
Cycle 68	410
Cycle 69	414
Cycle 70	419
Cycle 71	424
Cycle 72	428
Cycle 73	433
Cycle 74	438
Cycle 75	442
Cycle 76	447
Cycle 77	452
Cycle 78	456
Cycle 79	461
Cycle 80	466
Cycle 81	470
Cycle 82	475
Cycle 83	480
Cycle 84	484
Cycle 85	489
Cycle 86	494
Cycle 87	498
Cycle 88	503
Cycle 89	508
Cycle 90	513
Cycle 91	517
Cycle 92	522
Cycle 93	527
Cycle 94	532
Cycle 95	536
Cycle 96	541
Cycle 97	546
Cycle 98	551
Cycle 99	555
Cycle 100	560

Cycle 101	565
Cycle 102	569
Cycle 103	574
Cycle 104	579
Cycle 105	584
Cycle 106	588
Cycle 107	593
Cycle 108	598
Cycle 109	603
Cycle 110	607
Cycle 111	612
Cycle 112	617
Cycle 113	622
Cycle 114	626
Cycle 115	631
Cycle 116	636
Cycle 117	640
Cycle 118	645
Cycle 119	650
Cycle 120	654
Cycle 121	659
Cycle 122	664
Cycle 123	668
Cycle 124	673
Cycle 125	678
Cycle 126	683
Cycle 127	687
Cycle 128	692
Cycle 129	697
Cycle 130	702
Cycle 131	706
Cycle 132	711
Cycle 133	716
Cycle 134	721
Cycle 135	726
Cycle 136	731
Cycle 137	736
Cycle 138	740
Cycle 139	745
Cycle 140	750
Cycle 141	755
Cycle 142	760
Cycle 143	764
Cycle 144	769
Cycle 145	774
Cycle 146	779
Cycle 147	784
Cycle 148	789
Cycle 149	794
Cycle 150	799
Cycle 151	803
Cycle 152	808
Cycle 153	813
Cycle 154	818
Cycle 155	823
Cycle 156	828

Cycle 157	833
Cycle 158	837
Cycle 159	842
Cycle 160	847
Cycle 161	852
Cycle 162	857
Cycle 163	862
Cycle 164	867
Cycle 165	872
Cycle 166	877
Cycle 167	882
Cycle 168	887
Cycle 169	892
Cycle 170	897
Cycle 171	902
Cycle 172	907
Cycle 173	912
Cycle 174	917
Cycle 175	922
Cycle 176	927
Cycle 177	932
Cycle 178	937
Cycle 179	942
Cycle 180	947
Cycle 181	952
Cycle 182	957
Cycle 183	962
Cycle 184	967
Cycle 185	972
Cycle 186	977
Cycle 187	982
Cycle 188	987
Cycle 189	992
Cycle 190	997
Cycle 191	1002
Cycle 192	1007
Cycle 193	1012
Cycle 194	1018
Cycle 195	1023
Cycle 196	1028
Cycle 197	1033
Cycle 198	1038
Cycle 199	1043
Cycle 200	1049
Cycle 201	1054
Cycle 202	1059
Cycle 203	1064
Cycle 204	1069
Cycle 205	1074
Cycle 206	1079
Cycle 207	1085
Cycle 208	1090
Cycle 209	1095
Cycle 210	1100
Cycle 211	1106
Cycle 212	1111

Cycle 213	1116
Cycle 214	1121
Cycle 215	1127
Cycle 216	1132
Cycle 217	1137
Cycle 218	1142
Cycle 219	1148
Cycle 220	1153
Cycle 221	1158
Cycle 222	1164
Cycle 223	1169
Cycle 224	1174
Cycle 225	1179
Cycle 226	1185
Cycle 227	1190
Cycle 228	1195
Cycle 229	1201
Cycle 230	1206
Cycle 231	1212
Cycle 232	1217
Cycle 233	1222
Cycle 234	1228
Cycle 235	1233
Cycle 236	1238
Cycle 237	1244
Cycle 238	1249
Cycle 239	1255
Cycle 240	1260
Cycle 241	1266
Cycle 242	1271
Cycle 243	1276
Cycle 244	1282
Cycle 245	1287
Cycle 246	1293
Cycle 247	1298
Cycle 248	1304
Cycle 249	1309
Cycle 250	1315
Cycle 251	1315
Cycle 252	1363
Cycle 253	1363
Cycle 254	1363
Cycle 255	1363
Cycle 256	1363
Cycle 257	1363
Cycle 258	1365
Cycle 259	1365
Cycle 260	1441
Cycle 261	1441
Cycle 262	1441
Cycle 263	1441
Cycle 264	1441
Cycle 265	1441
Cycle 266	1448
Cycle 267	1448
Cycle 268	1546

Cycle 269	1546
Cycle 270	1546
Cycle 271	1546
Cycle 272	1546
Cycle 273	1546
Cycle 274	1546
Cycle 275	1546
Cycle 276	1546
Cycle 277	1546
Cycle 278	1546
Start basecalling (251F – 0R)	1547
Cycle 279	1547
Cycle 280	1550
Cycle 281	1554
End basecalling (251F – 0R)	1555
Cycle 282	1559
Cycle 283	1564
Cycle 284	1568
Cycle 285	1573
Cycle 286	1577
Cycle 287	1582
Cycle 288	1587
Cycle 289	1591
Cycle 290	1596
Cycle 291	1600
Cycle 292	1605
Cycle 293	1609
Cycle 294	1614
Cycle 295	1619
Cycle 296	1623
Cycle 297	1628
Cycle 298	1632
Cycle 299	1637
Cycle 300	1641
Cycle 301	1646
Cycle 302	1651
Cycle 303	1655
Cycle 304	1660
Cycle 305	1665
Cycle 306	1670
Cycle 307	1674
Cycle 308	1679
Cycle 309	1684
Cycle 310	1688
Cycle 311	1693
Cycle 312	1697
Cycle 313	1702
Cycle 314	1707
Cycle 315	1711
Cycle 316	1716
Cycle 317	1721
Start basecalling (251F - 50R)	1722
Cycle 318	1725
End basecalling (251F - 50R)	1730
Cycle 319	1730
Cycle 320	1735

Cycle 321	1739
Cycle 322	1744
Cycle 323	1749
Cycle 324	1753
Cycle 325	1758
Cycle 326	1763
Cycle 327	1767
Cycle 328	1772
Cycle 329	1777
Cycle 330	1782
Cycle 331	1786
Cycle 332	1791
Cycle 333	1796
Cycle 334	1801
Cycle 335	1805
Cycle 336	1810
Cycle 337	1815
Cycle 338	1819
Cycle 339	1824
Cycle 340	1829
Cycle 341	1833
Cycle 342	1838
Cycle 343	1843
Cycle 344	1848
Cycle 345	1852
Cycle 346	1857
Cycle 347	1862
Cycle 348	1867
Cycle 349	1871
Cycle 350	1876
Cycle 351	1881
Cycle 352	1886
Cycle 353	1890
Cycle 354	1895
Cycle 355	1900
Cycle 356	1905
Cycle 357	1909
Cycle 358	1914
Cycle 359	1919
Cycle 360	1923
Cycle 361	1928
Cycle 362	1933
Cycle 363	1938
Cycle 364	1943
Cycle 365	1948
Cycle 366	1954
Cycle 367	1958
Start basecalling (251F - 100R)	1959
Cycle 368	1963
End basecalling (251F - 100R)	1966
Cycle 369	1968
Cycle 370	1973
Cycle 371	1977
Cycle 372	1982
Cycle 373	1987
Cycle 374	1992

Cycle 375	1997
Cycle 376	2002
Cycle 377	2007
Cycle 378	2012
Cycle 379	2017
Cycle 380	2022
Cycle 381	2027
Cycle 382	2032
Cycle 383	2037
Cycle 384	2042
Cycle 385	2046
Cycle 386	2051
Cycle 387	2056
Cycle 388	2061
Cycle 389	2066
Cycle 390	2070
Cycle 391	2075
Cycle 392	2080
Cycle 393	2085
Cycle 394	2090
Cycle 395	2095
Cycle 396	2099
Cycle 397	2104
Cycle 398	2109
Cycle 399	2114
Cycle 400	2119
Cycle 401	2123
Cycle 402	2128
Cycle 403	2133
Cycle 404	2138
Cycle 405	2143
Cycle 406	2148
Cycle 407	2152
Cycle 408	2157
Cycle 409	2162
Cycle 410	2167
Cycle 411	2172
Cycle 412	2177
Cycle 413	2182
Cycle 414	2187
Cycle 415	2192
Cycle 416	2197
Cycle 417	2202
Start basecalling (251F - 150R)	2203
Cycle 418	2207
End basecalling (251F - 150R)	2210
Cycle 419	2212
Cycle 420	2217
Cycle 421	2222
Cycle 422	2227
Cycle 423	2232
Cycle 424	2237
Cycle 425	2242
Cycle 426	2247
Cycle 427	2252
Cycle 428	2257

Cycle 429	2261
Cycle 430	2266
Cycle 431	2271
Cycle 432	2276
Cycle 433	2281
Cycle 434	2286
Cycle 435	2291
Cycle 436	2296
Cycle 437	2301
Cycle 438	2306
Cycle 439	2311
Cycle 440	2316
Cycle 441	2321
Cycle 442	2327
Cycle 443	2332
Cycle 444	2337
Cycle 445	2342
Cycle 446	2347
Cycle 447	2352
Cycle 448	2357
Cycle 449	2362
Cycle 450	2368
Cycle 451	2373
Cycle 452	2378
Cycle 453	2383
Cycle 454	2388
Cycle 455	2393
Cycle 456	2399
Cycle 457	2404
Cycle 458	2410
Cycle 459	2415
Cycle 460	2421
Cycle 461	2426
Cycle 462	2431
Cycle 463	2437
Cycle 464	2442
Cycle 465	2447
Cycle 466	2452
Cycle 467	2457
Start basecalling (251F - 200R)	2458
Cycle 468	2463
End basecalling (251F - 200R)	2466
Cycle 469	2468
Cycle 470	2473
Cycle 471	2478
Cycle 472	2484
Cycle 473	2489
Cycle 474	2495
Cycle 475	2500
Cycle 476	2506
Cycle 477	2511
Cycle 478	2517
Cycle 479	2522
Cycle 480	2527
Cycle 481	2532
Cycle 482	2538

Cycle 483	2543
Cycle 484	2548
Cycle 485	2554
Cycle 486	2559
Cycle 487	2564
Cycle 488	2570
Cycle 489	2575
Cycle 490	2581
Cycle 491	2586
Cycle 492	2592
Cycle 493	2597
Cycle 494	2602
Cycle 495	2608
Cycle 496	2613
Cycle 497	2619
Cycle 498	2624
Cycle 499	2630
Cycle 500	2635
Cycle 501	2641
Cycle 502	2646
Cycle 503	2651
Cycle 504	2657
Cycle 505	2662
Cycle 506	2668
Cycle 507	2673
Cycle 508	2679
Cycle 509	2684
Cycle 510	2690
Cycle 511	2696
Cycle 512	2701
Cycle 513	2707
Cycle 514	2712
Cycle 515	2718
Cycle 516	2723
Cycle 517	2729
Cycle 518	2729
Start basecalling (251F - 250R)	2730
End basecalling (251F - 250R)	2738
Basecalling done (on MiSEQ)	2781

Table S5: Timestamps for real-time analysis MiSeq run 2

The first column lists the different events: start of the run, end of the run, generation of each cycle, start of each basecalling job and end of each basecalling job. The second column lists the minutes that passed since that start of the sequencing run.

Event	Minutes since start
Start run	0
Cycle 1	213
Cycle 2	213
Cycle 3	213
Cycle 4	213
Cycle 5	213
Cycle 6	213
Cycle 7	213
Cycle 8	213
Cycle 9	213
Cycle 10	213
Cycle 11	213
Cycle 12	213
Cycle 13	213
Cycle 14	213
Cycle 15	213
Cycle 16	213
Cycle 17	213
Cycle 18	213
Cycle 19	213
Cycle 20	213
Cycle 21	213
Cycle 22	213
Cycle 23	213
Cycle 24	213
Cycle 25	213
Cycle 26	214
Cycle 27	218
Cycle 28	222
Cycle 29	227
Cycle 30	232
Cycle 31	236
Cycle 32	241
Cycle 33	245
Cycle 34	250
Cycle 35	254
Cycle 36	259
Cycle 37	263
Cycle 38	268
Cycle 39	273
Cycle 40	277
Cycle 41	282
Cycle 42	287
Cycle 43	291
Cycle 44	296

Cycle 45	300
Cycle 46	305
Cycle 47	310
Cycle 48	314
Cycle 49	319
Cycle 50	323
Cycle 51	328
Cycle 52	333
Cycle 53	337
Cycle 54	342
Cycle 55	347
Cycle 56	351
Cycle 57	356
Cycle 58	360
Cycle 59	365
Cycle 60	370
Cycle 61	374
Cycle 62	379
Cycle 63	383
Cycle 64	388
Cycle 65	393
Cycle 66	397
Cycle 67	402
Cycle 68	407
Cycle 69	411
Cycle 70	416
Cycle 71	421
Cycle 72	425
Cycle 73	430
Cycle 74	435
Cycle 75	439
Cycle 76	444
Cycle 77	449
Cycle 78	453
Cycle 79	458
Cycle 80	463
Cycle 81	467
Cycle 82	472
Cycle 83	477
Cycle 84	481
Cycle 85	486
Cycle 86	491
Cycle 87	495
Cycle 88	500
Cycle 89	504
Cycle 90	509
Cycle 91	514
Cycle 92	518
Cycle 93	523
Cycle 94	528
Cycle 95	532
Cycle 96	537
Cycle 97	542
Cycle 98	547
Cycle 99	551
Cycle 100	556

Cycle 101	561
Cycle 102	565
Cycle 103	570
Cycle 104	575
Cycle 105	580
Cycle 106	585
Cycle 107	589
Cycle 108	594
Cycle 109	599
Cycle 110	604
Cycle 111	608
Cycle 112	613
Cycle 113	618
Cycle 114	622
Cycle 115	627
Cycle 116	632
Cycle 117	637
Cycle 118	641
Cycle 119	646
Cycle 120	652
Cycle 121	657
Cycle 122	662
Cycle 123	667
Cycle 124	672
Cycle 125	676
Cycle 126	681
Cycle 127	686
Cycle 128	691
Cycle 129	696
Cycle 130	700
Cycle 131	705
Cycle 132	710
Cycle 133	715
Cycle 134	720
Cycle 135	724
Cycle 136	729
Cycle 137	734
Cycle 138	739
Cycle 139	744
Cycle 140	749
Cycle 141	754
Cycle 142	759
Cycle 143	763
Cycle 144	768
Cycle 145	773
Cycle 146	778
Cycle 147	783
Cycle 148	788
Cycle 149	793
Cycle 150	797
Cycle 151	802
Cycle 152	807
Cycle 153	812
Cycle 154	817
Cycle 155	821
Cycle 156	826

Cycle 157	831
Cycle 158	836
Cycle 159	841
Cycle 160	846
Cycle 161	850
Cycle 162	855
Cycle 163	861
Cycle 164	866
Cycle 165	871
Cycle 166	876
Cycle 167	882
Cycle 168	887
Cycle 169	892
Cycle 170	897
Cycle 171	902
Cycle 172	908
Cycle 173	913
Cycle 174	918
Cycle 175	923
Cycle 176	929
Cycle 177	934
Cycle 178	939
Cycle 179	944
Cycle 180	950
Cycle 181	955
Cycle 182	961
Cycle 183	966
Cycle 184	971
Cycle 185	976
Cycle 186	982
Cycle 187	987
Cycle 188	993
Cycle 189	998
Cycle 190	1003
Cycle 191	1009
Cycle 192	1014
Cycle 193	1019
Cycle 194	1025
Cycle 195	1030
Cycle 196	1035
Cycle 197	1041
Cycle 198	1046
Cycle 199	1052
Cycle 200	1057
Cycle 201	1062
Cycle 202	1067
Cycle 203	1073
Cycle 204	1078
Cycle 205	1084
Cycle 206	1089
Cycle 207	1095
Cycle 208	1100
Cycle 209	1105
Cycle 210	1111
Cycle 211	1116
Cycle 212	1122

Cycle 213	1127
Cycle 214	1133
Cycle 215	1138
Cycle 216	1143
Cycle 217	1149
Cycle 218	1154
Cycle 219	1159
Cycle 220	1164
Cycle 221	1170
Cycle 222	1175
Cycle 223	1180
Cycle 224	1186
Cycle 225	1191
Cycle 226	1196
Cycle 227	1202
Cycle 228	1207
Cycle 229	1212
Cycle 230	1217
Cycle 231	1223
Cycle 232	1228
Cycle 233	1234
Cycle 234	1239
Cycle 235	1244
Cycle 236	1250
Cycle 237	1255
Cycle 238	1261
Cycle 239	1266
Cycle 240	1271
Cycle 241	1277
Cycle 242	1282
Cycle 243	1288
Cycle 244	1293
Cycle 245	1298
Cycle 246	1304
Cycle 247	1310
Cycle 248	1315
Cycle 249	1320
Cycle 250	1326
Cycle 251	1326
Cycle 252	1376
Cycle 253	1376
Cycle 254	1376
Cycle 255	1376
Cycle 256	1376
Cycle 257	1376
Cycle 258	1378
Cycle 259	1378
Cycle 260	1456
Cycle 261	1456
Cycle 262	1456
Cycle 263	1456
Cycle 264	1456
Cycle 265	1456
Cycle 266	1462
Cycle 267	1462
Cycle 268	1562

Cycle 269	1562
Cycle 270	1562
Cycle 271	1562
Cycle 272	1562
Cycle 273	1562
Cycle 274	1562
Cycle 275	1562
Cycle 276	1562
Cycle 277	1562
Cycle 278	1562
Start basecalling (251F - 0R)	1562
Cycle 279	1563
Cycle 280	1564
End basecalling (251F - 0R)	1568
Cycle 281	1569
Cycle 282	1574
Cycle 283	1578
Cycle 284	1583
Cycle 285	1587
Cycle 286	1592
Cycle 287	1597
Cycle 288	1601
Cycle 289	1606
Cycle 290	1611
Cycle 291	1615
Cycle 292	1621
Cycle 293	1625
Cycle 294	1630
Cycle 295	1634
Cycle 296	1639
Cycle 297	1644
Cycle 298	1649
Cycle 299	1653
Cycle 300	1658
Cycle 301	1663
Cycle 302	1667
Cycle 303	1672
Cycle 304	1677
Cycle 305	1681
Cycle 306	1686
Cycle 307	1691
Cycle 308	1695
Cycle 309	1700
Cycle 310	1705
Cycle 311	1710
Cycle 312	1714
Cycle 313	1719
Cycle 314	1724
Cycle 315	1728
Cycle 316	1733
Cycle 317	1738
Start basecalling (251F - 50R)	1739
Cycle 318	1742
End basecalling (251F - 50R)	1747
Cycle 319	1747
Cycle 320	1752

Cycle 321	1756
Cycle 322	1761
Cycle 323	1766
Cycle 324	1770
Cycle 325	1775
Cycle 326	1780
Cycle 327	1785
Cycle 328	1789
Cycle 329	1794
Cycle 330	1799
Cycle 331	1805
Cycle 332	1810
Cycle 333	1815
Cycle 334	1820
Cycle 335	1824
Cycle 336	1829
Cycle 337	1834
Cycle 338	1838
Cycle 339	1843
Cycle 340	1848
Cycle 341	1853
Cycle 342	1858
Cycle 343	1862
Cycle 344	1867
Cycle 345	1872
Cycle 346	1877
Cycle 347	1882
Cycle 348	1888
Cycle 349	1893
Cycle 350	1897
Cycle 351	1902
Cycle 352	1907
Cycle 353	1912
Cycle 354	1917
Cycle 355	1922
Cycle 356	1927
Cycle 357	1932
Cycle 358	1937
Cycle 359	1942
Cycle 360	1947
Cycle 361	1952
Cycle 362	1958
Cycle 363	1963
Cycle 364	1968
Cycle 365	1973
Cycle 366	1977
Cycle 367	1983
Start basecalling (251F - 100R)	1983
Cycle 368	1988
End basecalling (251F - 100R)	1990
Cycle 369	1993
Cycle 370	1998
Cycle 371	2004
Cycle 372	2008
Cycle 373	2014
Cycle 374	2019

Cycle 375	2024
Cycle 376	2029
Cycle 377	2034
Cycle 378	2039
Cycle 379	2045
Cycle 380	2050
Cycle 381	2055
Cycle 382	2060
Cycle 383	2065
Cycle 384	2070
Cycle 385	2076
Cycle 386	2081
Cycle 387	2086
Cycle 388	2091
Cycle 389	2096
Cycle 390	2101
Cycle 391	2107
Cycle 392	2112
Cycle 393	2117
Cycle 394	2123
Cycle 395	2128
Cycle 396	2133
Cycle 397	2138
Cycle 398	2143
Cycle 399	2149
Cycle 400	2154
Cycle 401	2159
Cycle 402	2164
Cycle 403	2170
Cycle 404	2175
Cycle 405	2180
Cycle 406	2185
Cycle 407	2191
Cycle 408	2195
Cycle 409	2201
Cycle 410	2206
Cycle 411	2212
Cycle 412	2217
Cycle 413	2222
Cycle 414	2227
Cycle 415	2233
Cycle 416	2238
Cycle 417	2243
Start basecalling (251F - 150R)	2244
Cycle 418	2249
End basecalling (251F - 150R)	2252
Cycle 419	2254
Cycle 420	2259
Cycle 421	2264
Cycle 422	2270
Cycle 423	2275
Cycle 424	2281
Cycle 425	2286
Cycle 426	2291
Cycle 427	2297
Cycle 428	2302

Cycle 429	2307
Cycle 430	2313
Cycle 431	2318
Cycle 432	2323
Cycle 433	2329
Cycle 434	2334
Cycle 435	2339
Cycle 436	2345
Cycle 437	2350
Cycle 438	2356
Cycle 439	2361
Cycle 440	2366
Cycle 441	2371
Cycle 442	2377
Cycle 443	2382
Cycle 444	2388
Cycle 445	2393
Cycle 446	2399
Cycle 447	2404
Cycle 448	2409
Cycle 449	2415
Cycle 450	2420
Cycle 451	2426
Cycle 452	2431
Cycle 453	2437
Cycle 454	2442
Cycle 455	2447
Cycle 456	2453
Cycle 457	2458
Cycle 458	2464
Cycle 459	2470
Cycle 460	2475
Cycle 461	2481
Cycle 462	2486
Cycle 463	2491
Cycle 464	2497
Cycle 465	2502
Cycle 466	2508
Cycle 467	2514
Start basecalling (251F - 200R)	2515
Cycle 468	2519
End basecalling (251F - 200R)	2522
Cycle 469	2525
Cycle 470	2530
Cycle 471	2535
Cycle 472	2541
Cycle 473	2547
Cycle 474	2552
Cycle 475	2558
Cycle 476	2564
Cycle 477	2570
Cycle 478	2575
Cycle 479	2580
Cycle 480	2586
Cycle 481	2592
Cycle 482	2597

Cycle 483	2603
Cycle 484	2609
Cycle 485	2614
Cycle 486	2620
Cycle 487	2626
Cycle 488	2632
Cycle 489	2638
Cycle 490	2643
Cycle 491	2649
Cycle 492	2655
Cycle 493	2660
Cycle 494	2666
Cycle 495	2672
Cycle 496	2678
Cycle 497	2684
Cycle 498	2689
Cycle 499	2695
Cycle 500	2701
Cycle 501	2707
Cycle 502	2713
Cycle 503	2718
Cycle 504	2724
Cycle 505	2730
Cycle 506	2736
Cycle 507	2742
Cycle 508	2747
Cycle 509	2753
Cycle 510	2759
Cycle 511	2764
Cycle 512	2770
Cycle 513	2776
Cycle 514	2781
Cycle 515	2787
Cycle 516	2792
Cycle 517	2798
Cycle 518	2798
Start basecalling (251F - 250R)	2799
End basecalling (251F - 250R)	2806
Basecalling done (on MiSEQ)	2850

Table S6: Timestamps for real-time analysis MiSeq run 3

The first column lists the different events: start of the run, end of the run, generation of each cycle, start of each basecalling job and end of each basecalling job. The second column lists the minutes that passed since that start of the sequencing run.

Event	Minutes since start
Cycle 1	216
Cycle 2	217
Cycle 3	217
Cycle 4	217
Cycle 5	217
Cycle 6	218
Cycle 7	218
Cycle 8	218
Cycle 9	218
Cycle 10	219
Cycle 11	219
Cycle 12	219
Cycle 13	220
Cycle 14	220
Cycle 15	220
Cycle 16	220
Cycle 17	221
Cycle 18	221
Cycle 19	221
Cycle 20	222
Cycle 21	222
Cycle 22	222
Cycle 23	223
Cycle 24	223
Cycle 25	223
Cycle 26	224
Cycle 27	224
Cycle 28	224
Cycle 29	224
Cycle 30	225
Cycle 31	225
Cycle 32	225
Cycle 33	225
Cycle 34	226
Cycle 35	226
Cycle 36	226
Cycle 37	227
Cycle 38	227
Cycle 39	227
Cycle 40	227
Cycle 41	228
Cycle 42	228
Cycle 43	232
Cycle 44	237
Cycle 45	241

Cycle 46	246
Cycle 47	250
Cycle 48	255
Cycle 49	259
Cycle 50	264
Cycle 51	269
Cycle 52	274
Cycle 53	277
Cycle 54	283
Cycle 55	289
Cycle 56	293
Cycle 57	298
Cycle 58	302
Cycle 59	306
Cycle 60	312
Cycle 61	316
Cycle 62	321
Cycle 63	325
Cycle 64	331
Cycle 65	336
Cycle 66	341
Cycle 67	345
Cycle 68	349
Cycle 69	353
Cycle 70	359
Cycle 71	363
Cycle 72	368
Cycle 73	372
Cycle 74	378
Cycle 75	382
Cycle 76	387
Cycle 77	392
Cycle 78	395
Cycle 79	400
Cycle 80	405
Cycle 81	410
Cycle 82	413
Cycle 83	418
Cycle 84	424
Cycle 85	427
Cycle 86	436
Cycle 87	437
Cycle 88	441
Cycle 89	446
Cycle 90	453
Cycle 91	455
Cycle 92	460
Cycle 93	464
Cycle 94	468
Cycle 95	473
Cycle 96	478
Cycle 97	483
Cycle 98	487
Cycle 99	492
Cycle 100	498
Cycle 101	502

Cycle 102	507
Cycle 103	511
Cycle 104	516
Cycle 105	521
Cycle 106	526
Cycle 107	530
Cycle 108	535
Cycle 109	540
Cycle 110	544
Cycle 111	548
Cycle 112	553
Cycle 113	558
Cycle 114	563
Cycle 115	569
Cycle 116	572
Cycle 117	577
Cycle 118	582
Cycle 119	588
Cycle 120	591
Cycle 121	596
Cycle 122	602
Cycle 123	606
Cycle 124	612
Cycle 125	616
Cycle 126	622
Cycle 127	625
Cycle 128	631
Cycle 129	635
Cycle 130	640
Cycle 131	644
Cycle 132	649
Cycle 133	655
Cycle 134	659
Cycle 135	665
Cycle 136	668
Cycle 137	674
Cycle 138	678
Cycle 139	683
Cycle 140	689
Cycle 141	693
Cycle 142	697
Cycle 143	703
Cycle 144	707
Cycle 145	714
Cycle 146	718
Cycle 147	722
Cycle 148	728
Cycle 149	732
Cycle 150	738
Cycle 151	742
Cycle 152	746
Cycle 153	751
Cycle 154	758
Cycle 155	760
Cycle 156	766
Cycle 157	773

Cycle 158	778
Cycle 159	782
Cycle 160	787
Cycle 161	791
Cycle 162	795
Cycle 163	800
Cycle 164	806
Cycle 165	812
Cycle 166	818
Cycle 167	820
Cycle 168	826
Cycle 169	832
Cycle 170	837
Cycle 171	842
Cycle 172	847
Cycle 173	850
Cycle 174	855
Cycle 175	861
Cycle 176	866
Cycle 177	872
Cycle 178	874
Cycle 179	880
Cycle 180	885
Cycle 181	891
Cycle 182	896
Cycle 183	901
Cycle 184	907
Cycle 185	912
Cycle 186	917
Cycle 187	920
Cycle 188	927
Cycle 189	930
Cycle 190	935
Cycle 191	943
Cycle 192	948
Cycle 193	954
Cycle 194	960
Cycle 195	965
Cycle 196	968
Cycle 197	973
Cycle 198	978
Cycle 199	983
Cycle 200	988
Cycle 201	995
Cycle 202	1000
Cycle 203	1006
Cycle 204	1012
Cycle 205	1018
Cycle 206	1024
Cycle 207	1029
Cycle 208	1035
Cycle 209	1040
Cycle 210	1043
Cycle 211	1052
Cycle 212	1054
Cycle 213	1060

Cycle 214	1065
Cycle 215	1071
Cycle 216	1077
Cycle 217	1082
Cycle 218	1089
Cycle 219	1095
Cycle 220	1101
Cycle 221	1107
Cycle 222	1110
Cycle 223	1116
Cycle 224	1122
Cycle 225	1128
Cycle 226	1134
Cycle 227	1140
Cycle 228	1143
Cycle 229	1149
Cycle 230	1156
Cycle 231	1162
Cycle 232	1169
Cycle 233	1172
Cycle 234	1178
Cycle 235	1184
Cycle 236	1187
Cycle 237	1193
Cycle 238	1199
Cycle 239	1206
Cycle 240	1209
Cycle 241	1217
Cycle 242	1223
Cycle 243	1226
Cycle 244	1233
Cycle 245	1239
Cycle 246	1242
Cycle 247	1249
Cycle 248	1256
Cycle 249	1259
Cycle 250	1266
Cycle 251	1270
Cycle 252	1278
Cycle 253	1281
Cycle 254	1288
Cycle 255	1295
Cycle 256	1298
Cycle 257	1305
Cycle 258	1309
Cycle 259	1315
Cycle 260	1322
Cycle 261	1326
Cycle 262	1333
Cycle 263	1341
Cycle 264	1344
Cycle 265	1351
Cycle 266	1355
Cycle 267	1362
Cycle 268	1368
Cycle 269	1372

Cycle 270	1379
Cycle 271	1382
Cycle 272	1390
Cycle 273	1397
Cycle 274	1401
Cycle 275	1408
Cycle 276	1411
Cycle 277	1418
Cycle 278	1426
Cycle 279	1429
Cycle 280	1437
Cycle 281	1440
Cycle 282	1449
Cycle 283	1452
Cycle 284	1460
Cycle 285	1463
Cycle 286	1471
Cycle 287	1478
Cycle 288	1482
Cycle 289	1489
Cycle 290	1493
Cycle 291	1501
Cycle 292	1505
Cycle 293	1513
Cycle 294	1517
Cycle 295	1525
Cycle 296	1529
Cycle 297	1537
Cycle 298	1541
Cycle 299	1550
Cycle 300	1550
Cycle 301	1597
Cycle 302	1597
Cycle 303	1598
Cycle 304	1598
Cycle 305	1598
Cycle 306	1598
Cycle 307	1601
Cycle 308	1602
Cycle 309	1674
Cycle 310	1675
Cycle 311	1675
Cycle 312	1675
Cycle 313	1675
Cycle 314	1676
Cycle 315	1679
Cycle 316	1679
Cycle 317	1778
Cycle 318	1778
Cycle 319	1778
Cycle 320	1779
Cycle 321	1779
Cycle 322	1779
Cycle 323	1779
Cycle 324	1780
Cycle 325	1780

Cycle 326	1780
Cycle 327	1780
Cycle 328	1783
Cycle 329	1784
Cycle 330	1787
Cycle 331	1792
Cycle 332	1797
Cycle 333	1801
Cycle 334	1805
Cycle 335	1810
Cycle 336	1814
Cycle 337	1822
Cycle 338	1826
Cycle 339	1831
Cycle 340	1836
Cycle 341	1840
Cycle 342	1845
Cycle 343	1850
Cycle 344	1854
Cycle 345	1858
Cycle 346	1863
Cycle 347	1867
Cycle 348	1871
Cycle 349	1876
Cycle 350	1881
Cycle 351	1885
Cycle 352	1891
Cycle 353	1896
Cycle 354	1900
Cycle 355	1905
Cycle 356	1909
Cycle 357	1914
Cycle 358	1918
Cycle 359	1923
Cycle 360	1927
Cycle 361	1932
Cycle 362	1936
Cycle 363	1942
Cycle 364	1947
Cycle 365	1951
Cycle 366	1956
Start basecalling (301F - 50R)	1960
End basecalling (301F - 50R)	1966
Cycle 368	1967
Cycle 369	1969
Cycle 370	1974
Cycle 371	1978
Cycle 372	1983
Cycle 373	1989
Cycle 374	1993
Cycle 375	1998
Cycle 376	2002
Cycle 377	2007
Cycle 378	2017
Cycle 379	2017
Cycle 380	2021

Cycle 381	2026
Cycle 382	2032
Cycle 383	2037
Cycle 384	2041
Cycle 385	2046
Cycle 386	2051
Cycle 387	2055
Cycle 388	2060
Cycle 389	2065
Cycle 390	2070
Cycle 391	2077
Cycle 392	2081
Cycle 393	2086
Cycle 394	2091
Cycle 395	2096
Cycle 396	2101
Cycle 397	2106
Cycle 398	2111
Cycle 399	2116
Cycle 400	2122
Cycle 401	2122
Cycle 402	2127
Cycle 403	2132
Cycle 404	2138
Cycle 405	2143
Cycle 406	2148
Cycle 407	2154
Cycle 408	2159
Cycle 409	2165
Cycle 410	2165
Cycle 411	2170
Cycle 412	2176
Cycle 413	2181
Cycle 414	2186
Cycle 415	2192
Cycle 416	2198
Start basecalling (301F - 100R)	2199
End basecalling (301F - 100R)	2205
Cycle 418	2206
Cycle 419	2211
Cycle 420	2217
Cycle 421	2217
Cycle 422	2225
Cycle 423	2231
Cycle 424	2237
Cycle 425	2237
Cycle 426	2244
Cycle 427	2255
Cycle 428	2256
Cycle 429	2268
Cycle 430	2268
Cycle 431	2278
Cycle 432	2278
Cycle 433	2289
Cycle 434	2289
Cycle 435	2299

Cycle 436	2300
Cycle 437	2300
Cycle 438	2312
Cycle 439	2312
Cycle 440	2323
Cycle 441	2324
Cycle 442	2335
Cycle 443	2335
Cycle 444	2335
Cycle 445	2346
Cycle 446	2347
Cycle 447	2359
Cycle 448	2359
Cycle 449	2359
Cycle 450	2370
Cycle 451	2370
Cycle 452	2382
Cycle 453	2382
Cycle 454	2393
Cycle 455	2393
Cycle 456	2394
Cycle 457	2404
Cycle 458	2404
Cycle 459	2415
Cycle 460	2416
Cycle 461	2427
Cycle 462	2427
Cycle 463	2427
Cycle 464	2438
Cycle 465	2439
Cycle 466	2451
Start basecalling (301F - 150R)	2451
End basecalling (301F - 150R)	2458
Cycle 467	2459
Cycle 468	2463
Cycle 469	2463
Cycle 470	2464
Cycle 471	2475
Cycle 472	2475
Cycle 473	2487
Cycle 474	2487
Cycle 475	2488
Cycle 476	2499
Cycle 477	2499
Cycle 478	2511
Cycle 479	2512
Cycle 480	2512
Cycle 481	2524
Cycle 482	2525
Cycle 483	2537
Cycle 484	2537
Cycle 485	2537
Cycle 486	2548
Cycle 487	2549
Cycle 488	2561
Cycle 489	2561

Cycle 490	2561
Cycle 491	2574
Cycle 492	2574
Cycle 493	2587
Cycle 494	2587
Cycle 495	2588
Cycle 496	2600
Cycle 497	2600
Cycle 498	2612
Cycle 499	2613
Cycle 500	2613
Cycle 501	2625
Cycle 502	2625
Cycle 503	2639
Cycle 504	2639
Cycle 505	2639
Cycle 506	2651
Cycle 507	2652
Cycle 508	2664
Cycle 509	2664
Cycle 510	2664
Cycle 511	2679
Cycle 512	2679
Cycle 513	2697
Cycle 514	2698
Cycle 515	2698
Cycle 516	2710
Start basecalling (301F - 200R)	2710
Cycle 519	2711
End basecalling (301F - 200R)	2717
Cycle 520	2724
Cycle 521	2724
Cycle 522	2737
Cycle 523	2737
Cycle 524	2738
Cycle 525	2750
Cycle 526	2750
Cycle 527	2766
Cycle 528	2767
Cycle 529	2767
Cycle 530	2780
Cycle 531	2781
Cycle 532	2781
Cycle 533	2794
Cycle 534	2795
Cycle 535	2795
Cycle 536	2807
Cycle 537	2808
Cycle 538	2821
Cycle 539	2821
Cycle 540	2821
Cycle 541	2835
Cycle 542	2835
Cycle 543	2848
Cycle 544	2848
Cycle 545	2849

Cycle 546	2862
Cycle 547	2862
Cycle 548	2875
Cycle 549	2876
Cycle 550	2876
Cycle 551	2889
Cycle 552	2890
Cycle 553	2904
Cycle 554	2904
Cycle 555	2905
Cycle 556	2918
Cycle 557	2918
Cycle 558	2932
Cycle 559	2933
Cycle 560	2933
Cycle 561	2949
Cycle 562	2949
Cycle 563	2949
Cycle 564	2963
Cycle 565	2963
Cycle 566	2977
Start basecalling (301F - 250R)	2978
Cycle 568	2979
End basecalling (301F - 250R)	2985
Cycle 569	2992
Cycle 570	2993
Cycle 571	2993
Cycle 572	3008
Cycle 573	3008
Cycle 574	3023
Cycle 575	3023
Cycle 576	3023
Cycle 577	3038
Cycle 578	3038
Cycle 579	3038
Cycle 580	3052
Cycle 581	3052
Cycle 582	3067
Cycle 583	3067
Cycle 584	3068
Cycle 585	3082
Cycle 586	3083
Cycle 587	3097
Cycle 588	3097
Cycle 589	3097
Cycle 590	3112
Cycle 591	3112
Cycle 592	3126
Cycle 593	3127
Cycle 594	3127
Cycle 595	3141
Cycle 596	3142
Cycle 597	3142
Cycle 598	3157
Cycle 599	3158
Cycle 600	3172

Cycle 601	3172
Cycle 602	3172
Cycle 603	3189
Cycle 604	3189
Cycle 605	3205
Cycle 606	3205
Cycle 607	3205
Cycle 608	3220
Cycle 609	3220
Cycle 610	3220
Cycle 611	3236
Cycle 612	3236
Cycle 613	3266
Cycle 614	3266
Cycle 615	3266
Cycle 616	3279
Start basecalling (301F - 300R)	3279
End basecalling (301F - 300R)	3286
Basecalling done (on MiSEQ)	3332

Figures

Figure S1: Overview of predefined jobs in the 'data conversion queue' for the three technical replicates

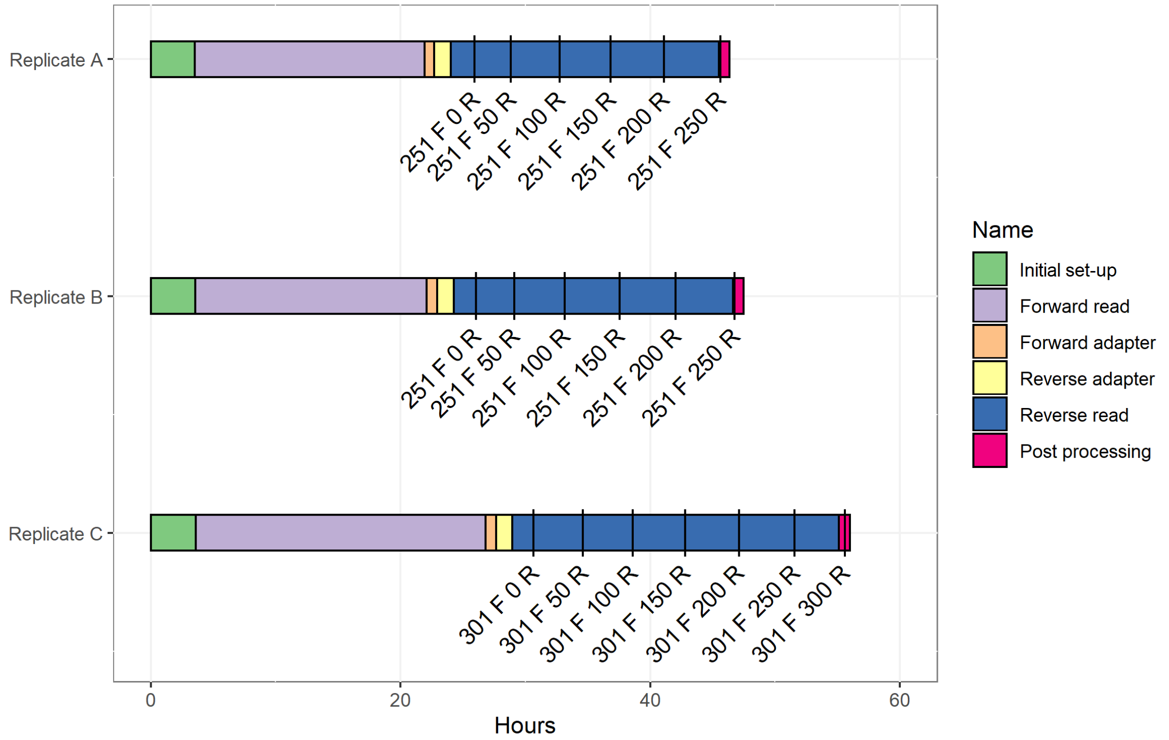

The technical replicate runs are presented on the y-axis, and their recorded times during sequencing are indicated on the x-axis. Replicates A and B were sequenced with read length 2x251 bp, replicate C with read length 2x301 bp. Colors on the bars indicate particular components of the sequencing process. The green color represents the initial startup after commencing the sequencing run during which no sequencing occurs. The purple color represents different sequencing cycles of the forward read, during which no analysis is possible because adaptors required for de-multiplexing still need to be sequenced. The orange and yellow colors represent sequencing cycles of the forward and reverse read adapters, respectively. The blue color represents sequencing cycles of the reverse read, during which vertical lines indicate points at which basecalling and data generation jobs were started for the corresponding pre-defined jobs. The red color represents post-processing of generated sequencing data by the MiSeq. Abbreviations: F, forward; R, reverse.

WGS data quality (symmetric read lengths)

Figure S2: Scenario 1 (No real-time analysis) - Effective coverage

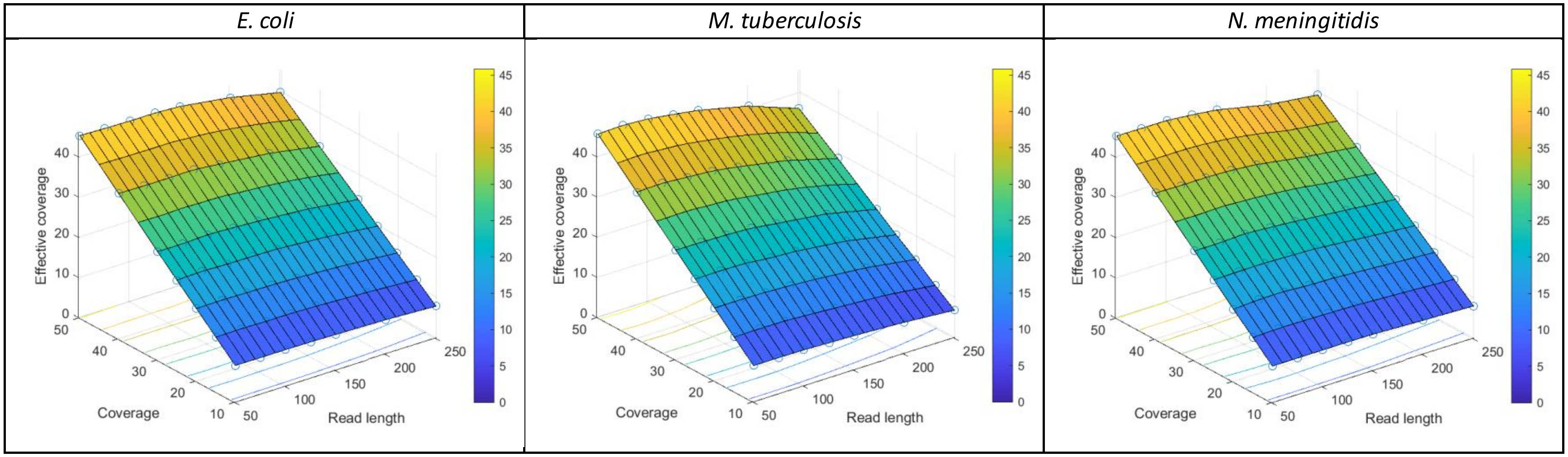

Figure S3: Scenario 1 (No real-time analysis) – N50

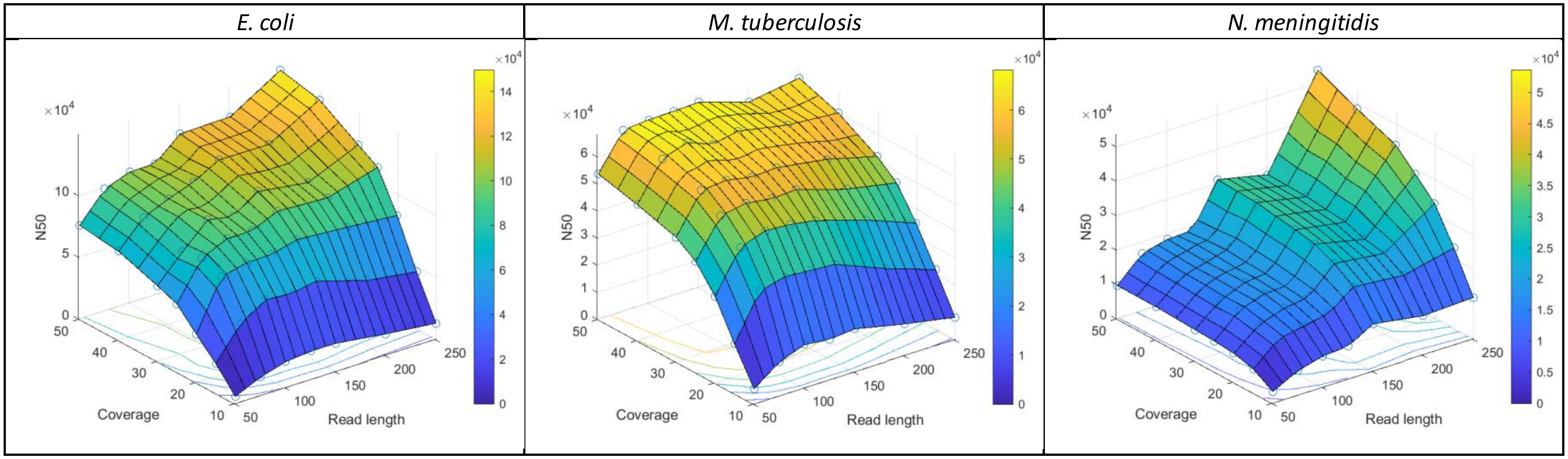

Figure S4: Scenario 1 (No real-time analysis) – Total assembly length

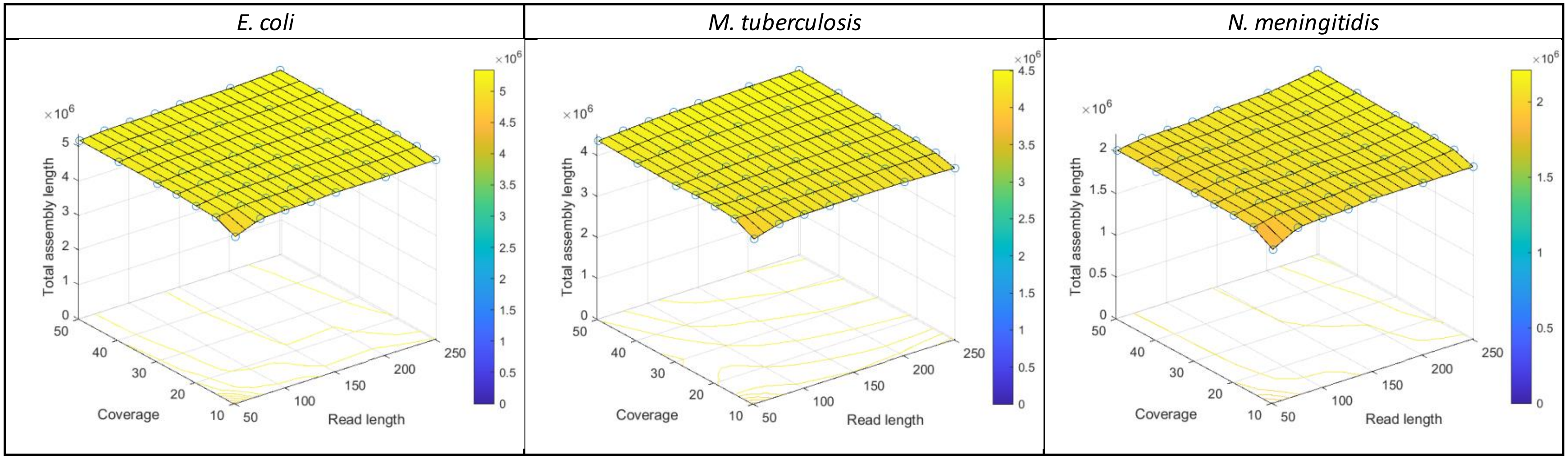

Figure S5: Scenario 2 (Real-time analysis) - Effective coverage

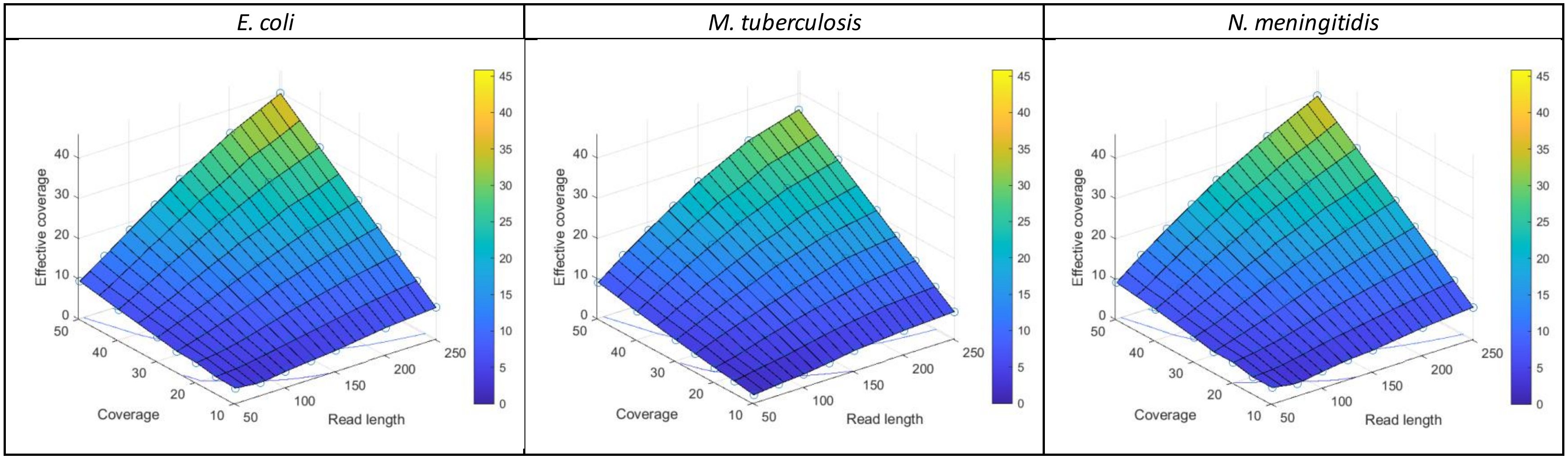

Figure S6: Scenario 2 (Real-time analysis) – N50

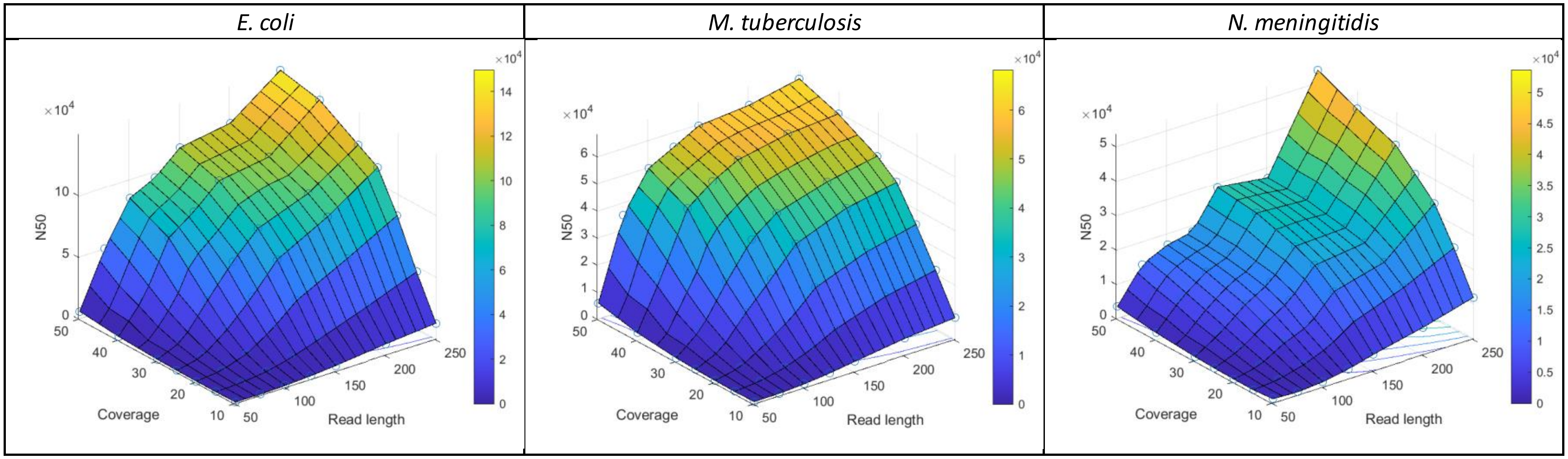

Figure S7: Scenario 2 (Real-time analysis) – Total assembly length

E. coli	*M. tuberculosis*	*N. meningitidis*
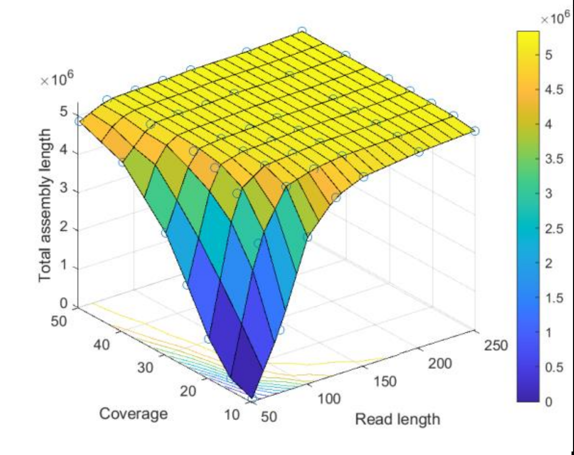	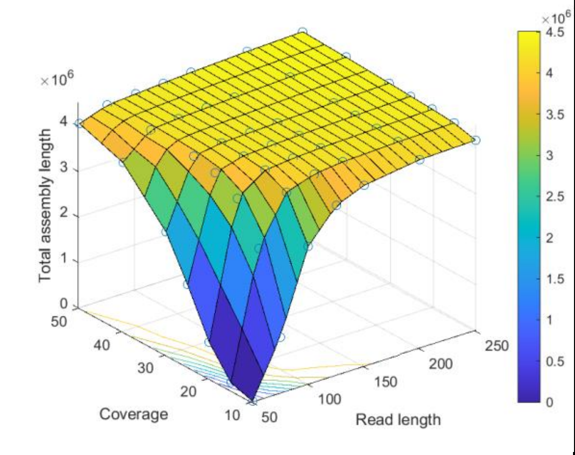	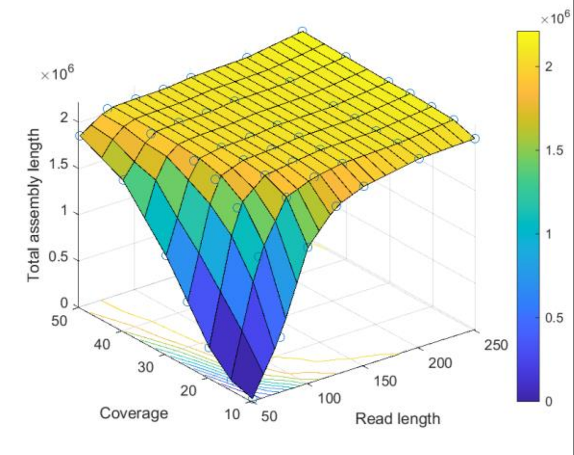

WGS data quality (asymmetric read lengths)

Figure S8: Scenario 1 (No real-time analysis) – Effective coverage – Asymmetric read-length combinations – *E. coli*

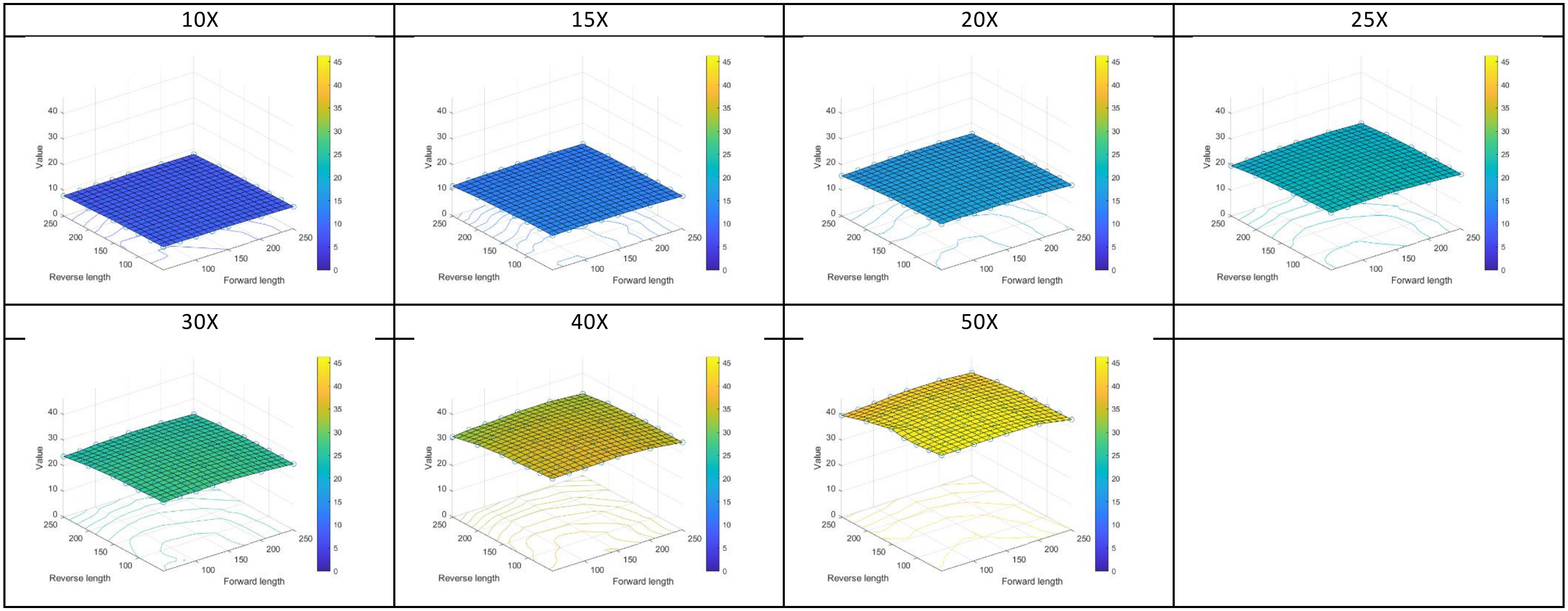

Figure S9: Scenario 1 (No real-time analysis) – Effective coverage – Asymmetric read-length combinations – *M. tuberculosis*

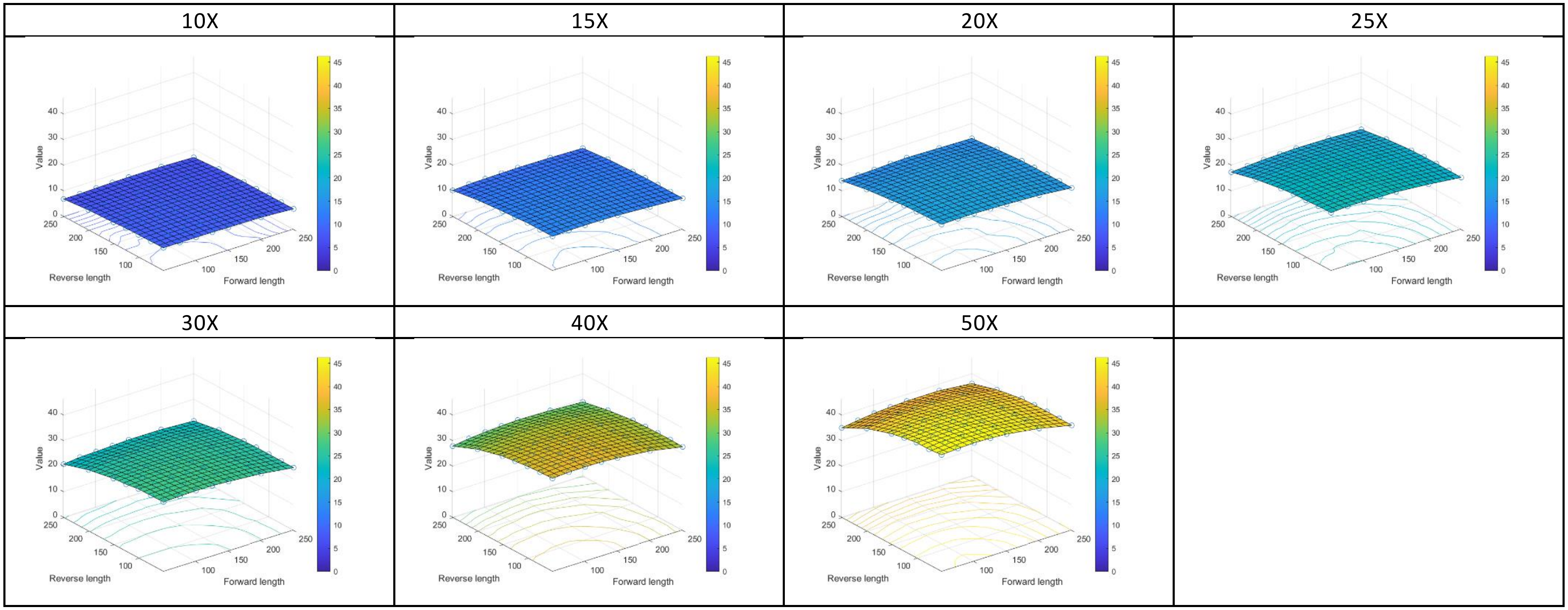

Figure S10: Scenario 1 (No real-time analysis) – Effective coverage – Asymmetric read-length combinations – *N. meningitidis*

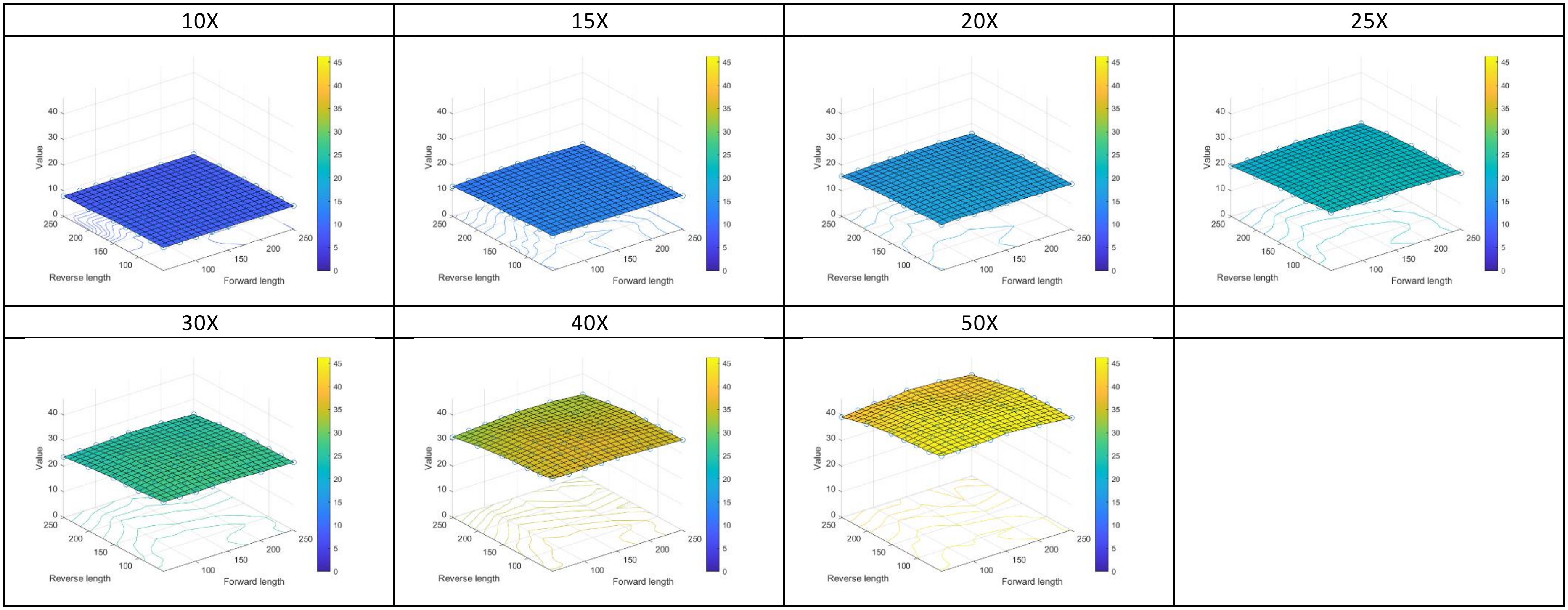

Figure S11: Scenario 1 (No real-time analysis) – N50 – Asymmetric read-length combinations – *E. coli*

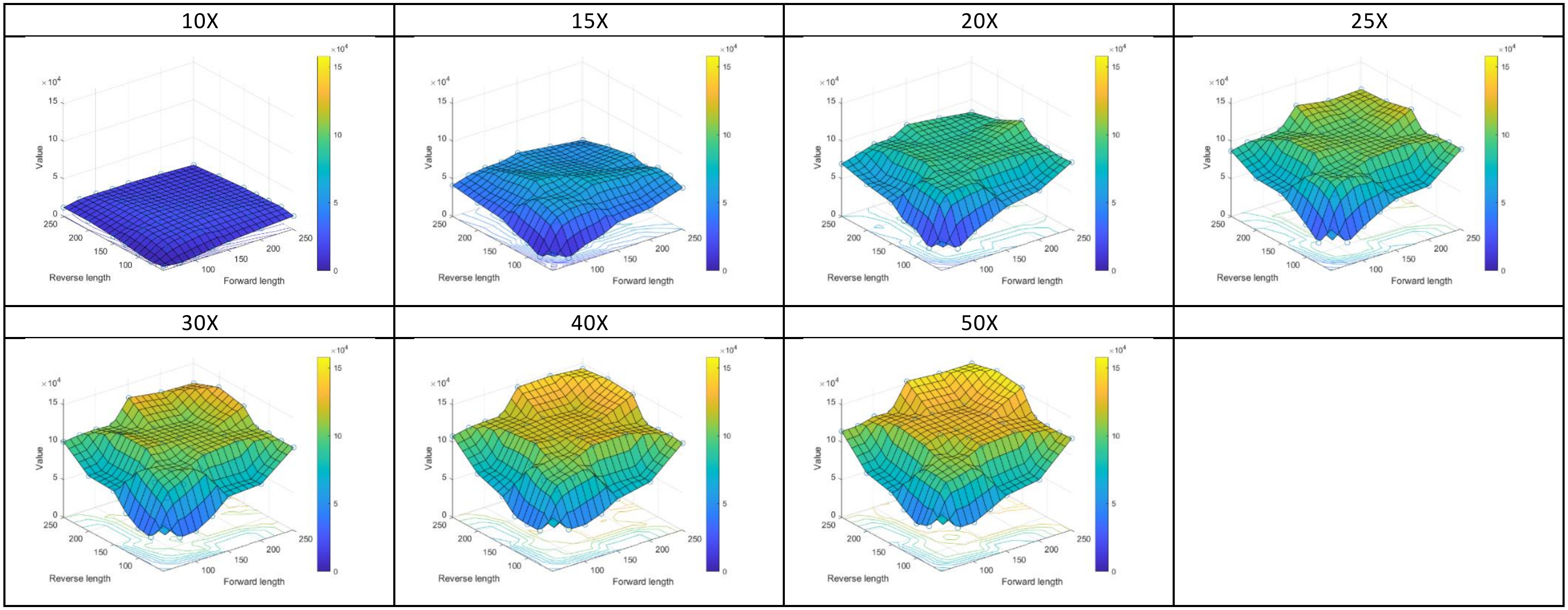

Figure S12: Scenario 1 (No real-time analysis) – N50 – Asymmetric read-length combinations – *M. tuberculosis*

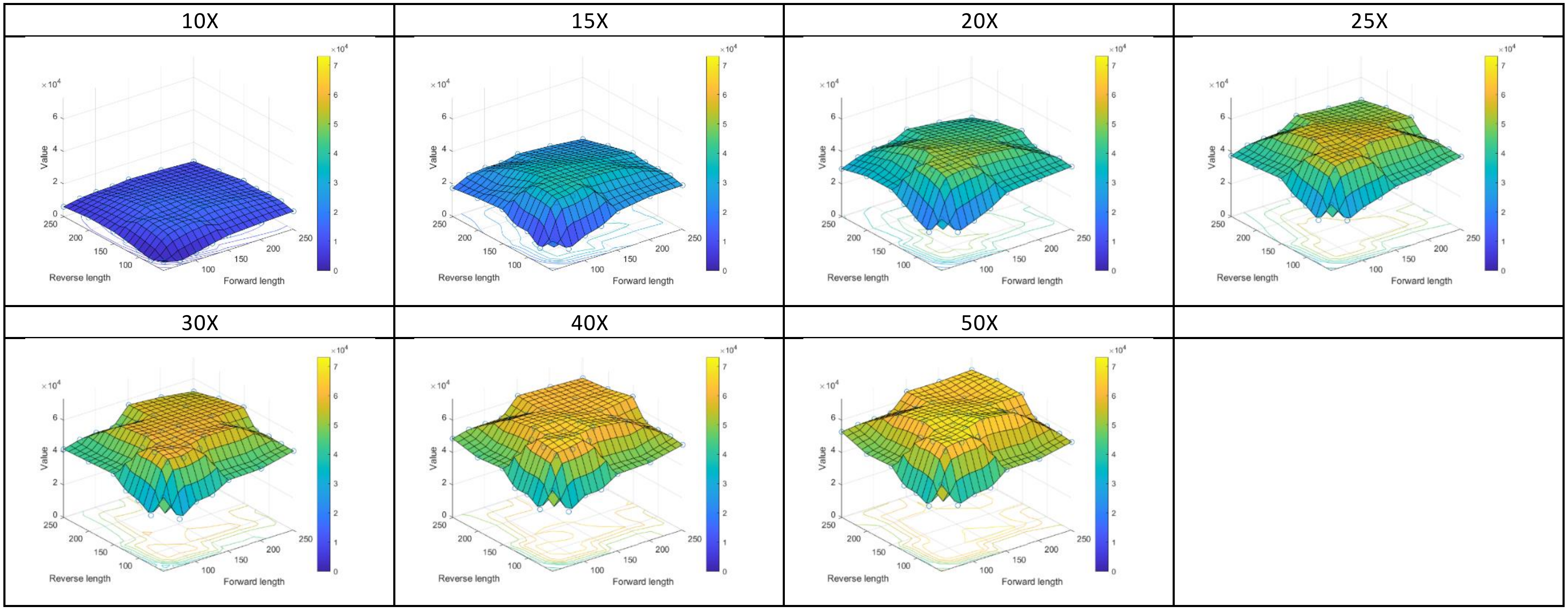

Figure S13: Scenario 1 (No real-time analysis) – N50 – Asymmetric read-length combinations – *N. meningitidis*

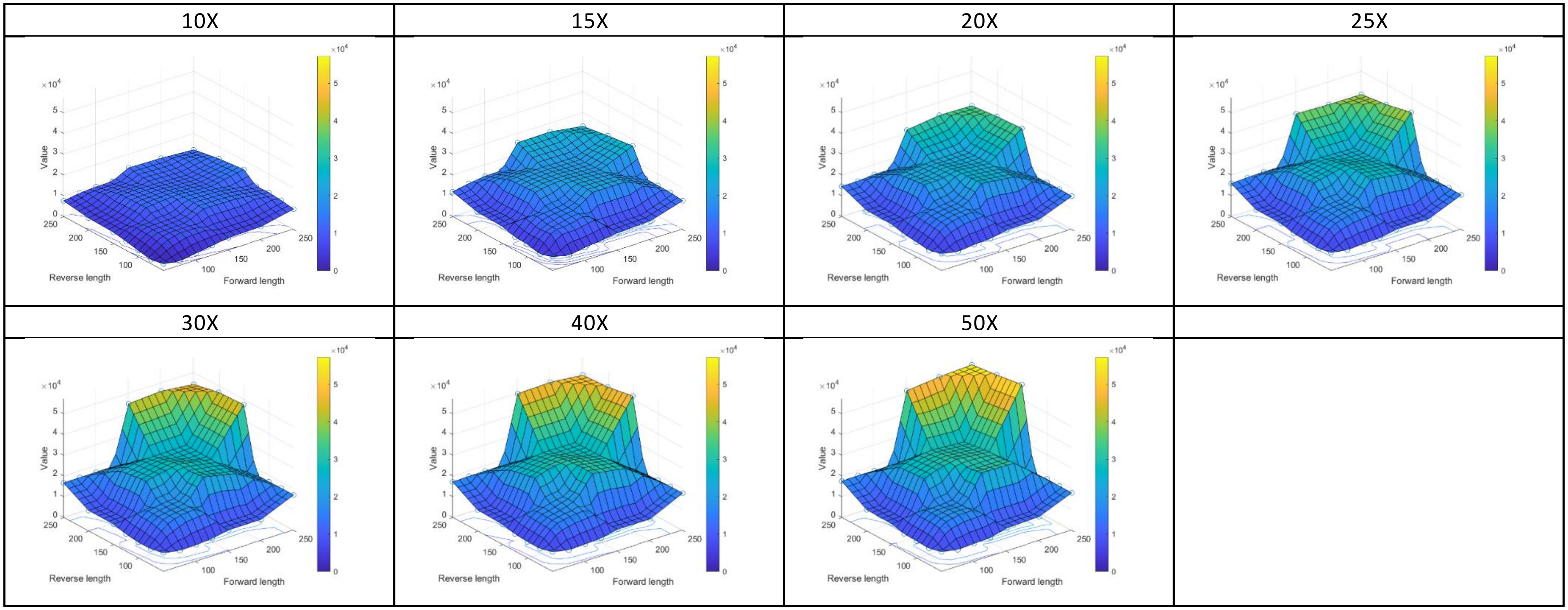

Figure S14: Scenario 1 (No real-time analysis) – Total assembly length – Asymmetric read-length combinations – *E. coli*

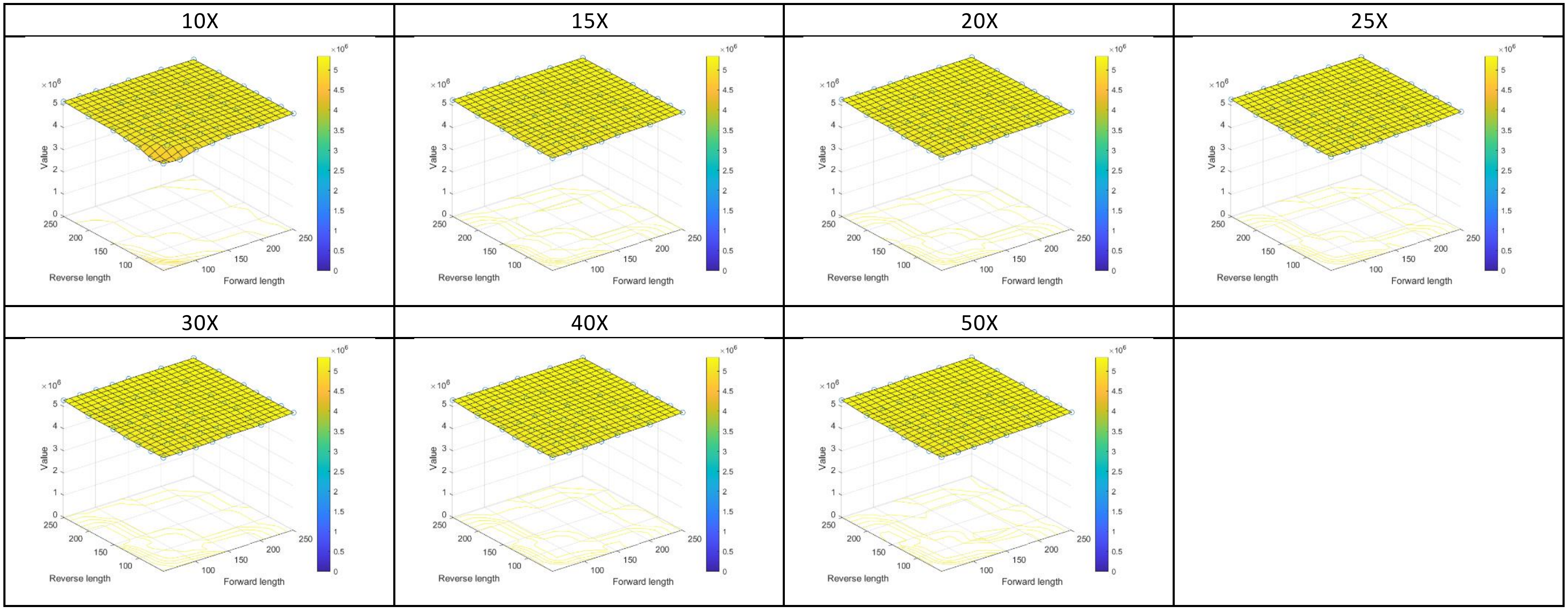

Figure S15: Scenario 1 (No real-time analysis) – Total assembly length – Asymmetric read-length combinations – *M. tuberculosis*

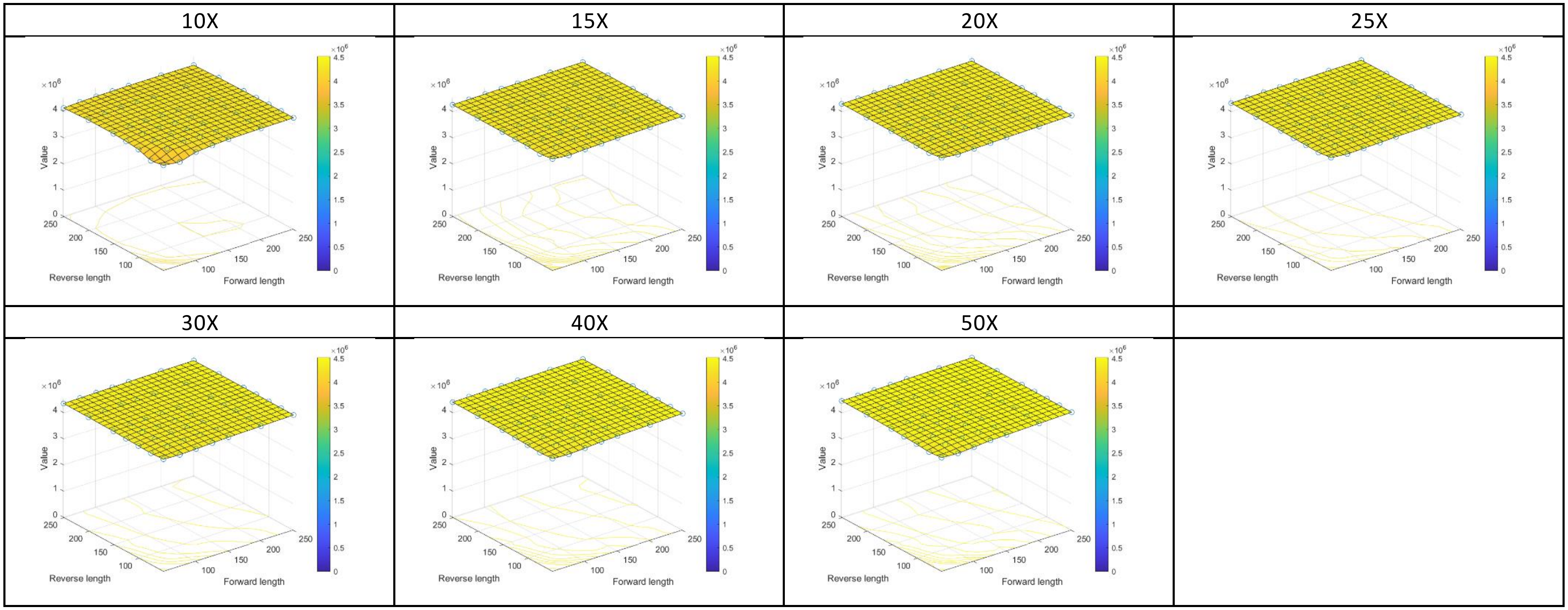

Figure S16: Scenario 1 (No real-time analysis) – Total assembly length – Asymmetric read-length combinations – *N. meningitidis*

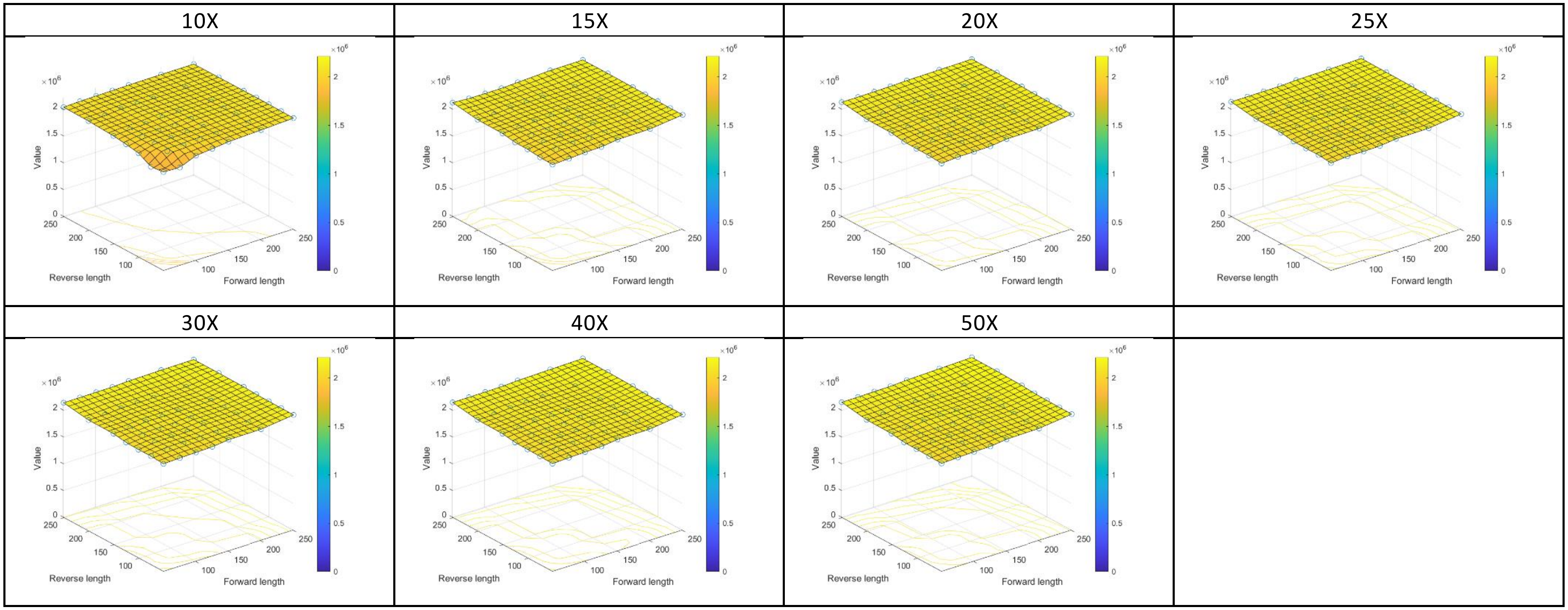

Figure S17: Scenario 2 (Real-time analysis) – Effective coverage – Asymmetric read-length combinations – *E. coli*

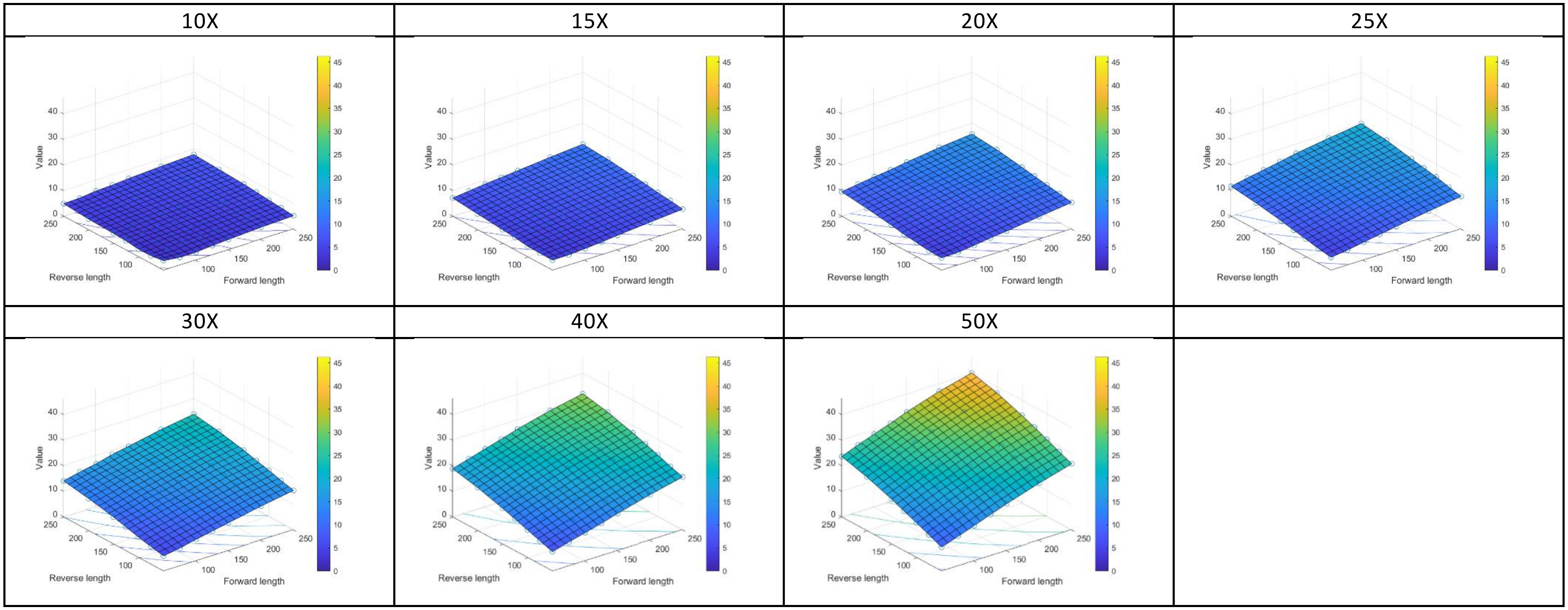

Figure S18: Scenario 2 (Real-time analysis) – Effective coverage – Asymmetric read-length combinations – *M. tuberculosis*

10X

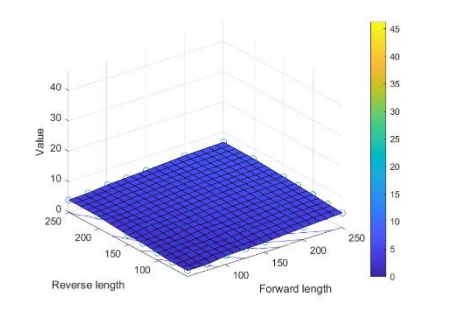

15X

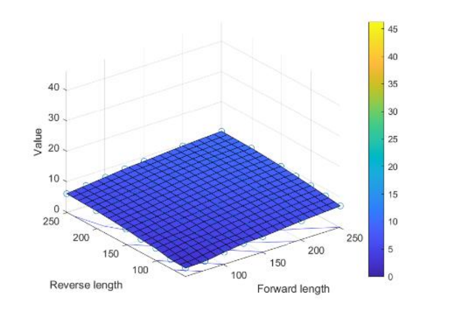

20X

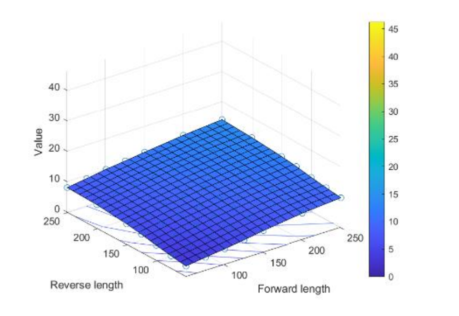

25X

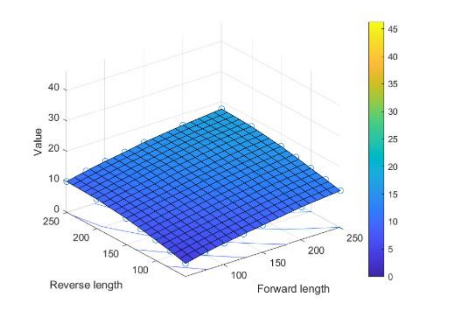

30X

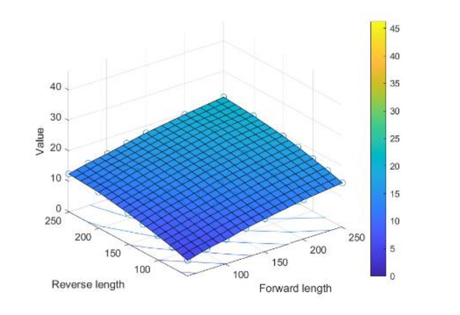

40X

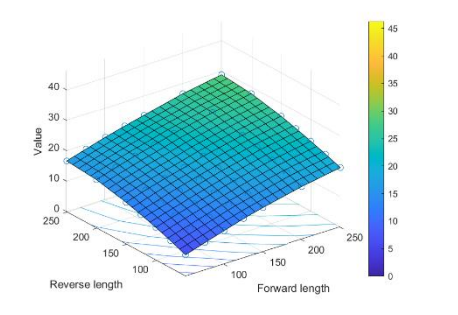

50X

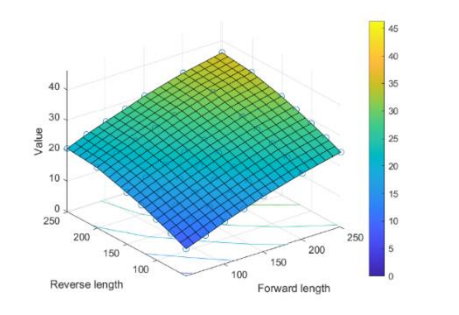

Figure S19: Scenario 2 (Real-time analysis) – Effective coverage – Asymmetric read-length combinations – *N. meningitidis*

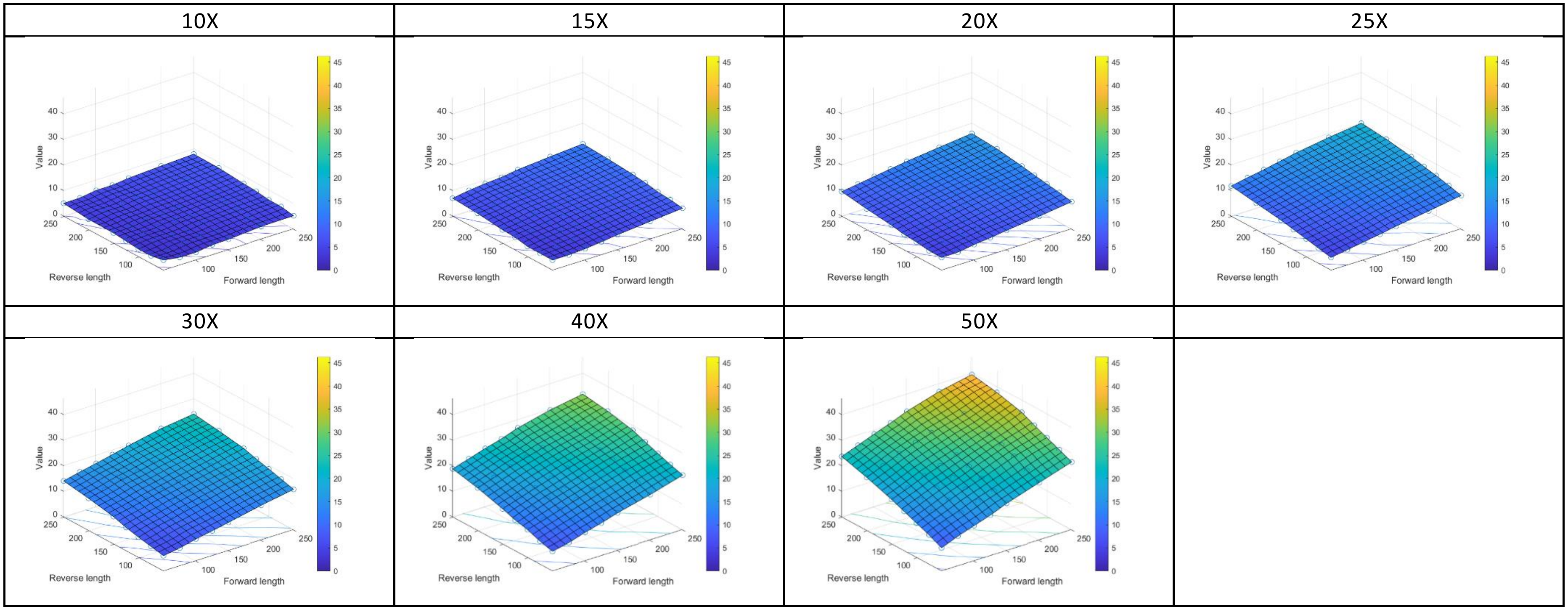

Figure S20: Scenario 2 (Real-time analysis) – N50 – Asymmetric read-length combinations – *E. coli*

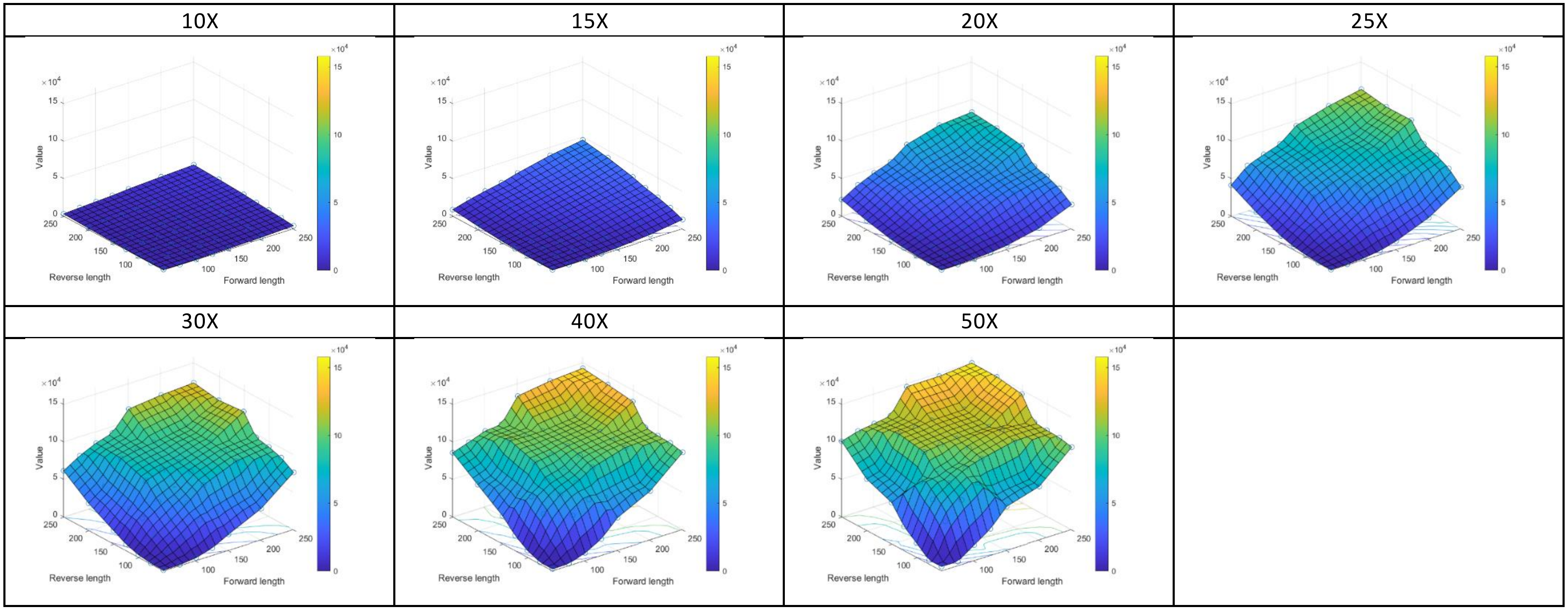

Figure S21: Scenario 2 (Real-time analysis) – N50 – Asymmetric read-length combinations – *M. tuberculosis*

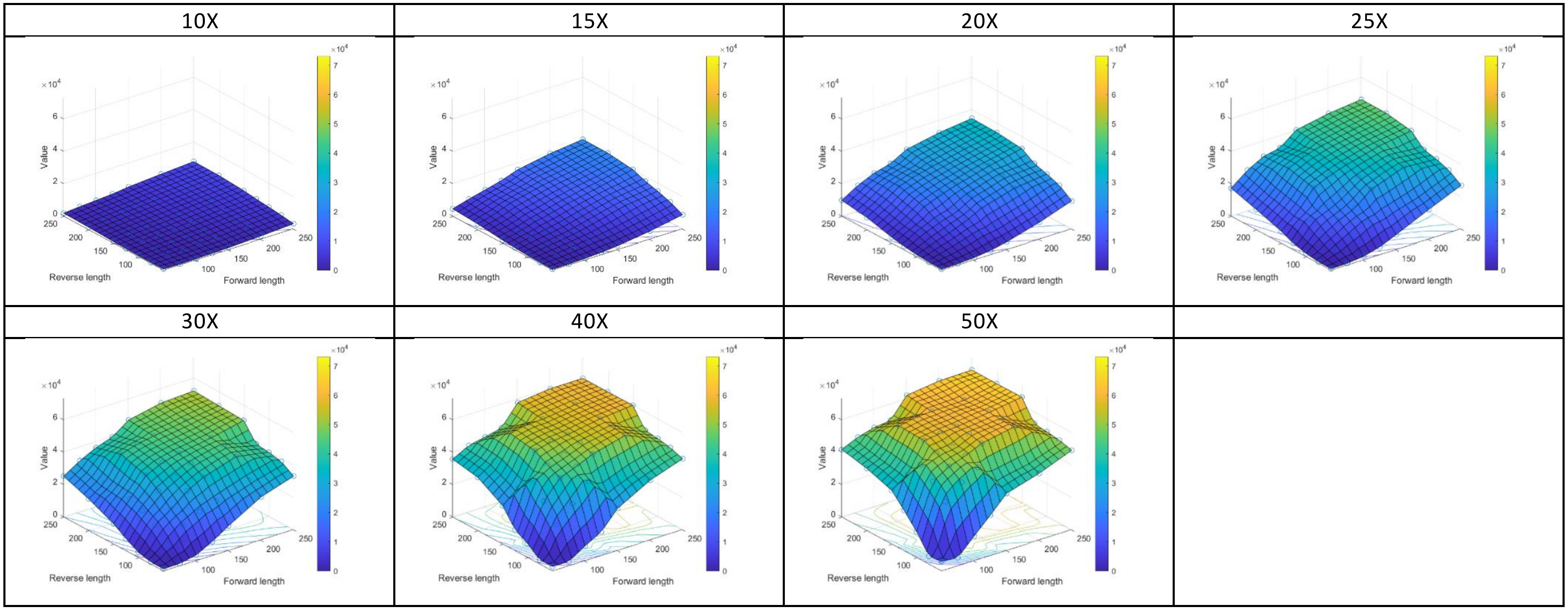

Figure S22: Scenario 2 (Real-time analysis) – N50 – Asymmetric read-length combinations – *N. meningitidis*

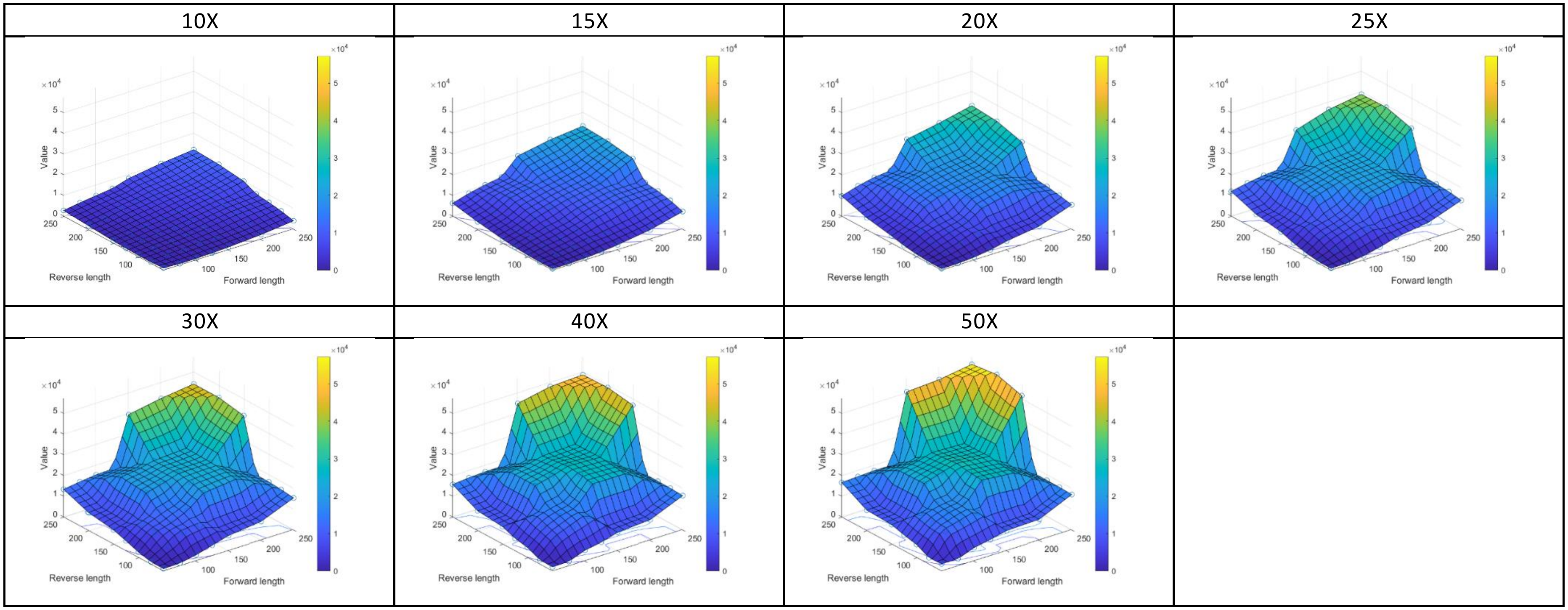

Figure S23: Scenario 2 (Real-time analysis) – Total assembly length – Asymmetric read-length combinations – *E. coli*

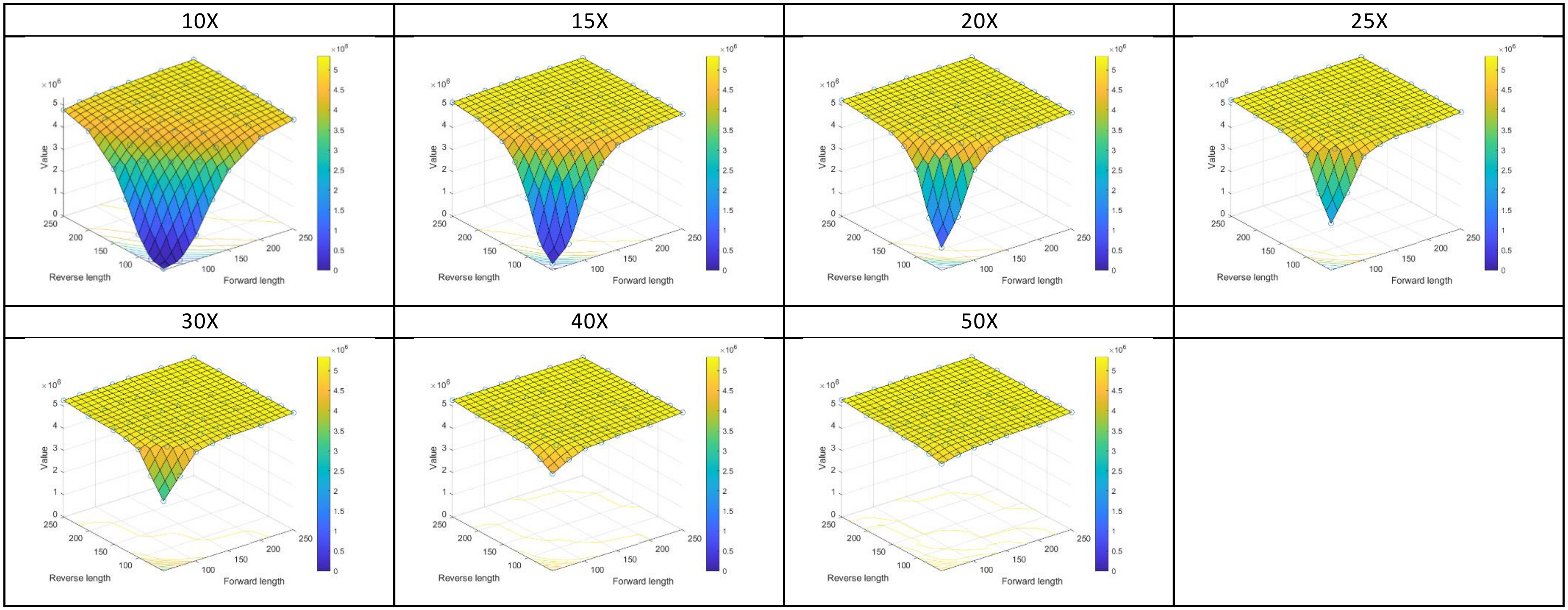

Figure S24: Scenario 2 (Real-time analysis) – Total assembly length – Asymmetric read-length combinations – *M. tuberculosis*

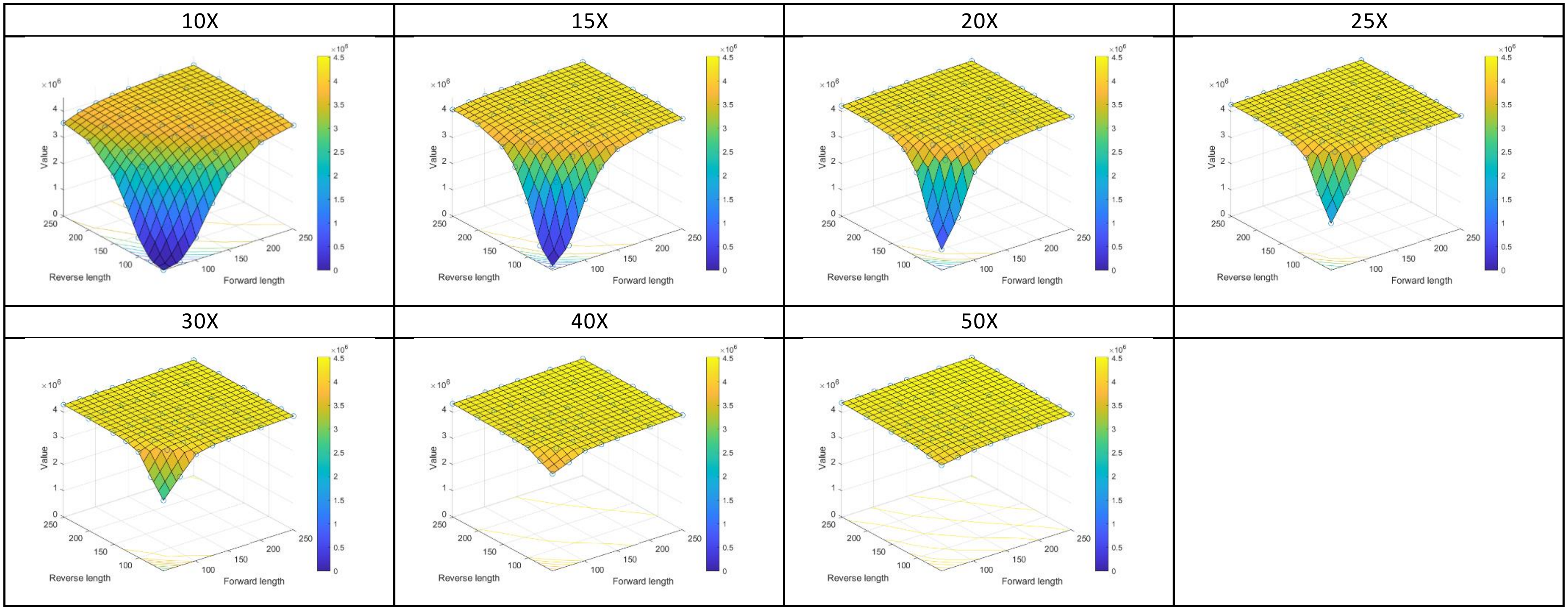

Figure S25: Scenario 2 (Real-time analysis) – Total assembly length – Asymmetric read-length combinations – *N. meningitidis*

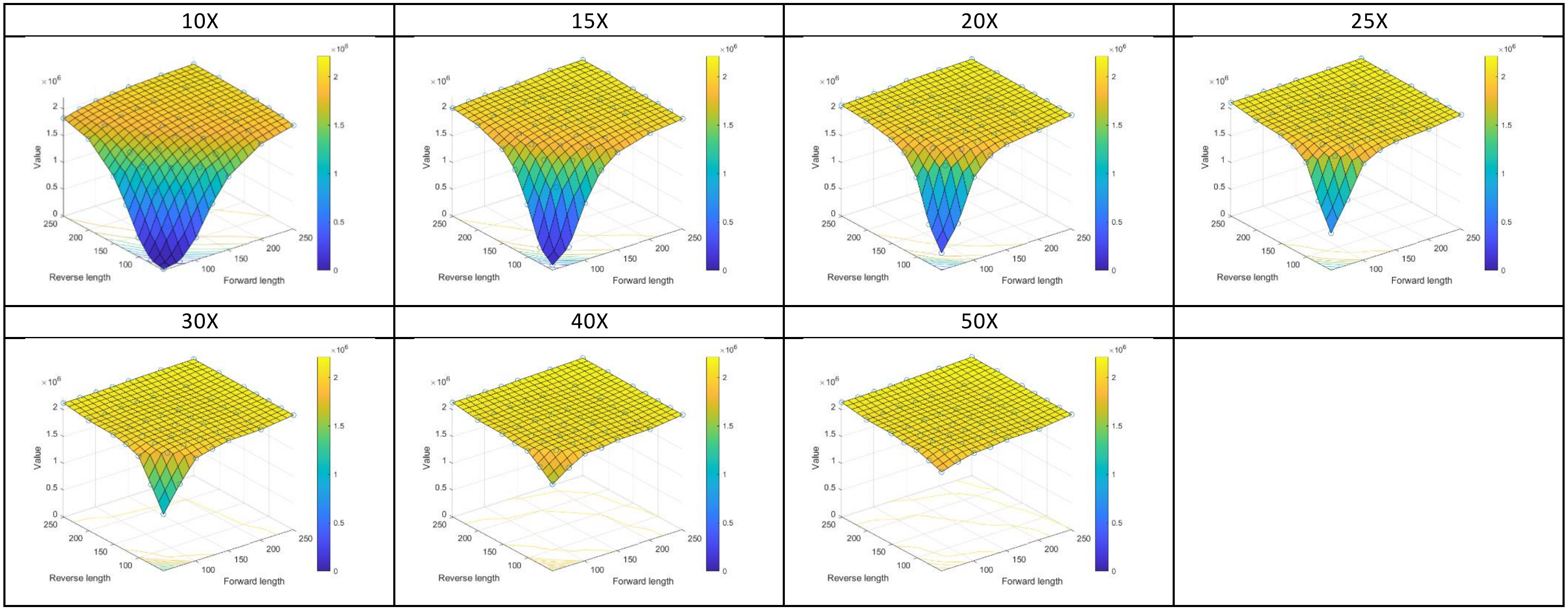

Assay performance (symmetric read lengths)

Figure S26: Scenario 1 (No real-time analysis) – 16S rRNA species confirmation

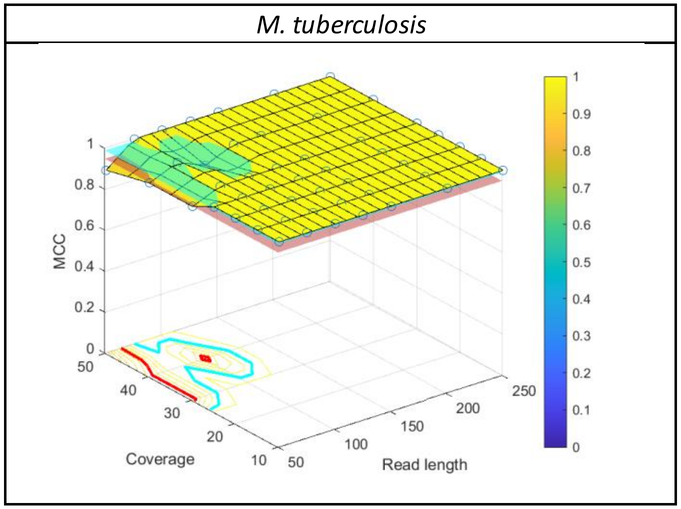

Figure S27: Scenario 1 (No real-time analysis) – Gene detection: AMR genes

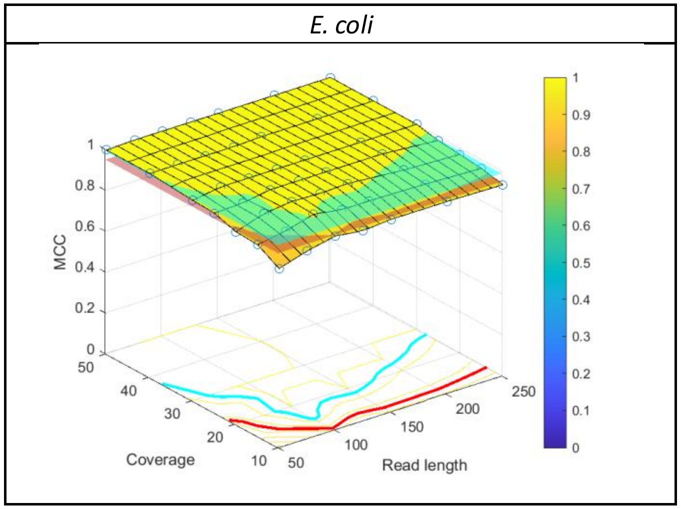

Figure S28: Scenario 1 (No real-time analysis) – Gene detection: Virulence genes

E. coli	*N. meningitidis*
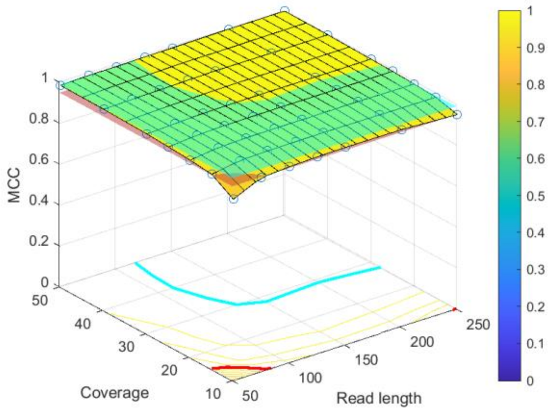	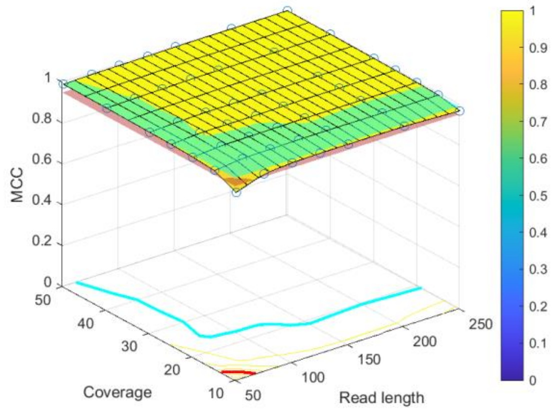

Figure S29: Scenario 1 (No real-time analysis) – PointFinder

E. coli	*M. tuberculosis*
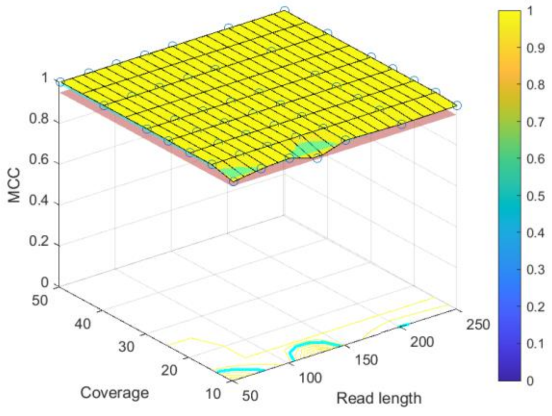	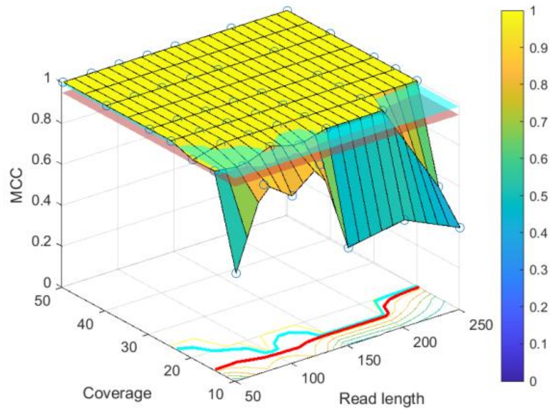

Figure S30: Scenario 1 (No real-time analysis) – SNP-based antimicrobial resistance detection

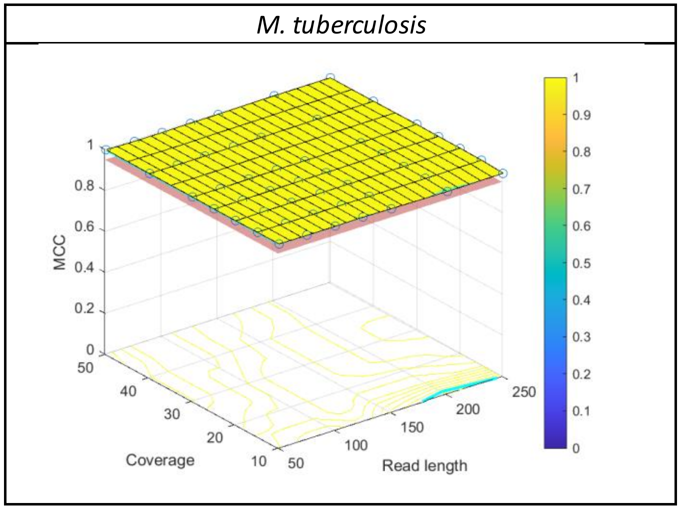

Figure S31: Scenario 1 (No real-time analysis) – Sequence typing

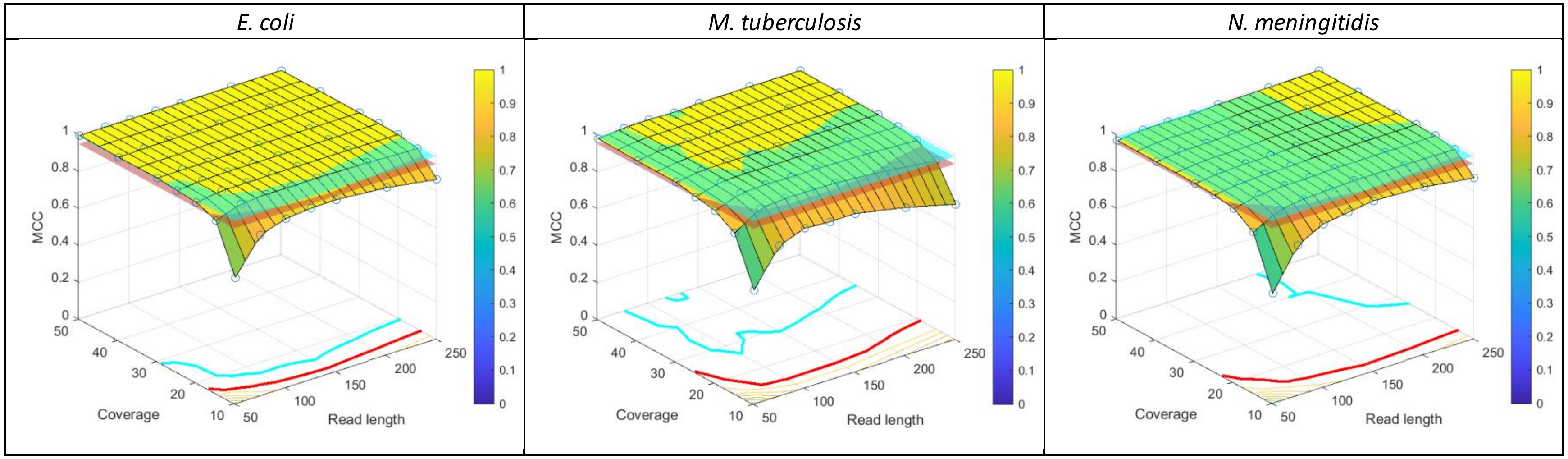

Figure S32: Scenario 1 (No real-time analysis) – Serotype determination: *E. coli*

E. coli

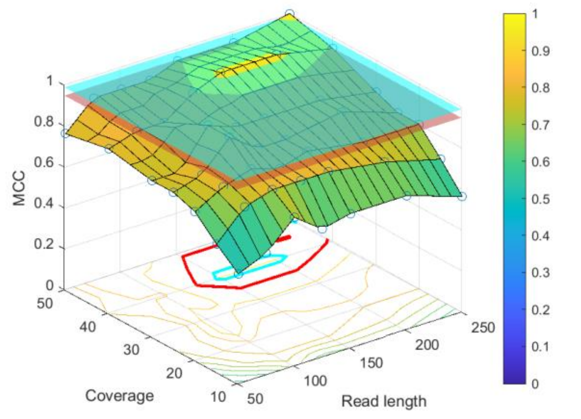

Figure S33: Scenario 1 (No real-time analysis) – Serotype determination: *N. meningitidis*

N. meningitidis

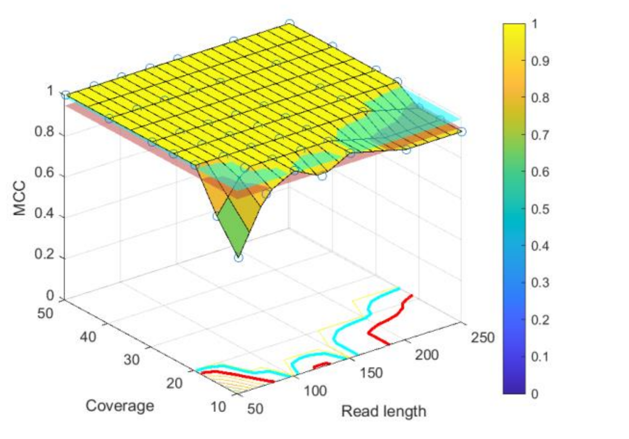

Figure S34: Scenario 2 (Real-time analysis) – 16S rRNA species confirmation

M. tuberculosis

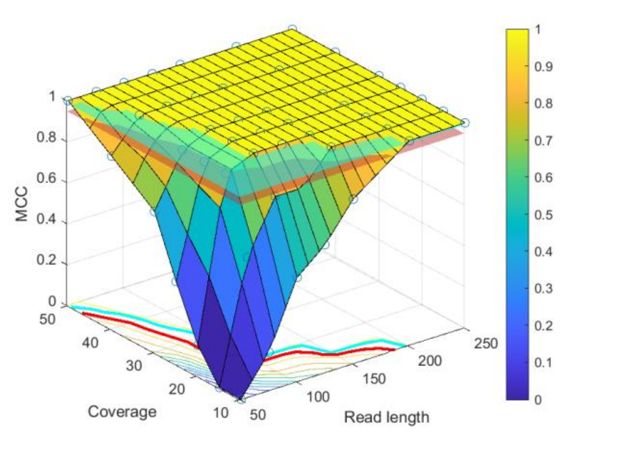

382 **Figure S35: Scenario 2 (Real-time analysis) – Gene detection: AMR genes**

383

E. coli

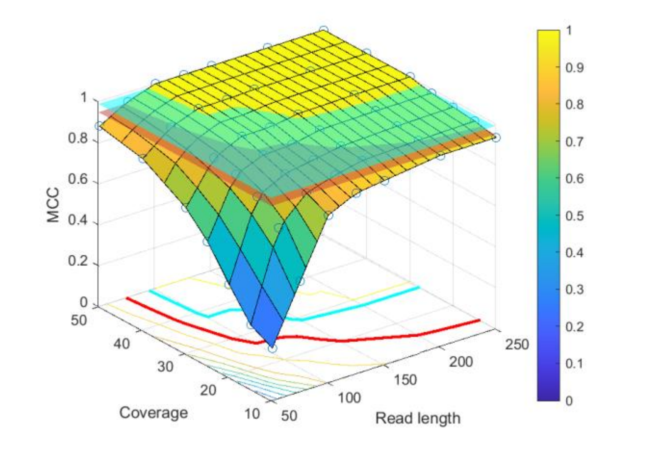

384

385

Figure S36: Scenario 2 (Real-time analysis) – Gene detection: Virulence genes

E. coli	*N. meningitidis*
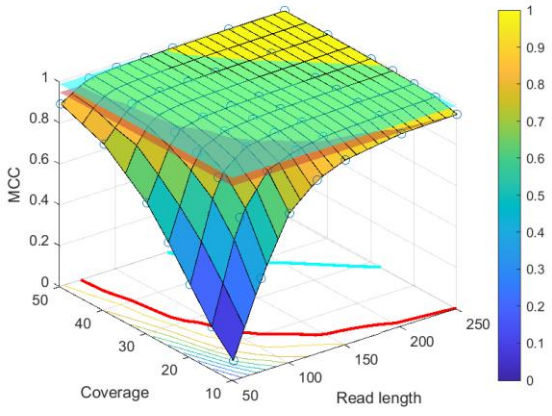	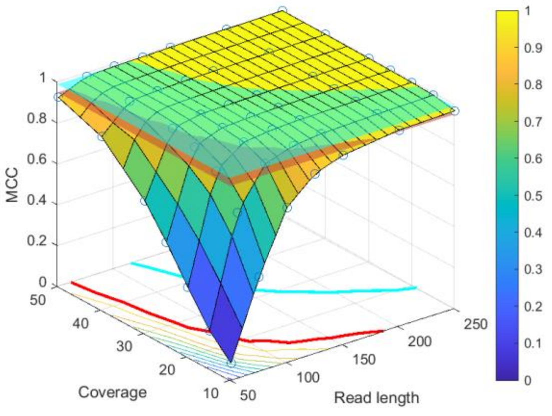

Figure S37: Scenario 2 (Real-time analysis) – PointFinder

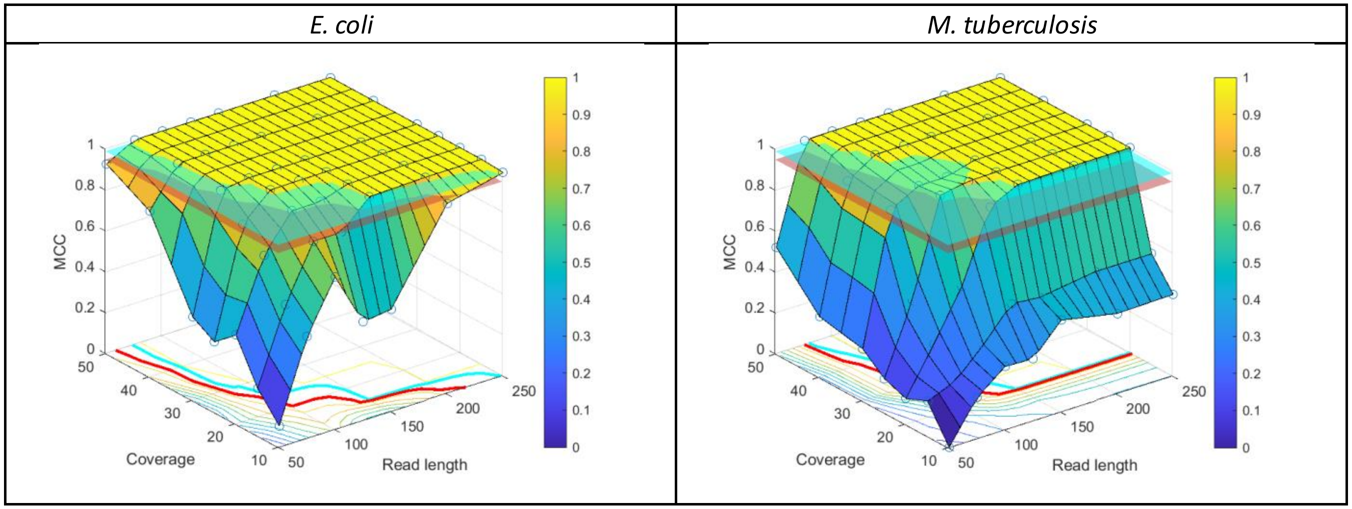

Figure S38: Scenario 2 (Real-time analysis) – SNP-based antimicrobial resistance detection

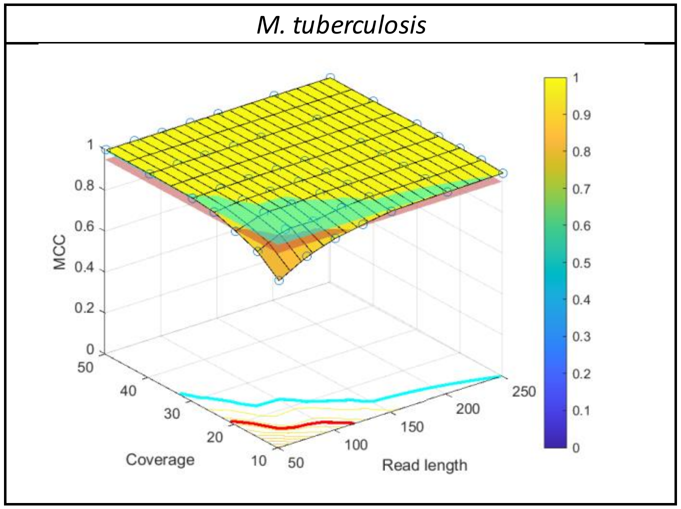

Figure S39: Scenario 2 (Real-time analysis) – Sequence typing

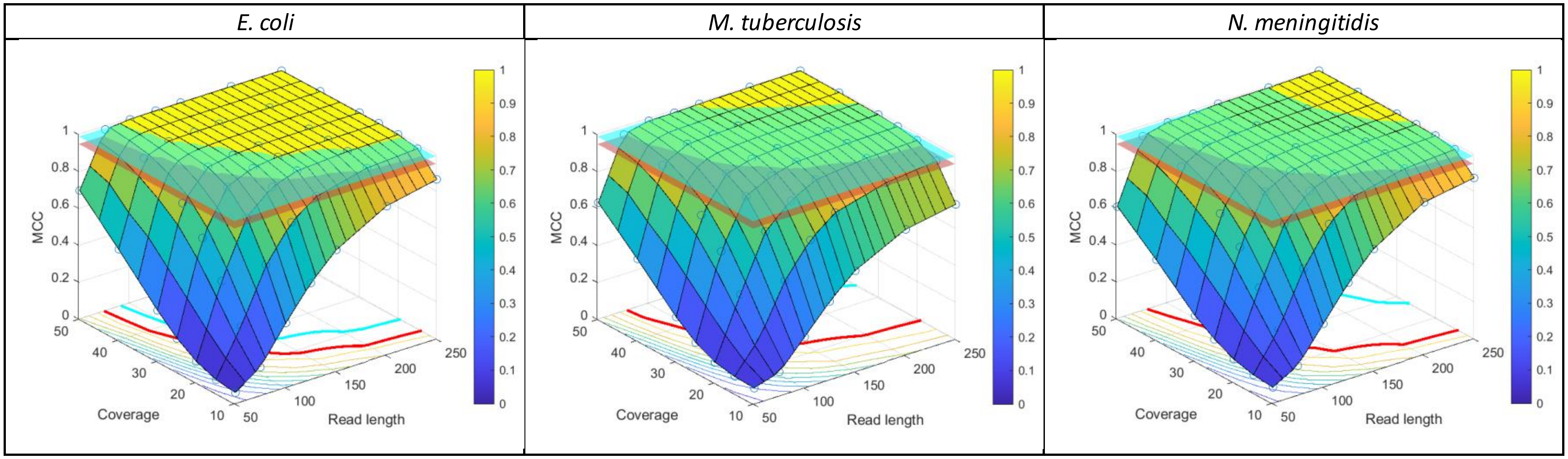

Figure S40: Scenario 2 (Real-time analysis) – Serotype determination: *E. coli*

E. coli

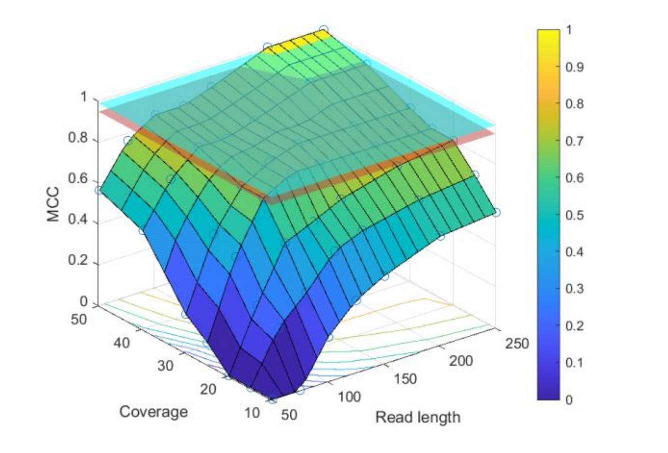

Figure S41: Scenario 2 (Real-time analysis) – Serotype determination: *N. meningitidis*

N. meningitidis

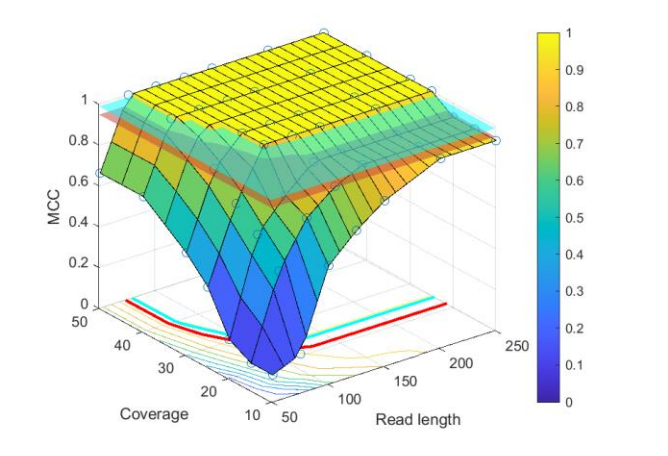

Assay performance (asymmetric read lengths)

Figure S42: Scenario 1 (No real-time analysis) – 16S rRNA species confirmation – Asymmetric read-length combinations – *M. tuberculosis*

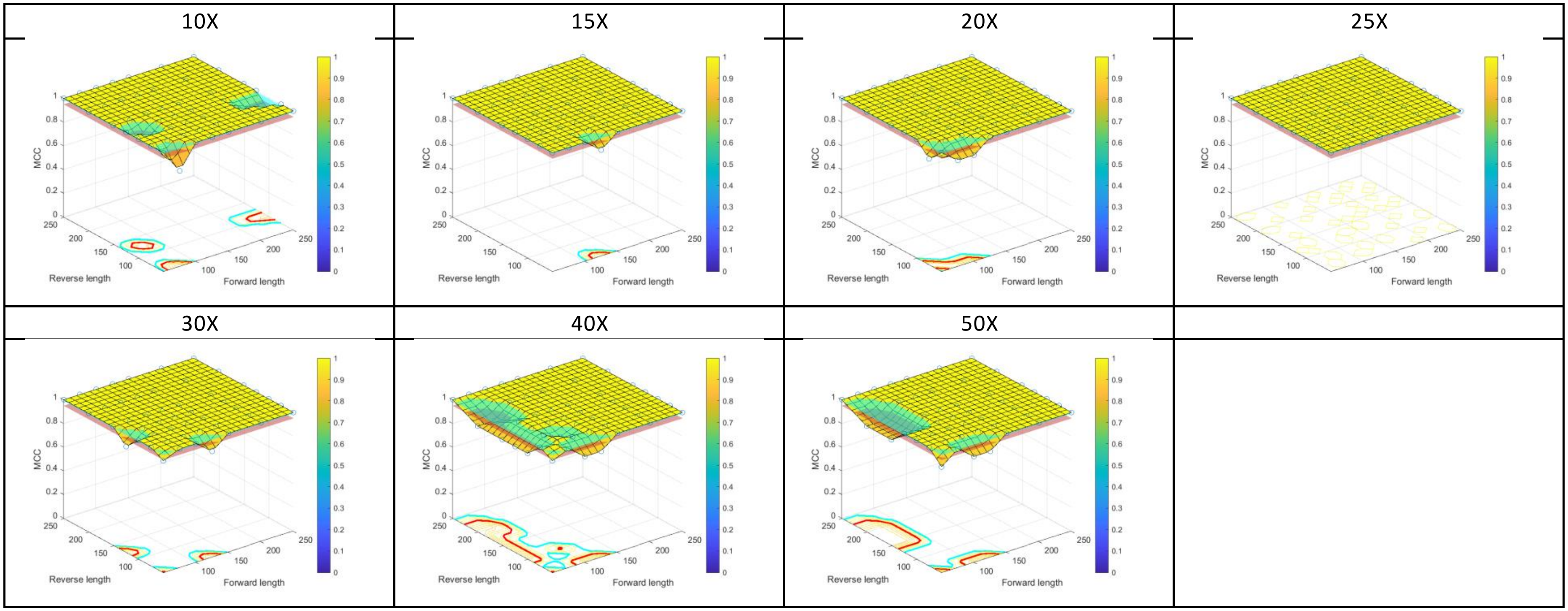

Figure S43: Scenario 1 (No real-time analysis) – Gene detection: AMR genes – Asymmetric read-length combinations – *E. coli*

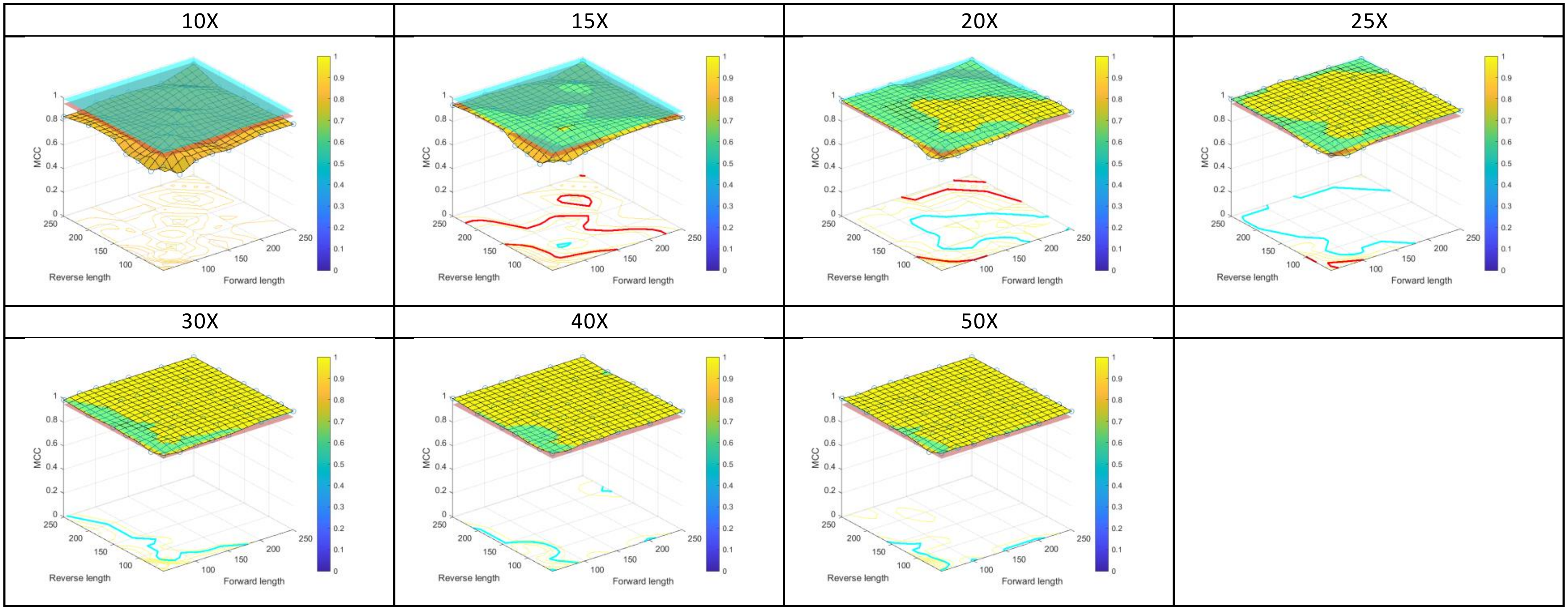

Figure S44: Scenario 1 (No real-time analysis) – Gene detection: Virulence genes – Asymmetric read-length combinations – *E. coli*

10X	15X	20X	25X
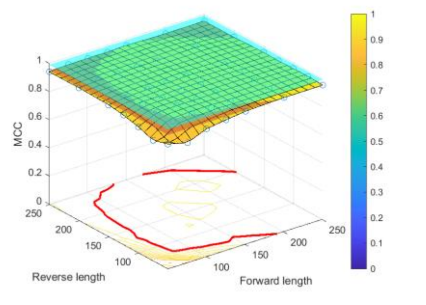	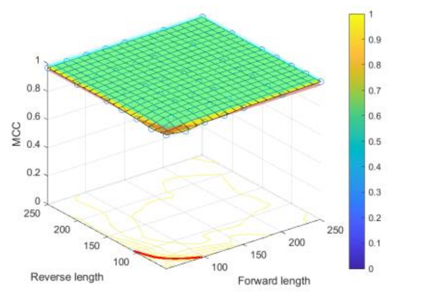	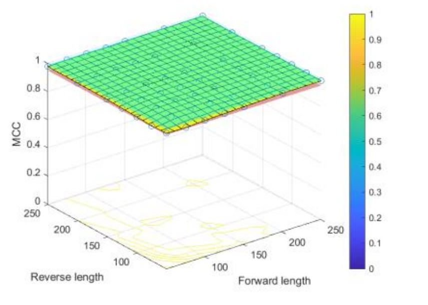	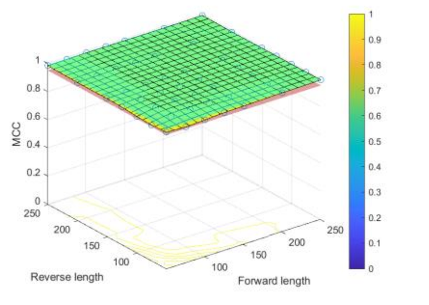
**30X**	**40X**	**50X**	
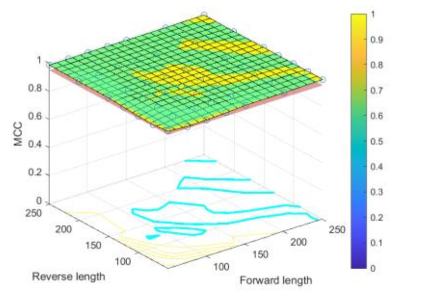	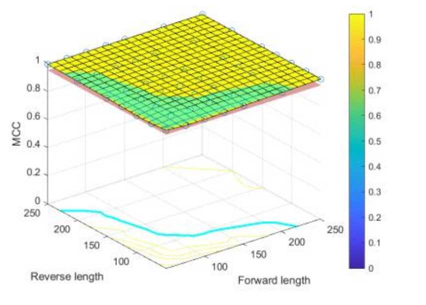	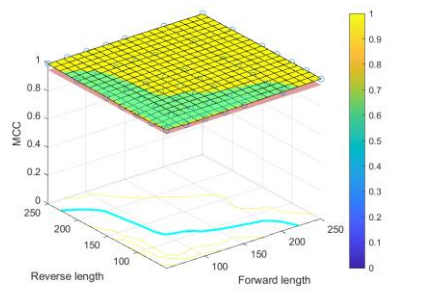	

Figure S45: Scenario 1 (No real-time analysis) – Gene detection: Virulence genes – Asymmetric read-length combinations – *N. meningitidis*

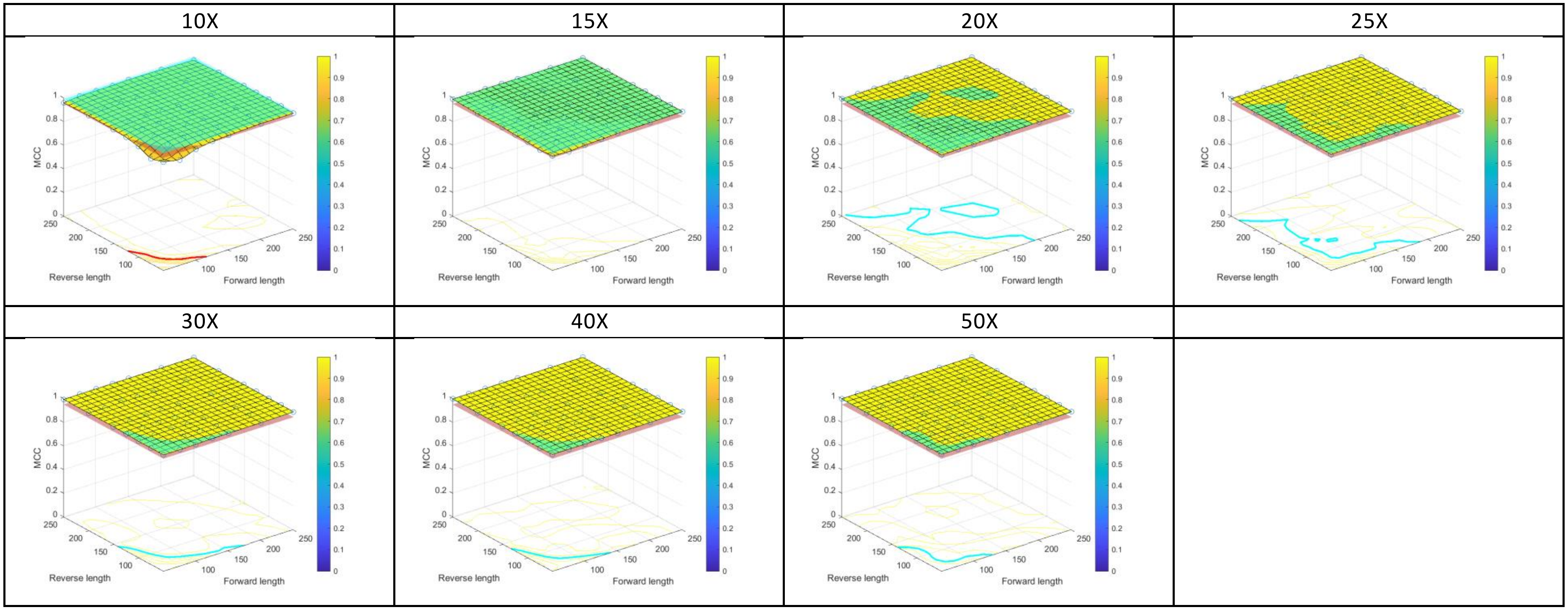

Figure S46: Scenario 1 (No real-time analysis) – PointFinder – Asymmetric read-length combinations – *E. coli*

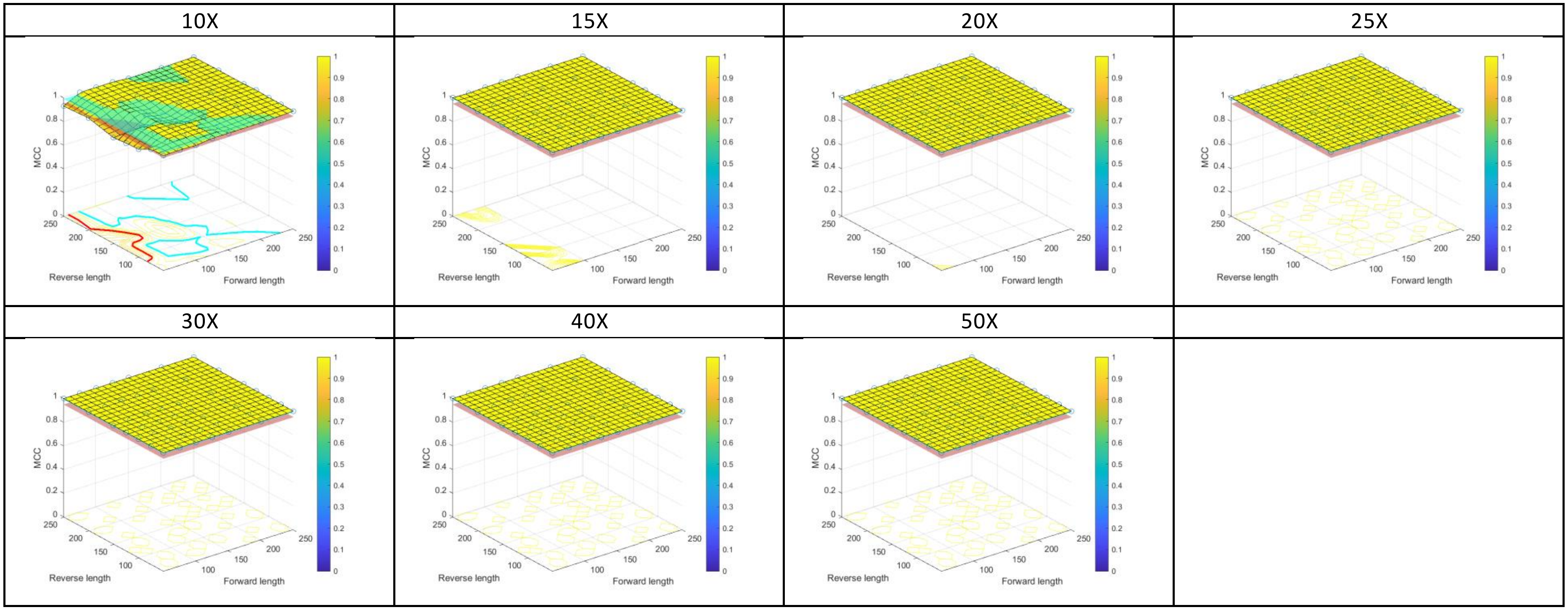

Figure S47: Scenario 1 (No real-time analysis) – PointFinder – Asymmetric read-length combinations – *M. tuberculosis*

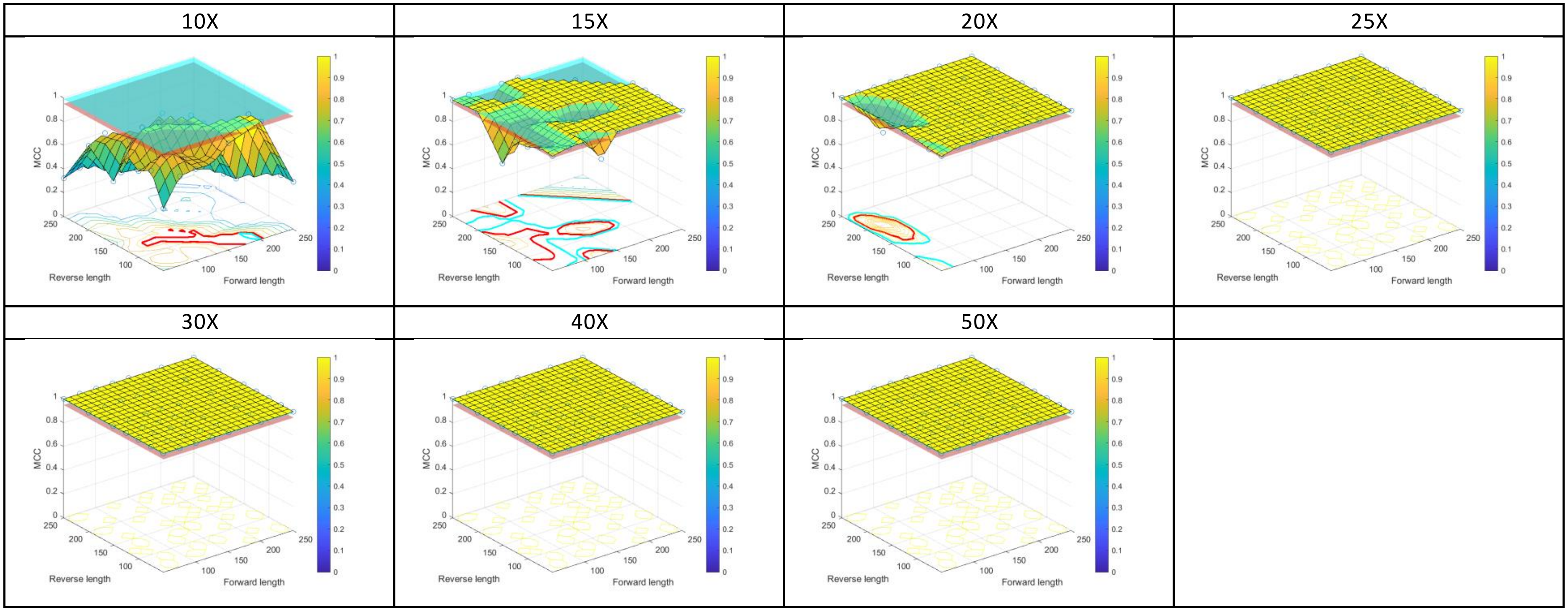

Figure S48: Scenario 1 (No real-time analysis) – SNP-based antimicrobial resistance detection – Asymmetric read-length combinations – *M. tuberculosis*

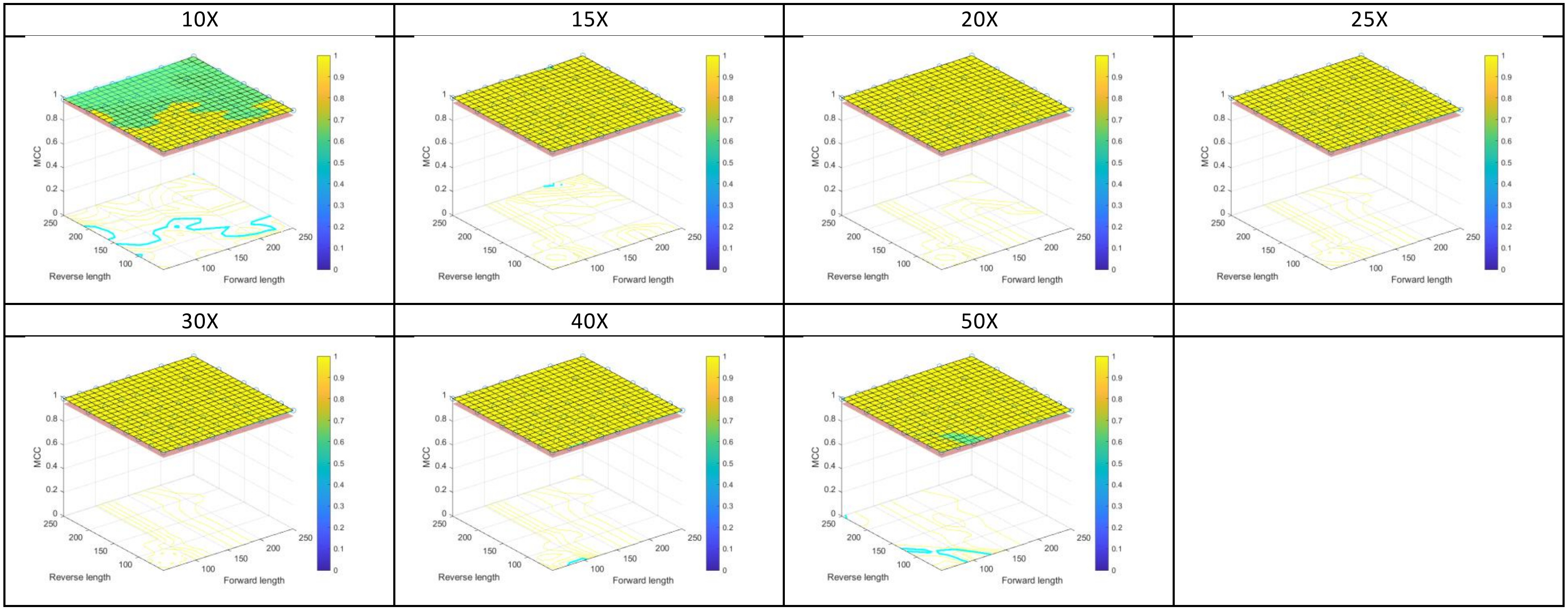

Figure S49: Scenario 1 (No real-time analysis) – Sequence typing – Asymmetric read-length combinations – *E. coli*

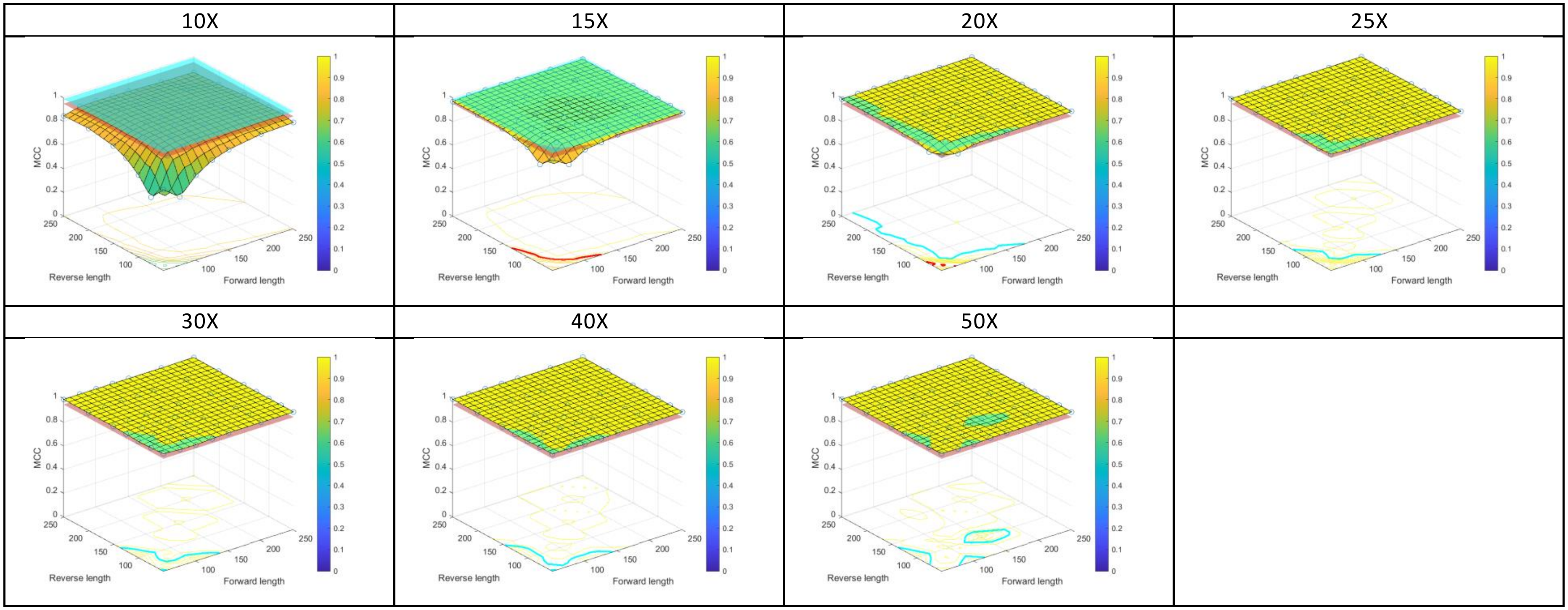

Figure S50: Scenario 1 (No real-time analysis) – Sequence typing – Asymmetric read-length combinations – *M. tuberculosis*

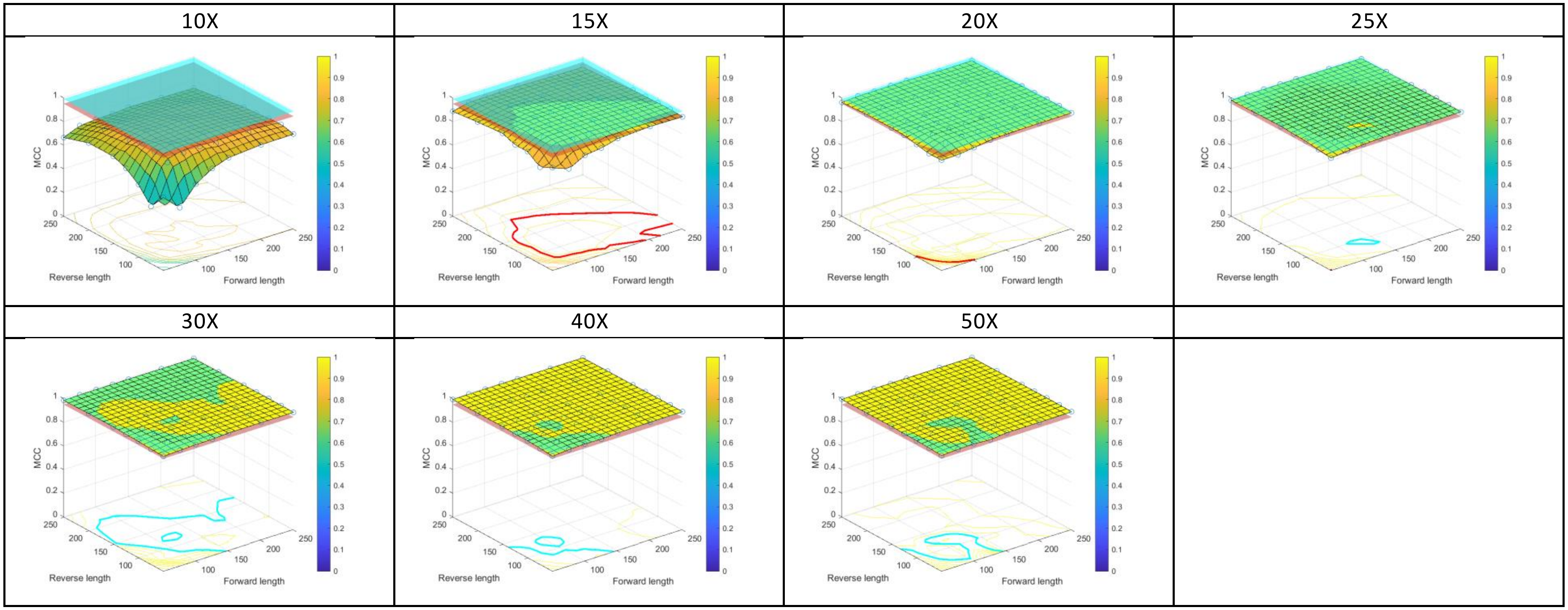

Figure S51: Scenario 1 (No real-time analysis) – Sequence typing – Asymmetric read-length combinations – *N. meningitidis*

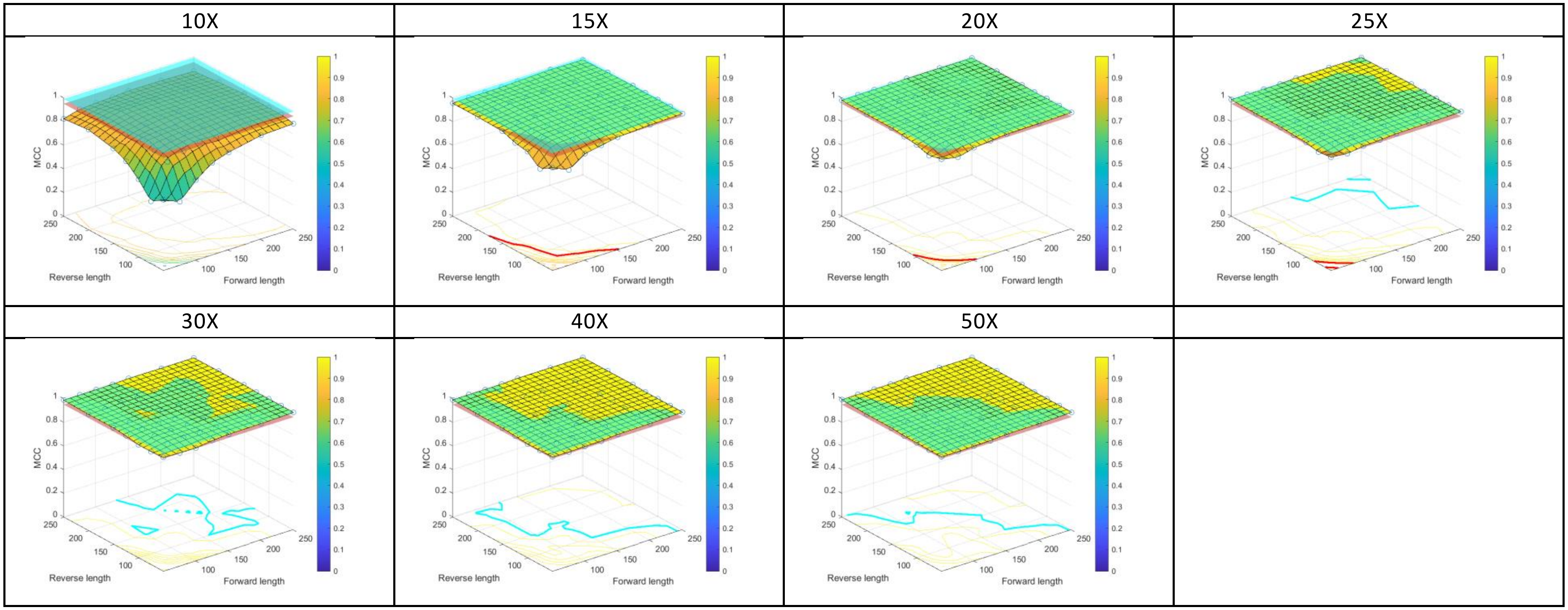

Figure S52: Scenario 1 (No real-time analysis) – Serotype determination – Asymmetric read-length combinations – *E. coli*

10X	15X	20X	25X
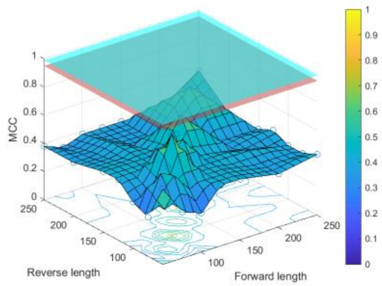	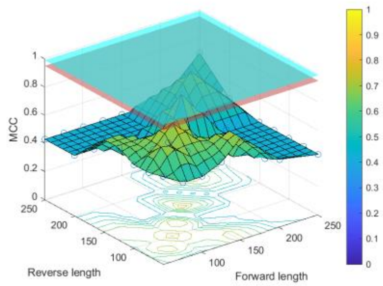	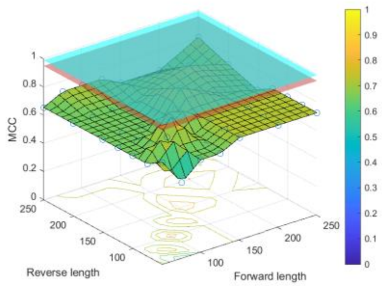	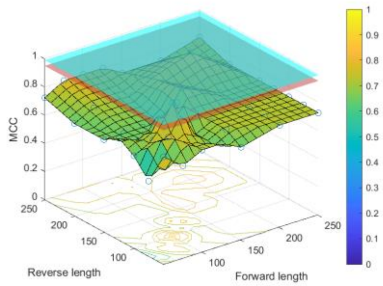
30X	40X	50X	
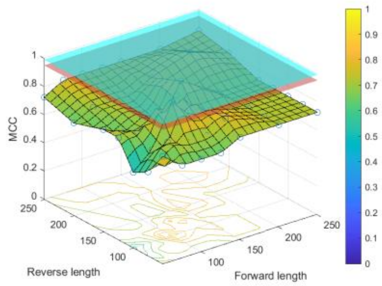	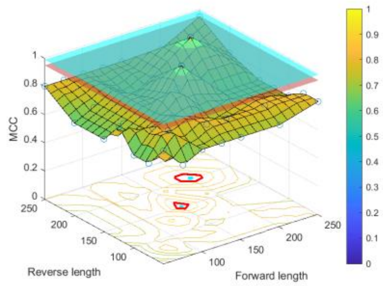	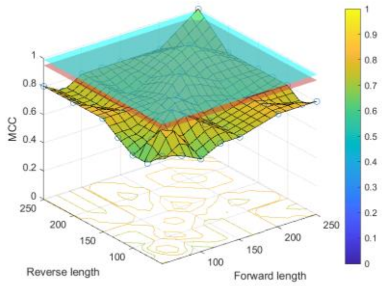	

Figure S53: Scenario 1 (No real-time analysis) – Serotype determination – Asymmetric read-length combinations – *N. meningitidis*

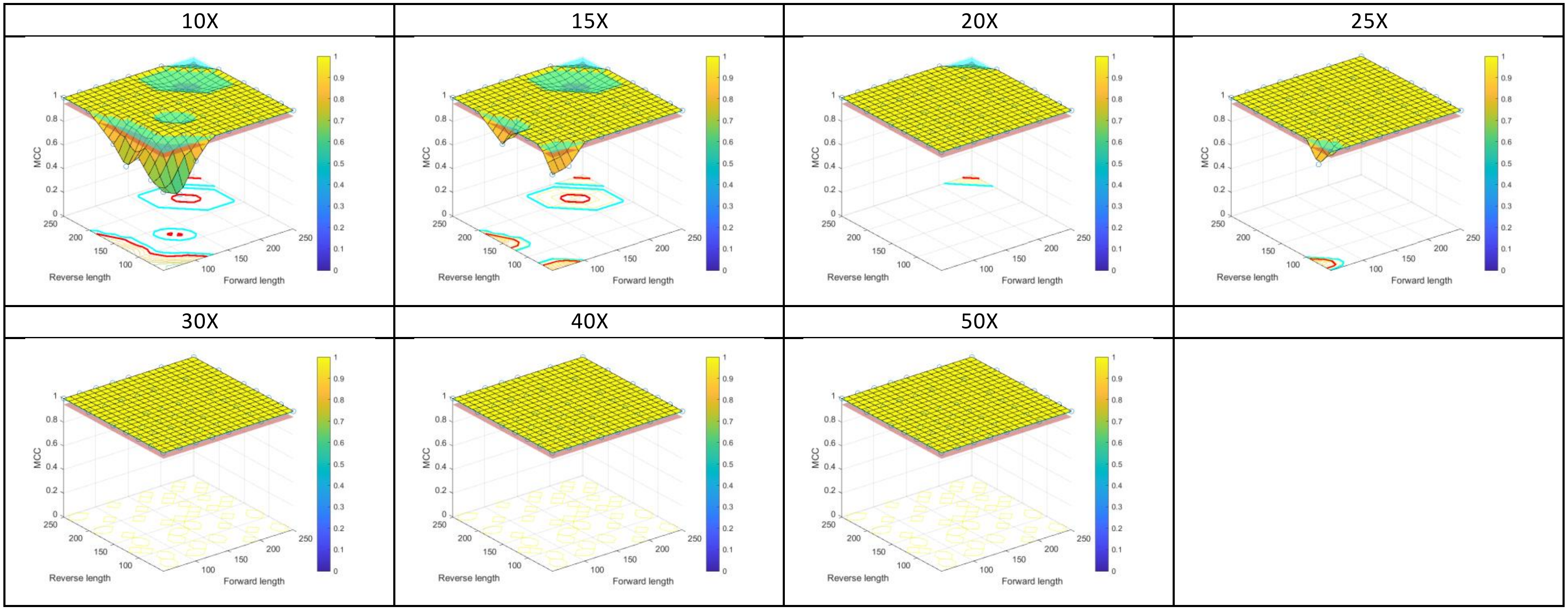

Figure S54: Scenario 2 (Real-time analysis) – 16S rRNA species confirmation – Asymmetric read-length combinations – *M. tuberculosis*

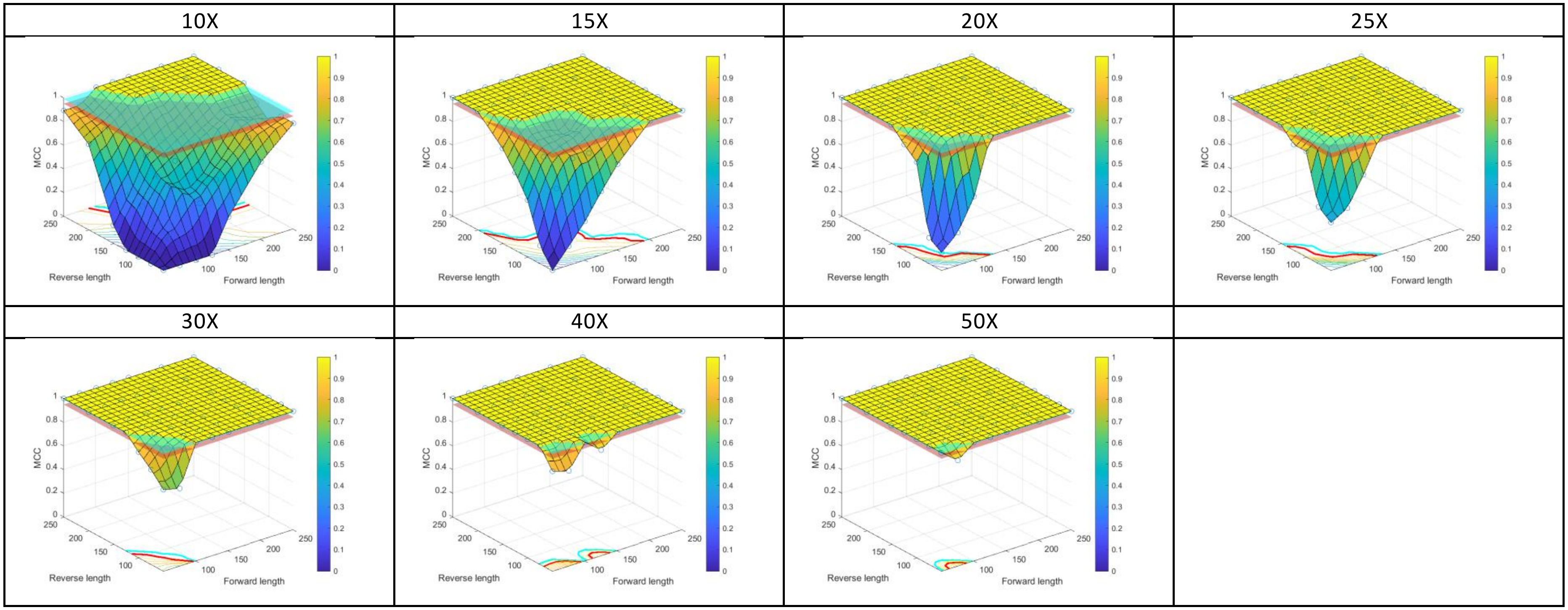

Figure S55: Scenario 2 (Real-time analysis) – Gene detection: AMR genes – Asymmetric read-length combinations – *E. coli*

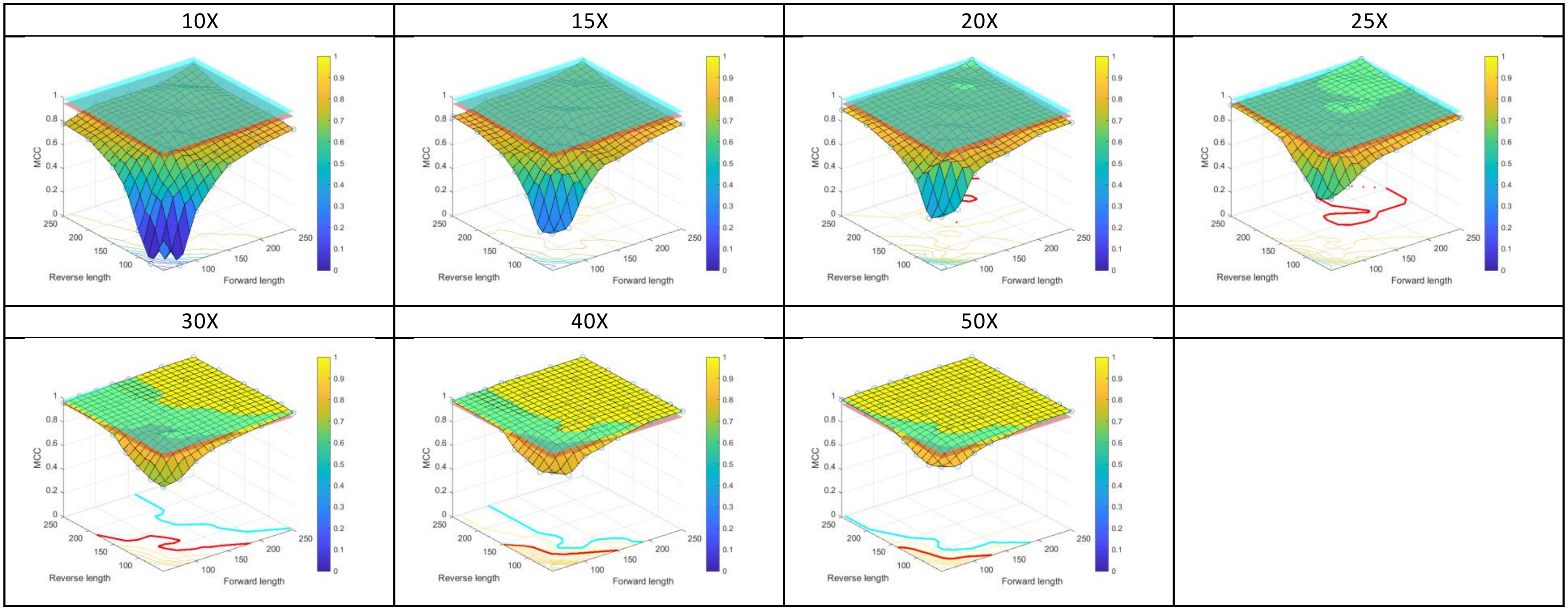

Figure S56: Scenario 2 (Real-time analysis) – Gene detection: Virulence genes – Asymmetric read-length combinations – *E. coli*

10X	15X	20X	25X
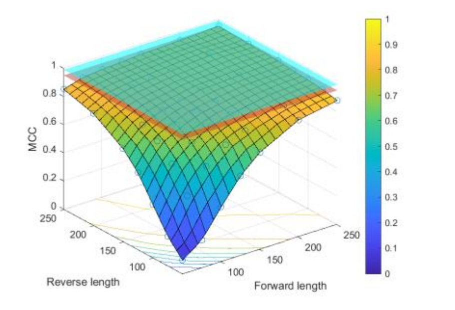	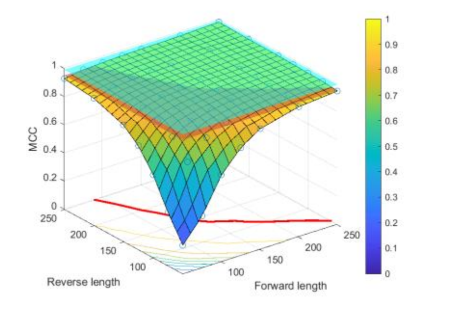	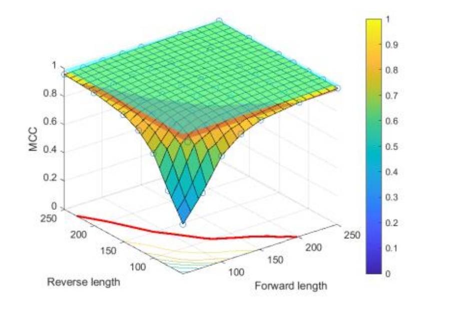	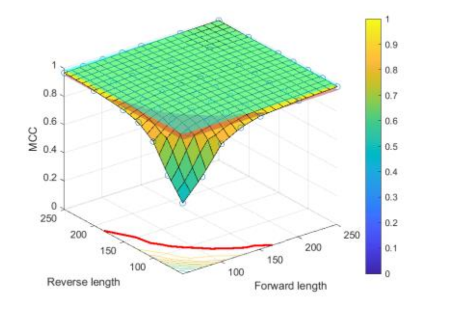
**30X**	**40X**	**50X**	
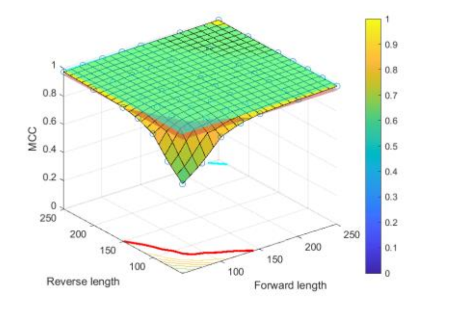	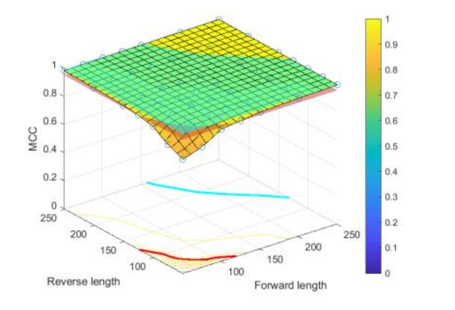	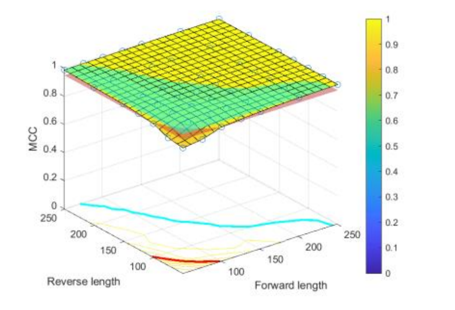	

Figure S57: Scenario 2 (Real-time analysis) – Gene detection: Virulence genes – Asymmetric read-length combinations – *N. meningitidis*

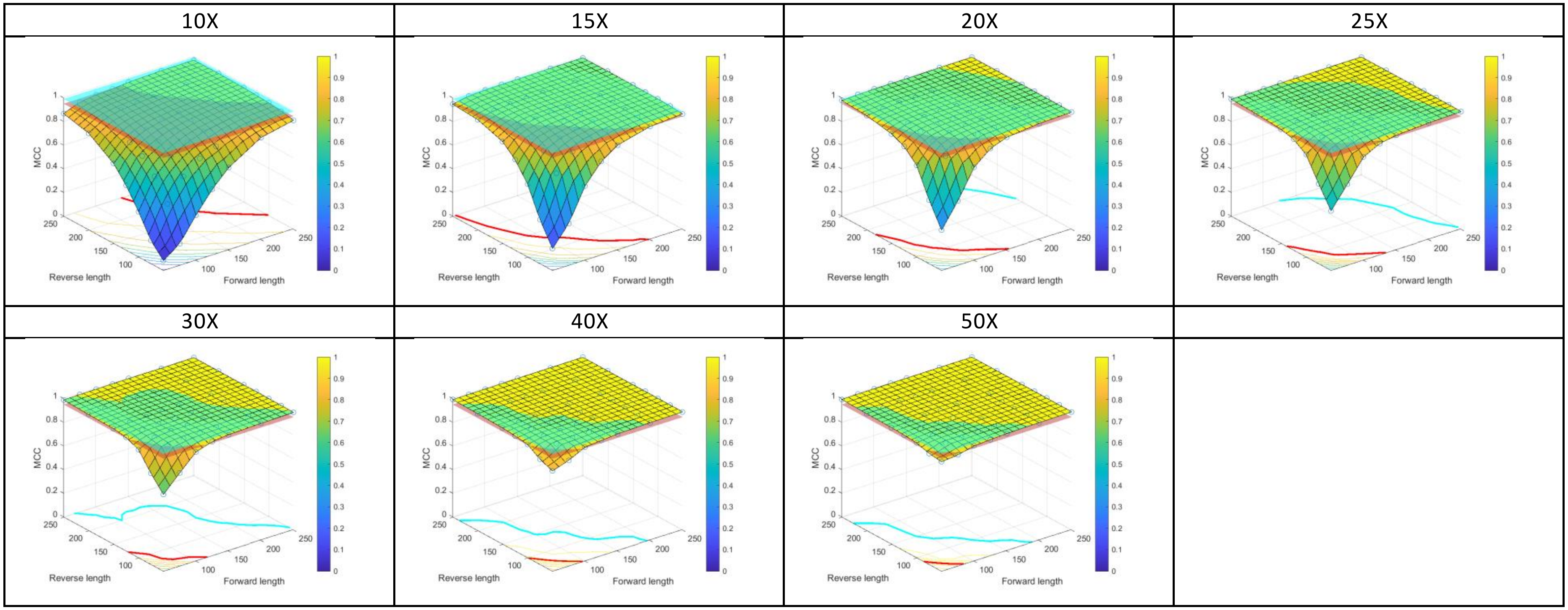

Figure S58: Scenario 2 (Real-time analysis) – PointFinder – Asymmetric read-length combinations – *E. coli*

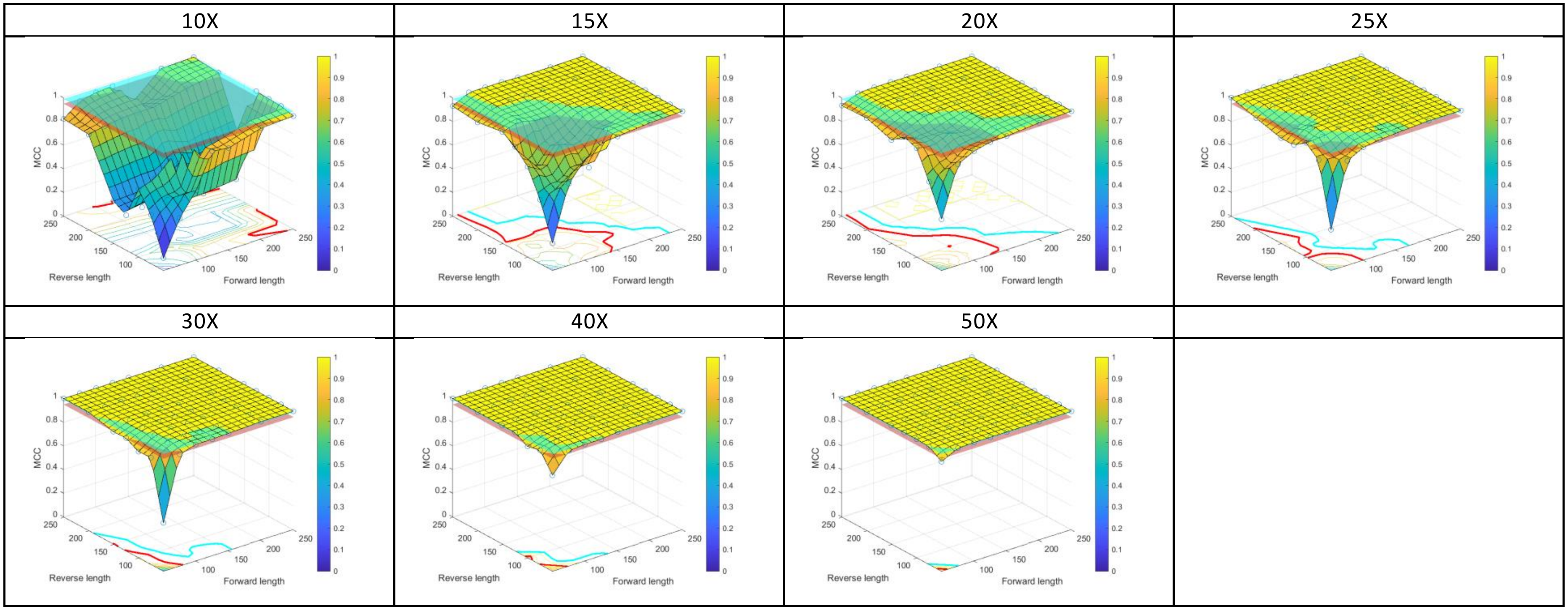

Figure S59: Scenario 2 (Real-time analysis) – PointFinder – Asymmetric read-length combinations – *M. tuberculosis*

10X	15X	20X	25X
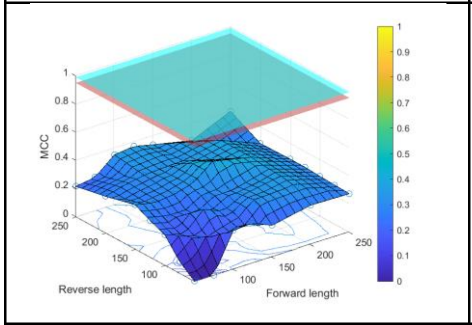	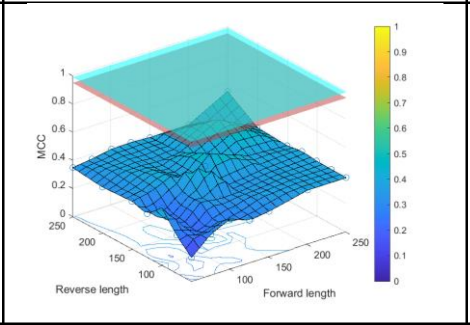	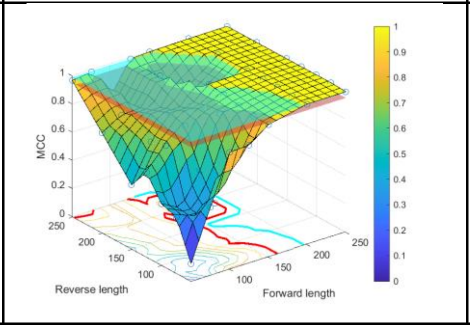	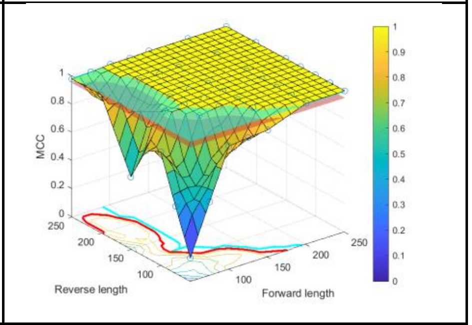
30X	40X	50X	
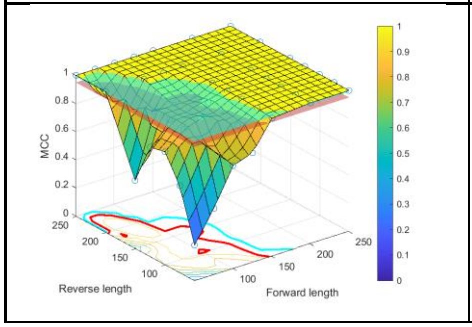	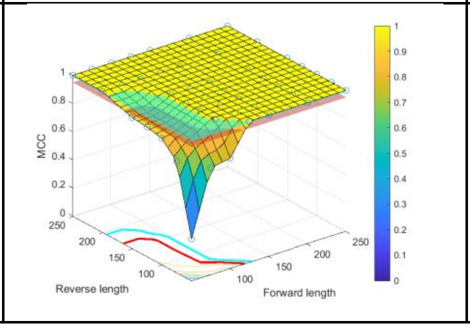	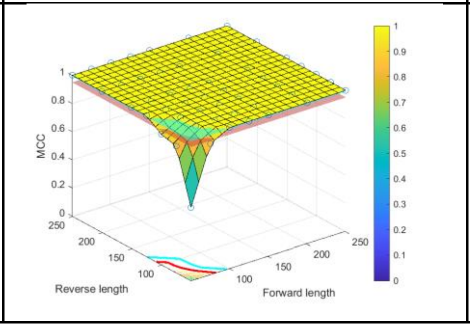	

Figure S60: Scenario 2 (Real-time analysis) – SNP-based antimicrobial resistance detection – Asymmetric read-length combinations – *M. tuberculosis*

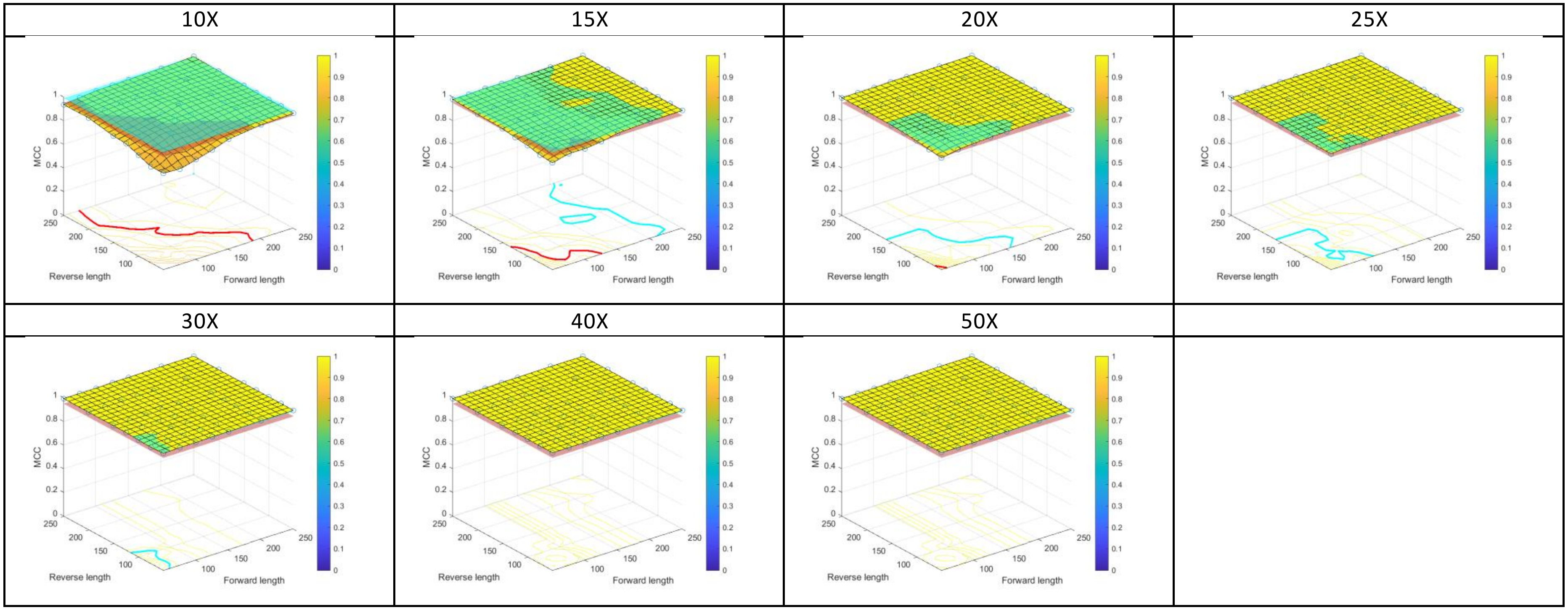

Figure S61: Scenario 2 (Real-time analysis) – Sequence typing – Asymmetric read-length combinations – *E. coli*

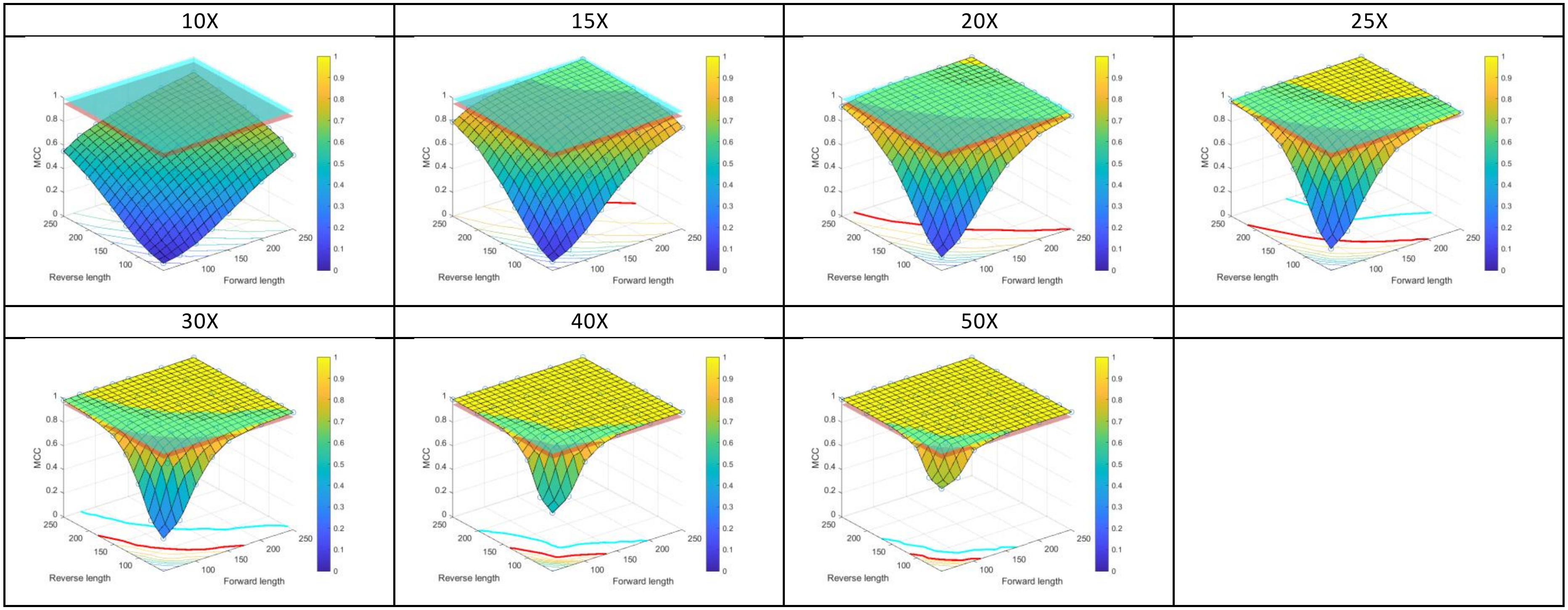

Figure S62: Scenario 2 (Real-time analysis) – Sequence typing – Asymmetric read-length combinations – *M. tuberculosis*

10X	15X	20X	25X
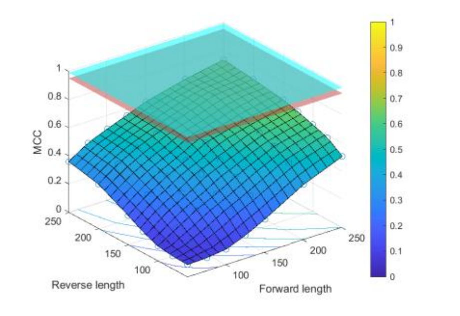	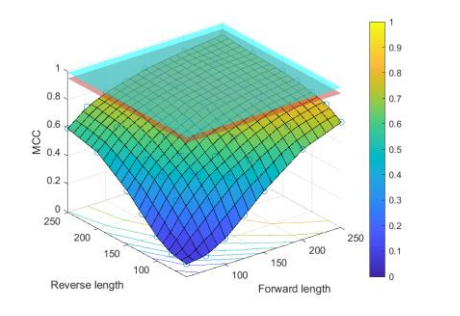	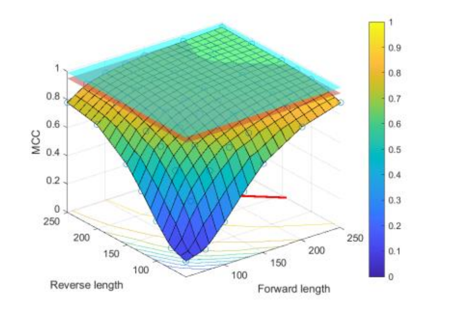	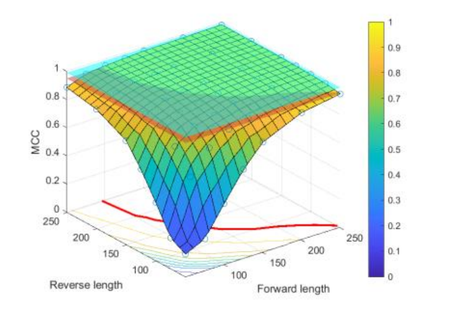
30X	40X	50X	
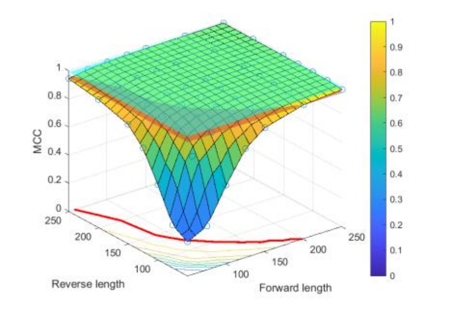	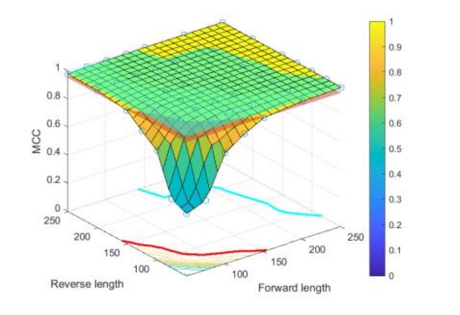	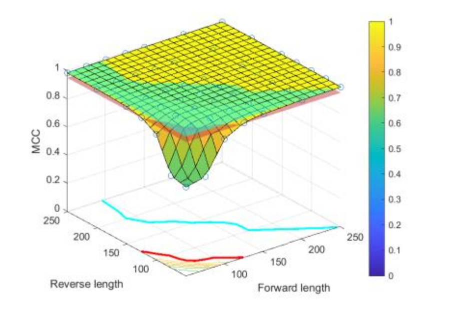	

Figure S63: Scenario 2 (Real-time analysis) – Sequence typing – Asymmetric read-length combinations – *N. meningitidis*

10X

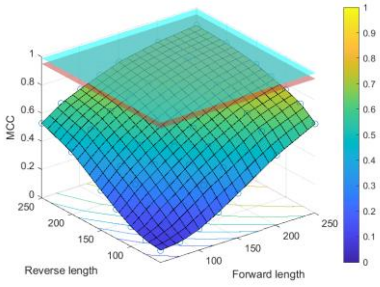

15X

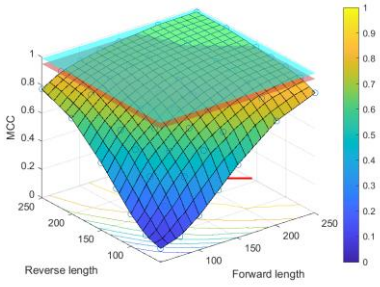

20X

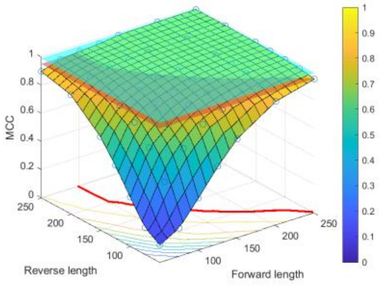

25X

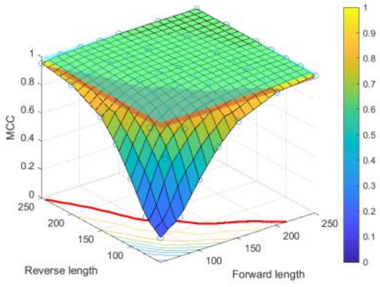

30X

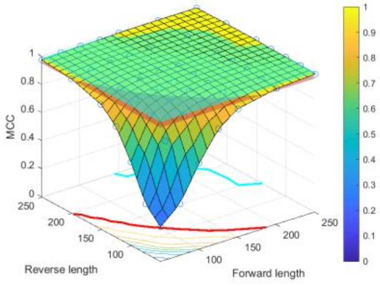

40X

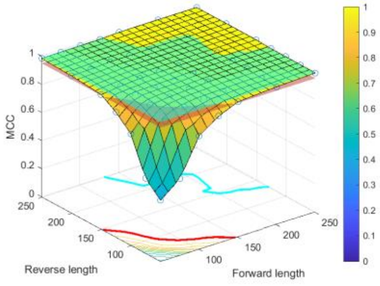

50X

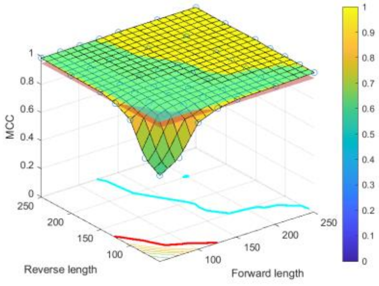

Figure S64: Scenario 2 (Real-time analysis) – Serotype determination – Asymmetric read-length combinations – *E. coli*

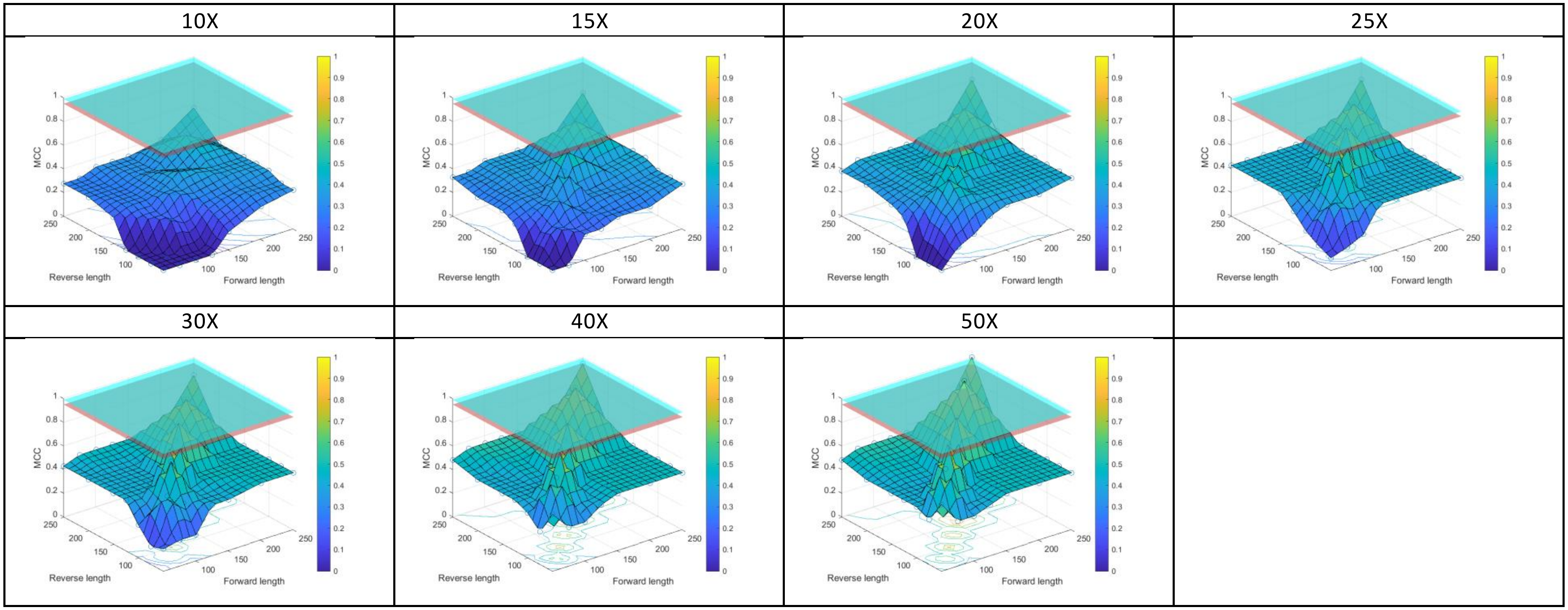

Figure S65: Scenario 2 (Real-time analysis) – Serotype determination – Asymmetric read-length combinations – *N. meningitidis*

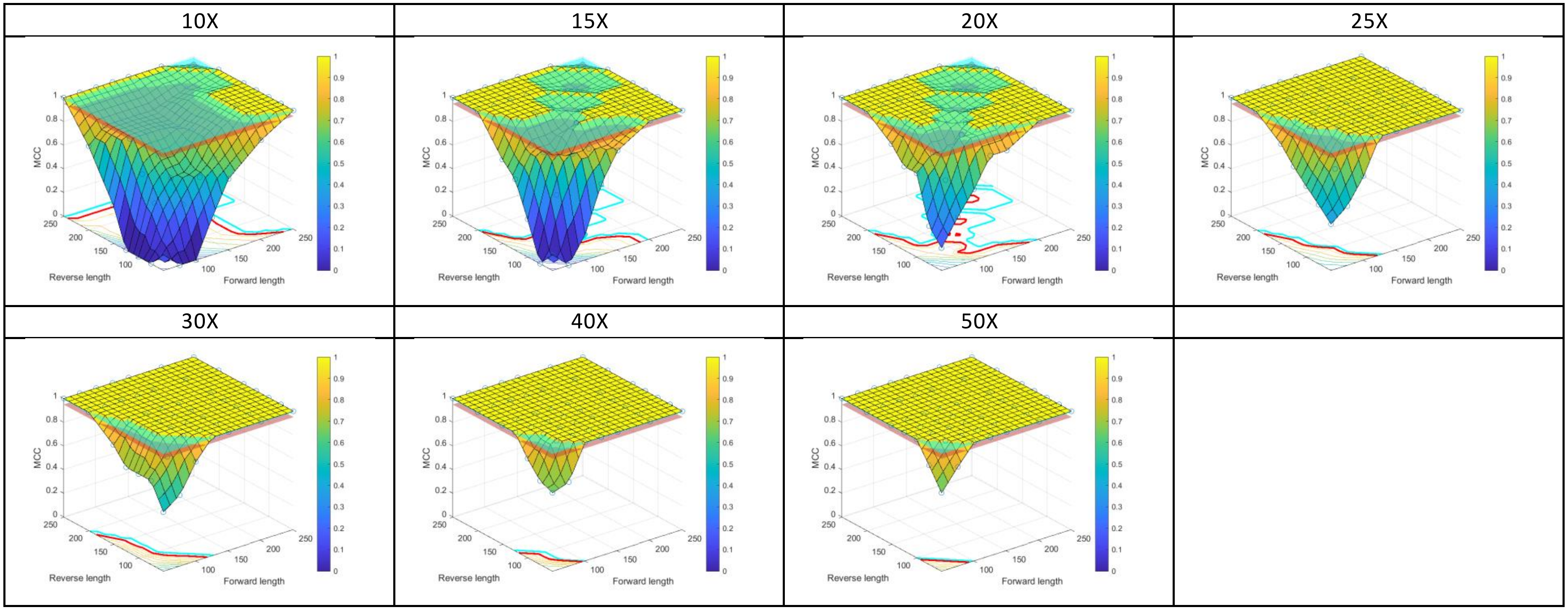

References

1. Bogaerts B, Winand R, Fu Q, Van Braekel J, Ceyssens P-J, Mattheus W, Bertrand S, De Keersmaecker SCJ, Roosens NHC, Vanneste K. 2019. Validation of a Bioinformatics Workflow for Routine Analysis of Whole-Genome Sequencing Data and Related Challenges for Pathogen Typing in a European National Reference Center: Neisseria meningitidis as a Proof-of-Concept. Front Microbiol 10.
2. Bogaerts B, Delcourt T, Soetaert K, Boarbi S, Ceyssens P-J, Winand R, Van Braekel J, De Keersmaecker SCJ, Roosens NHC, Marchal K, Mathys V, Vanneste K. 2021. A bioinformatics WGS workflow for clinical Mycobacterium tuberculosis complex isolate analysis, validated using a reference collection extensively characterized with conventional methods and in silico approaches . J Clin Microbiol https://doi.org/10.1128/jcm.00202-21.
3. Bogaerts B, Nouws S, Verhaegen B, Denayer S, Van Braekel J, Winand R, Fu Q, Crombé F, Piérard D, Marchal K, Roosens NHC, De KSCJ, Vanneste K. 2021. Validation strategy of a bioinformatics whole genome sequencing workflow for Shiga Toxin-producing Escherichia coli using a reference collection extensively characterized with conventional methods. Microb Genomics Accepted.
4. Bankevich A, Nurk S, Antipov D, Gurevich AA, Dvorkin M, Kulikov AS, Lesin VM, Nikolenko SI, Pham S, Prjibelski AD, Pyshkin A V., Sirotkin A V., Vyahhi N, Tesler G, Alekseyev MA, Pevzner P a. 2012. SPAdes: A New Genome Assembly Algorithm and Its Applications to Single-Cell Sequencing. J Comput Biol 19:455–477.
5. Chen L, Zheng D, Liu B, Yang J, Jin Q. 2016. VFDB 2016: Hierarchical and refined dataset for big data analysis - 10 years on. Nucleic Acids Res 44:D694–D697.
6. Jolley KA, Maiden MCJ. 2010. BIGSdb: Scalable analysis of bacterial genome variation at the population level. BMC Bioinformatics 11:595.
7. Feldgarden M, Brover V, Haft DH, Prasad AB, Slotta DJ, Tolstoy I, Tyson GH, Zhao S, Hsu CH, McDermott PF, Tadesse DA, Morales C, Simmons M, Tillman G, Wasilenko J, Folster JP, Klimke W. 2019. Validating the AMRFINder tool and resistance gene database by using antimicrobial resistance genotype-phenotype correlations in a collection of isolates. Antimicrob Agents Chemother 63:1–20.
8. Joensen KG, Tetzschner AMM, Iguchi A, Aarestrup FM, Scheutz F. 2015. Rapid and easy in silico serotyping of Escherichia coli isolates by use of whole-genome sequencing data. J Clin Microbiol 53:2410–2426.
9. Zhou Z, Alikhan NF, Mohamed K, Fan Y, Achtman M. 2020. The EnteroBase user's guide, with case studies on Salmonella transmissions, Yersinia pestis phylogeny, and Escherichia core genomic diversity. Genome Res 30:138–152.
10. Zankari E, Allesøe R, Joensen KG, Cavaco LM, Lund O, Aarestrup FM. 2017. PointFinder: A novel web tool for WGS-based detection of antimicrobial resistance associated with chromosomal point mutations in bacterial pathogens. J Antimicrob Chemother 72:2764–2768.
